# A structurally compact aqueous soluble oxypicolinium photocage with high photosensitivity†

**DOI:** 10.1039/d5sc00204d

**Published:** 2025-03-05

**Authors:** Komadhie C. Dissanayake, Mohammad K. I. Walid, Madelyn Austin, Emily A. Smith, Arthur H. Winter

**Affiliations:** a Department of Chemistry, Iowa State University Ames Iowa 50014 USA winter@iastate.edu

## Abstract

Photocages are protecting groups that can be unmasked with light irradiation and provide spatiotemporal control over the activity of a biomolecule. Ideally, photocages have high quantum yields for deprotection and good water solubility. Small photocages are preferred in applications where minimal biological perturbations of the photocage are desired or where large photocage structures stymie needed binding or activity, such as in the incorporation of unnatural photocaged amino acids into proteins. Here, we report *N*-methyl-3-oxypicolinium ester, by molecular weight the smallest photocage reported to date. The photocage is smaller (by MW) than even the parent nitrobenzyl photocage, but has improved properties, including being more water soluble as a result of its zwitterionic character, a higher quantum yield of release of 0.32 at pH 7 for release of the mediocre leaving group AcOH, and a superior chromophore within a single benzene ring (*λ*_max_ = 320 nm, *λ*_em_ = 402 nm) that matches the absorbance wavelength of the larger 7-hydroxy coumarin photocage. The zwitterionic character aids in water solubility, red-shifts the chromophore absorption and emission by raising the HOMO and lowering the LUMO, and improves the quantum yield of release. Cell studies show that the photocage crosses the HEK293 cell membrane and shows no observable toxicity (trypan blue exclusion assay, 25 μM), while mechanistic studies indicate a singlet photoheterolysis mechanism that is supported by ultrafast transient absorption spectroscopy, oxygen sensitivity studies, computational investigations, and photoproduct analysis.

## Abstract 2

Photocages are protecting groups that can be unmasked with light irradiation and provide spatiotemporal control over the activity of a biomolecule.

## Graphical abstract

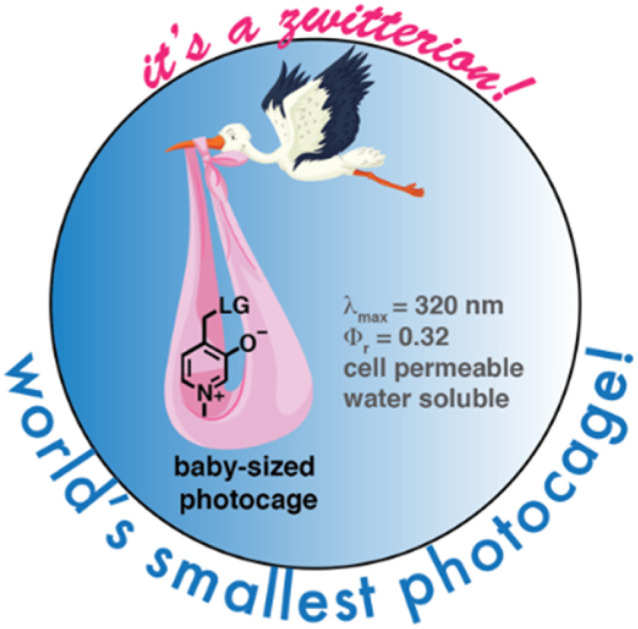

## Introduction

Photocages are photoremovable protecting groups (PPGs) that are synthetically attached to a chemical cargo, temporarily hindering its biological activity. The covalent bond between the photocage and the cargo is broken upon light irradiation, releasing the cargo and restoring its biological activity. This irradiation-dependent release of substrates is useful in fields such as biological sciences, synthetic chemistry, and material sciences due to the unmatched ability of light to provide spatiotemporal control. However, photocages require an absorbance above 300 nm to prevent irradiation with deep-UV light that can cause sub-cellular damage and phototoxicity. Other attractive properties in a photocage are a high quantum yield of release (*Φ*_r_), solubility in aqueous media, thermal stability in the dark, cellular permeability, and biocompatibility.^1^ With the increasing demand for photocages for biological applications, there is a need for new classes of photocages that improve their fundamental properties. Many recent advances have resulted in promising photocages that function at long wavelengths in the visible and NIR region^2–5^ however, hydrophilicity and size of the photocages are often compromised while tuning the absorption towards the biological window by increasing the conjugations, while light penetration into tissue is not important for *in vitro* applications.

The smallest, earliest introduced, and most widely used photocages are based on the *ortho*-nitrobenzyl (oNB) moiety. They are valued for their smaller size, commercial availability, functional group tolerance, and ease of synthesis.^6^ However, the parent oNB photocage has limited water solubility, a low *Φ*_r_, and a toxic nitrosoarene byproduct. Other related photocages are the phenacyl and coumarin photocages.

**Fig. 1:**
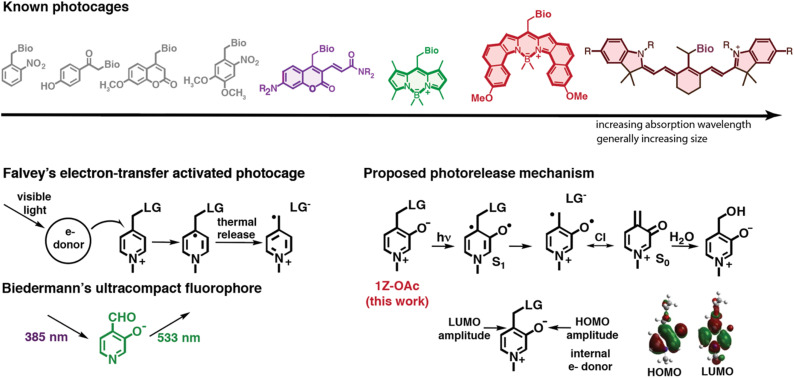
(Top) Commonly used photocages. (Bottom left) Falvey's electron transfer activated picolinium ion photocage and Biedermann's compact oxypyridine fluorophore. (Bottom right) Oxypicolinium zwitterionic photocage (this work) showing proposed photoreaction mechanism and orbital diagrams for HOMO and LUMO.

Relevant to this study, a related series of novel photocages have been developed by Falvey and coworkers^7–9^ (Fig. 1), which has a picolinium ion as the core, making the photocage soluble in water. However, these PPGs have yet to be used in a biological context because they require a photoreductant that is irradiated to start a photoinduced electron transfer reaction to the picolinium PPG that initiates photorelease. Inspired by the ultracompact HINA fluorophore by Biedermann and coworkers,^10^ which packages a potent green-light-emitting chromophore in a single aromatic ring, as well as Falvey's picolinium photocage; we synthesized the photocage *N*-methyl-3-hydroxypicolinium ester (1-OAc), which exists as a zwitterion above pH 4. The design starts with Falvey's picolinium PPG and marries it with Biedermann's ultracompact chromophore within a single compact PPG where the “electron donor” oxyanion is, in a sense, incorporated as part of the chromophore rather than added as a separate agent.

We find that this new zwitterionic PPG has advantageous properties. Notably, it has an absorption *λ*_max_ that is the same as the larger 7-hydroxycoumarin PPG^11^ and with a violet emission (*λ*_em_ = 402 nm). It also has an excellent quantum yield of release of *Φ*_r_ = 0.32 for release of the mediocre leaving group AcOH, measured in pH 7 phosphate buffer under ambient conditions. Oxygen sensitivity studies showed that the release is not affected by oxygen, suggesting a singlet photorelease mechanism, which was further corroborated by transient absorption experiments and product studies. Cell studies confirmed the ability of the photocage to permeate the cell membrane. These findings collectively demonstrate that this oxypicolphotoinium photocage combines a compact potent chromophore within a single benzene ring that mimics the optical absorption of the much larger hydroxy coumarin PPG, yet improving on its photosensitivity, aqueous solubility, and cell permeability, making it a unique addition to the photocaging arsenal.

## Results and discussion

### Synthesis and characterization

Synthetic pathways for preparing the photocages are shown in Scheme S1.† The *N*-methyl-3-hydroxypicolinium ester salt (1-OAc) was prepared by first protecting the hydroxy group of the commercially available methyl-3-hydroxyisonicotinate with tert-butyldimethylsilyl chloride followed by the reduction of the ester group to a primary alcohol. Acetic acid was then attached to the alcohol *via* DCC coupling. Deprotection of the phenolic alcohol was carried out with TBAF, and in the final step, trimethyloxonium tetrafluoroborate was used to methylate the N on the pyridine. 1-OAc was converted to the zwitterion by deprotonating the phenolic OH to give 1z-OAc. To validate our hypothesis of neighboring oxide stabilizing the carbocation we also synthesized photocages bearing OMe (2-OAc) and H (3-OAc) at the 3rd position.

**Fig. 2:**
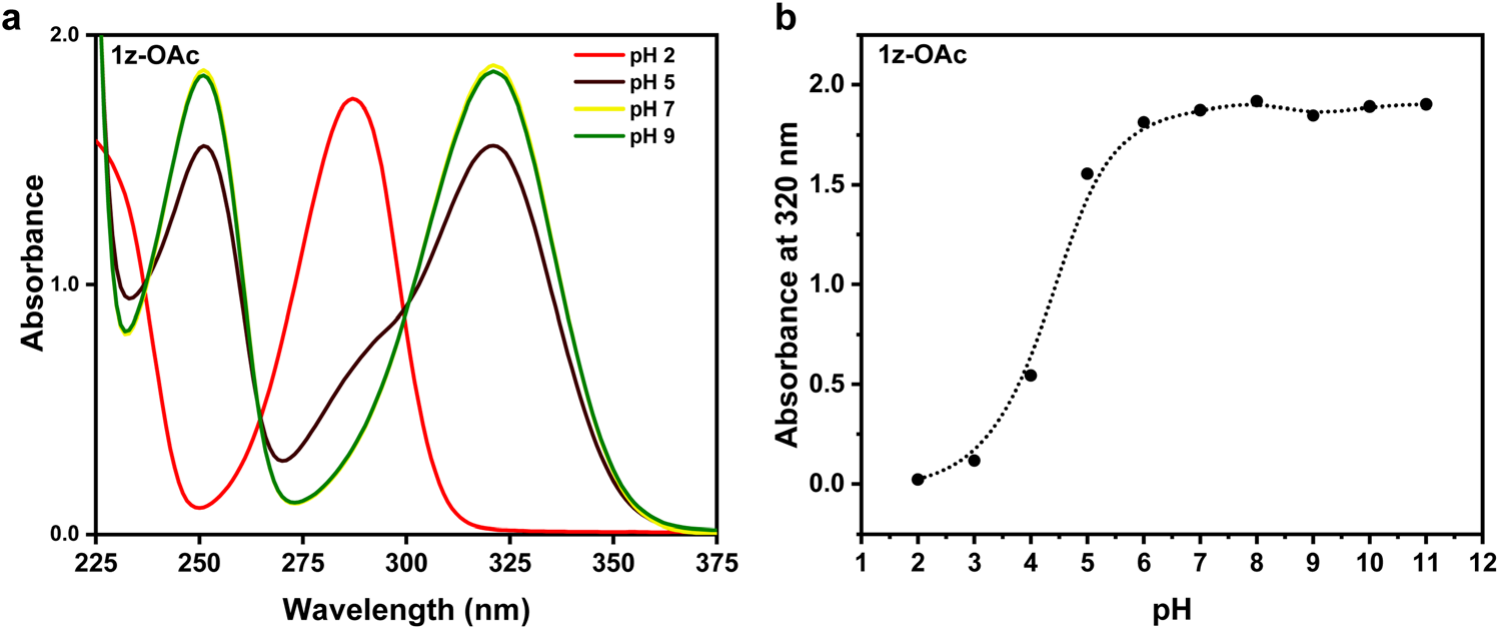
(a) pH-dependent absorption profile of 1-OAc. (b) Absorbance at 320 nm as a function of pH, showing the zwitterionic character above pH = 4.

As expected of a zwitterion, compound 1z-OAc showed full water solubility up to 5 mM, the maximum concentration tried. The compound is also soluble in other polar solvents such as methanol, acetonitrile, nitromethane, and dimethyl sulfoxide. Experiments described herein were conducted in aqueous media. Absorbance spectra of 1-OAc were recorded in a series of buffers ranging from pH 2 to pH 11, as shown in Fig. 2. In pH > 5, 1-OAc is in its zwitterionic form 1z-OAc, whereas, in lower pH, 1-OAc is the dominant species.

**Fig. 3:**
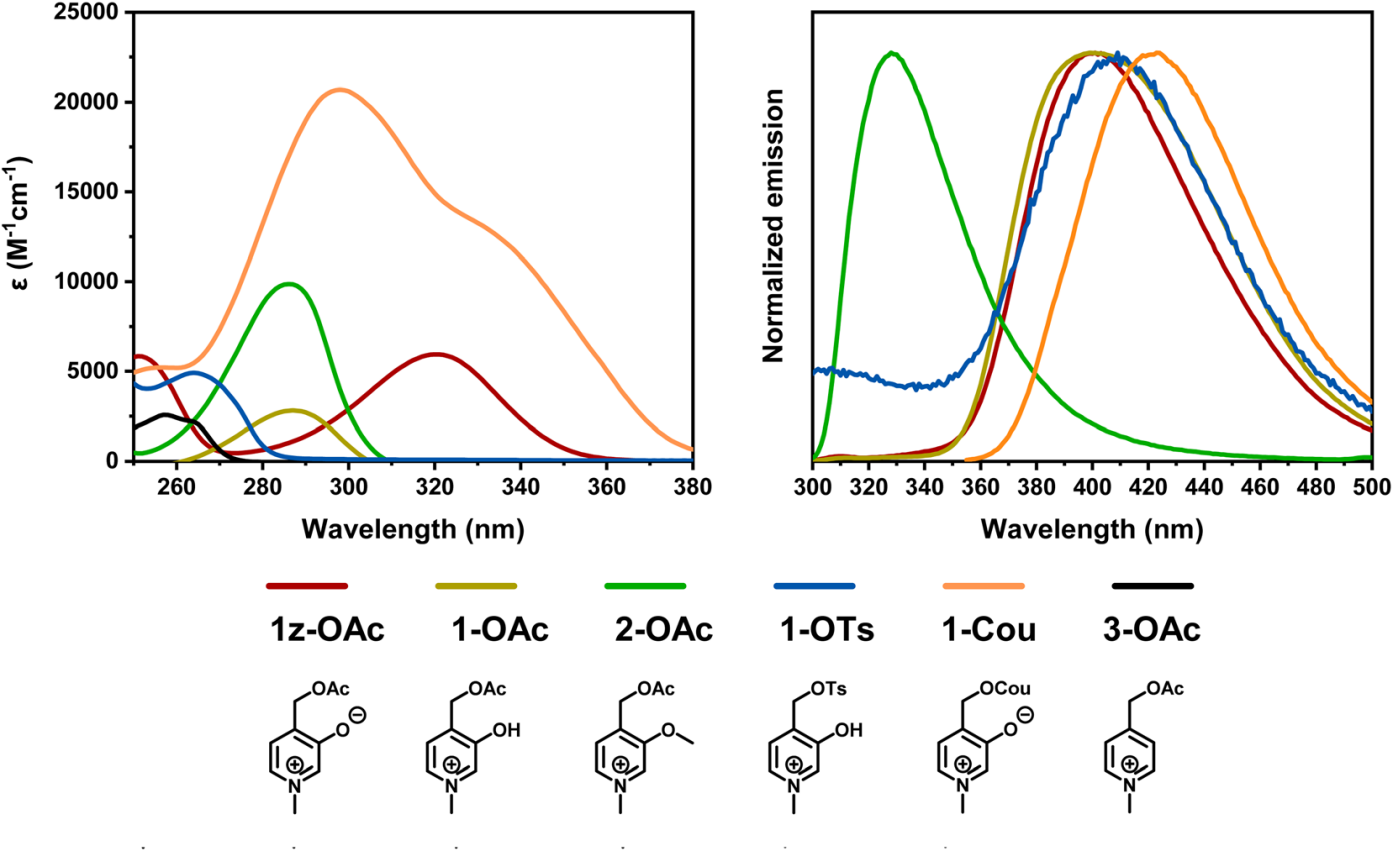
Absorption and emission profiles of the photocages synthesized in pH = 7 phosphate buffer.

Hammett *σ*_p_ values can be used to compare the electron-donating character of substituents. The substituents of interest are O^−^, OH, OMe, and H, which have *σ*_p_ Hammett constants of −0.81, −0.37, −0.27, and 0.00, respectively. Fig. 3 summarizes the absorption and emission profiles of **1z-OAc**, **1-OAc**, **2-OAc**, and 3-OAc.The absorption maxima ranged from 221 nm to 320 nm, with the lowest observed for 3-OAc and the highest reported for 1z-OAc. While there is a discernible trend in the absorption maxima, indicating that a more electron-donating substituent leads to a redshift in the absorption maxima, the substituents OH (1-OAc) and OMe (2-OAc) did not significantly alter the UV-vis absorption spectra. This lack of absorption distinction may be attributed to their closely matched electron-donating abilities (*σ*_p_ of −0.27 and −0.37). In contrast, 1-OAc and 2-OAc displayed significantly shifted emission in the emission spectra, approximately 70 nm apart. Surprisingly, 1z-OAc and 1-OAc didn't differ substantially in the emission maxima. Additionally, 3-OAc had extremely weak fluorescence.

### Improved photocage efficiency with better *ortho* donors

**Fig. 4:**
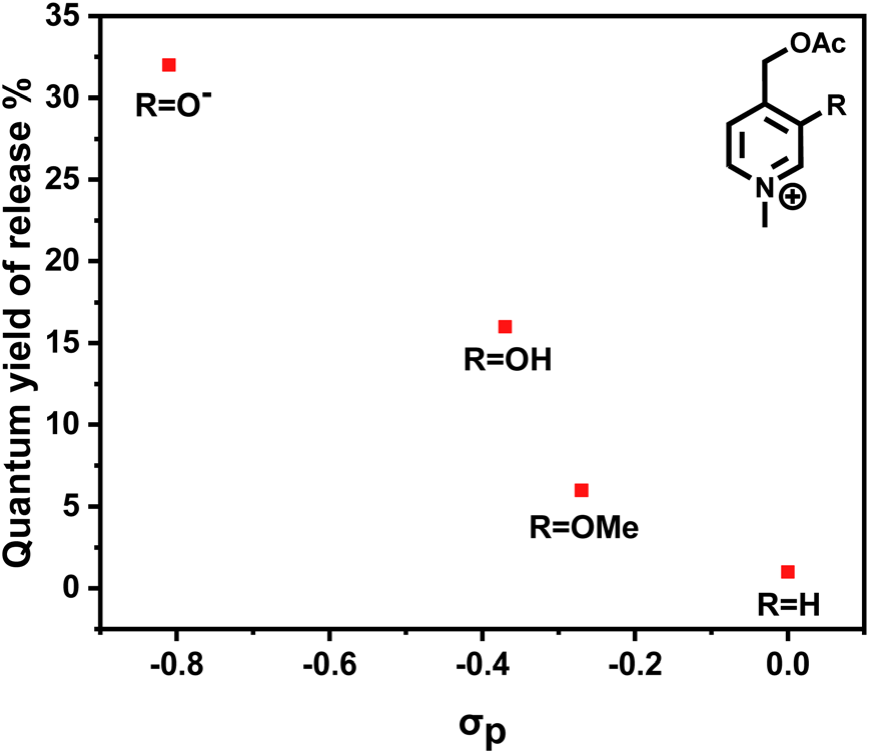
Correlation of quantum yield of release with the electron-donating ability of the substituent *R*.

The *Φ*_r_ of the synthesized photocages was measured by actinometry using ^1^H NMR following the photoreleased acetic acid (see ESI-Section 3.1†). The reported value for each compound in Table 1 averages three independent trials. Zwitterionic 1z-OAc exhibited the highest *Φ*_r_, while the lowest *Φ*_r_ was observed when the neighboring group was H (3-OAc). The increase in *Φ*_r_ achieved by incorporating a strong electron-donating group amounts to an impressive 32-fold enhancement. The experimental results were plotted against Hammett *σ*_p_ values in Fig. 4. This reveals a correlation between the electron-donating ability of the neighboring groups and the obtained *Φ*_r_. Furthermore, DFT calculations were conducted to determine the energy barriers associated with the C–O heterolysis for the triplet states of the photocages, which were found as 9.1, 12.7, 12.6, and 13.4 kcal mol^−1^ for 1z-OAc, 1-OAc, 2-OAc, and 3-OAc respectively with a general trend of electron donating groups lowering the energy barrier. These experimental and theoretical results support the hypothesis of electron donating groups in the neighboring position participating in stabilizing the cation, facilitating photo heterolysis.

**Table 1:** Summary of photophysical properties of photocages

Compound	pH	*λ*_max, ab_ (nm)	*λ*_max, em_ (nm)	*Φ*_r_ a (%)	ε × 10^3^ (M^−1^ cm^−1^)	*Φ*_r_·*ε* (M^−1^ cm^−1^)
1z-OAc	7	320	402	32^b^	6.0	1920
1-OAc	2	285	401	16^b^	3.4	544
2-OAc	7	286	330	6^b^	10.0	600
3-OAc	7	221	n.d.	1^c^	2.2	22
1-OTs	7	265	409	4^c^	4.9	196

a *Φ*_r_ were obtained using quantitative ^1^H-NMR following the growth of AcOH or OTs in the corresponding buffer solution with ferrioxalate as the actinometer.

b Samples were irradiated using four 6-inch 300 nm bulbs in the air without purging.

c Samples were irradiated using four 254 nm bulbs in the air without purging. n.d.- not detected.

### 1z-OAc is thermally stable in the dark

The stability of compound 1z-OAc at pH 7 was evaluated by keeping the sample in the dark, followed by quantifying the release of acetic acid by NMR over a period (ESI-Section 3.4†). After 24 hours, only 1% of 1z-OAc was found to be hydrolyzed. This shows the stability of the photocage 1z-OAc in the aqueous media at pH 7, an essential parameter for photocages to be used in biological studies. Furthermore, a photocage incorporating a well-established good leaving group, para toluene sulfonate (1-OTs), was synthesized. The calculated energy barrier for 1-OTs exhibited the lowest among the newly synthesized compounds at 2.4 kcal, signifying a higher release rate. However, in contrast to 1-OAc, which displayed a pH-dependent deprotonation of the phenolic OH, 1-OTs exhibited no discernible change in absorption spectra, suggesting an inability to convert to the zwitterionic form (Fig. S12†). The *Φ*_r_ measured at 254 nm for 1-OTs was 8-fold lower than 1z-OAc. This lower *Φ*_r_ may be ascribed to the inability to form a zwitterion, probably arising from an intramolecular hydrogen bonding between the phenolic H and sulfonyl oxygen. This interaction may hinder deprotonation and impede solvent separation of the ion pair, promoting recombination and lowering the *Φ*_r_

Though not precisely matched in size, a photocage bearing resemblance in absorbance to 1z-OAc (320 nm) is 7-hydroxy coumarin-based photocage at physiological pH (325 nm).^11^ However, 1z-OAc exhibits superior performance across several metrics. Notably, it demonstrates remarkable water solubility and higher *Φ*_r_ (0.32 at 300 nm *vs.* 0.025 at 365 nm). This enhancement underscores 1z-OAc's potential as an advanced photocage for biological applications.

### Photoheterolysis from singlet excited state

Photorelease of 1z-OAc was monitored using NMR, UV-vis, and fluorescence spectroscopies. Results from UV-Vis and fluorescence studies are shown in ESI-Section 4.†^1^H NMR measurements upon irradiation showed the emergence of *N*-methyl-3-oxy-4-methanol-pyridinium (1-OH), which was confirmed by comparing the NMR, HRMS, and retention time in LC-MS with 1-OH (ESI-Section 3.5†). 1-OH is a solvent adduct that is commonly observed in photocages that undergo photoheterolysis,^4,5^ suggesting that the major pathway of photorelease might be a photo S_N_1-type reaction. This can further be supported by the lack of evidence for forming 1-H, which has been the major photoproduct in homolytic cleavage reported by Falvey and co-workers.^7–9^

To investigate the mechanism further, oxygen dependent photorelease studies were conducted. The photorelease under air is within 10% of the rate under nitrogen. Zhang and coworkers reported increased photorelease for a photocage based on a pyridinium ion, attributing the increased rate to a side oxidation reaction.^12^ However, in contrast to their observation, in this study, the difference between the rates with and without oxygen is insignificant (10 μM s^−1^*vs.* 9 μM s^−1^ respectively), and in the photo product analysis, 1-COOH oxidation product was not observed; only 1-CHO was detected as a trace green-light-emissive byproduct (Fig. S7†). These observations could indicate that the side oxidation reaction might not significantly contribute to the release of acetic acid, potentially due to the high photorelease efficiency of 1z-OAc.

**Fig. 5:**
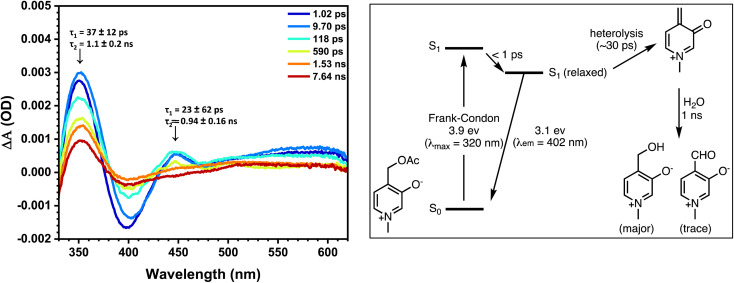
(Left) Transient absorption spectra of 1z-OAc of selected time delays between 1.02 ps–7.64 ns in pH 7 buffer solution with an excitation wavelength of 320 nm. (Right) Jablonski diagram with the proposed photoreaction mechanism.

Femtosecond-resolved transient absorption studies (Fig. 5) were carried out to obtain more insight into the mechanism of release, and the results were compared with the calculated TD-DFT spectra (Fig. S22†). The spectra show transients having lifetimes of 20–40 ps with an absorption of about 350 nm and a longer-lived transient absorbing ∼450 nm having a lifetime of ∼1 ns. Based on comparisons with TD-DFT, computed spectra of candidate intermediates support a singlet photoheterolysis taking place in 20–30 ps to generate a cation having a lifetime of ∼1 ns. Based on these data, the proposed Jablonksi diagram is shown in Fig. 5 (right).

### Photorelease in cells and cell toxicity evaluation

**Fig. 6:**
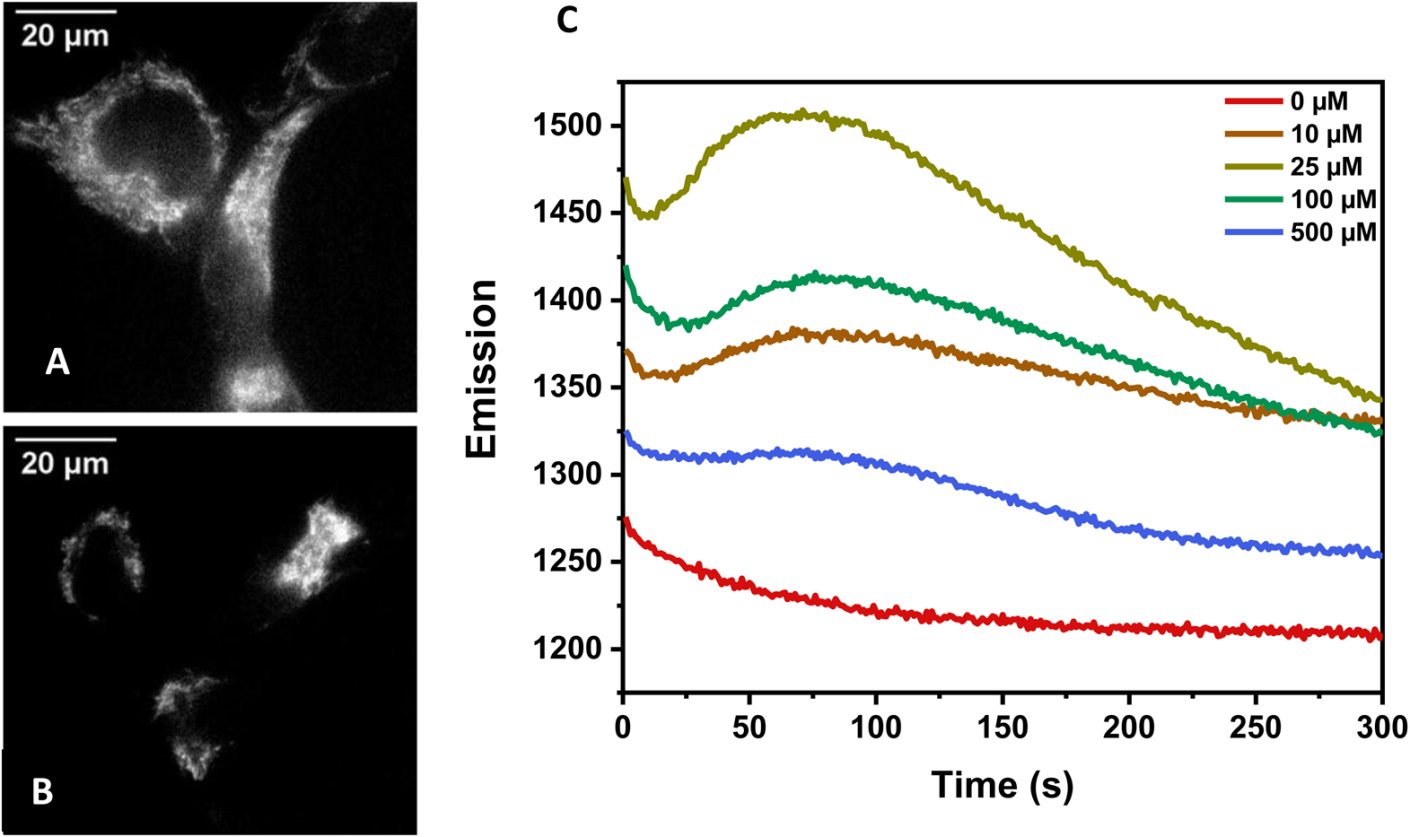
(A) HEK293 cells after incubation with 1z-OAc (25 μM) (B) HEK293 cells without 1z-OAc. (C) Graph showing the changes in fluorescence intensity monitored at 400–450 nm over time for live cell fluorescence imaging experiments. The change in emission of fluorescence is shown when HEK293 cells being treated with 10 μM, 25 μM, 100 μM and 500 μM of compound 1z-OAc were irradiated continuously with 365 ± 17 nm light and imaged at 400 ± 25 nm. Note that the *y*-axis of C does not start at zero and that the emission goes up and then down above 25 μM.

To test the cell permeability HEK293 cells were incubated with 1z-OAc at different concentrations and monitored with fluorescence microscopy to evaluate the cell permeability of the new photocage. The incubated whole live cells were irradiated with 365 ± 17 nm and imaged at 400–450 nm. A significant difference was observed in the sample incubated with 1z-OAc, shown in Fig. 6A and B. Additionally, an increase in the fluorescence intensity in the incubated cells with concentration was observed, indicating the accumulation of 1z-OAc inside the HEK293 cells, which is shown in Fig. 6C. For cellular studies, 1-Cou was synthesized, but the difference in fluorescence between the photocaged and photoreleased forms was too small to be confident that the small change in cellular fluorescence was due to photorelease (see ESI†). Additional studies for cytotoxicity of 1z-OAc with Trypan blue exclusion assay showed 95% cell viability with 25 μM incubation, so the photocage does not have observable cellular toxicity at these concentrations. Above 25 μM toxicity was observed, which may explain the decrease in fluorescence (Fig. 6C) at concentrations >25 μM.

## Conclusions

We have synthesized a photocage that shows solubility and stability in water, which also has excellent release efficiency in aqueous media compared to the parent nitrobenzyl photocage. The photocage shows surprising cell permeability for a zwitterion and no observed toxicity at 25 μM concentration. In conclusion, this new tiny photocage is highly water-soluble, cell membrane-permeant, non-toxic, thermally stable, and highly photoreactive.

Thus, it offers a competitor to the parent *o*-nitrobenzyl PPG as a minimally perturbing protecting group. They are roughly the same size (1 benzene), although the oxypicolinium has a slightly smaller MW (MW = 123 g mol^−1^ for nitrobenzene; 109 g mol^−1^ for oxypicolinium). Although the parent nitrobenzyl photocage has its advantages (easy synthesis, ‘just works’ as a plug and play structure, long storied history for chemical biology problems), it is inferior to the oxypicolinium PPG from the perspective of absorption profile and photosensitivity. Thus, the principle advantages to this new oxypicolinium PPG compared to *o*-nitrobenzyl PPG are: (1) a red-shifted absorption and emission of this zwitterionic photocage, beyond the most damaging deep-UV wavelengths; (2) a higher photosensitivity (>3× more photosensitive comparing *Φ*_r_*ε* values); (3) better water solubility from its zwitterionic structure; (4) it makes an unreactive molecular byproduct and not a reactive/toxic nitrosoarene byproduct/polymer. These features make it a nice addition to the minimally-perturbing photocaging arsenal.

## Data availability

The data supporting this article have been included as part of the ESI.†

## Author contributions

K. C. D. developed, synthesized, and characterized the photocages and photolysis experiments. M. W. conducted the cell studies. M. A. conducted the transient spectroscopic studies.

## Conflicts of interest

There are no conflicts to declare.

## Supplementary Material

SC-OLF-D5SC00204D-s001
