# Supplementary material for: A structurally compact aqueous soluble oxypicolinium photocage with high photosensitivity
Source: Chem Sci. 2025 Mar 5;16(15):6418–24. doi: 10.1039/d5sc00204d (PMC11912023; doi:10.1039/d5sc00204d)
Supplement: SC-016-D5SC00204D-s001 [file SC-016-D5SC00204D-s001.pdf]

Supplementary Information (SI) for Chemical Science.
This journal is © The Royal Society of Chemistry 2025

Supporting Information

A Structurally Compact Aqueous Soluble Photocage with High Photosensitivity

Komadhie C. Dissanayake, Mohammad K. I. Walid, Madelyn Austin, Emily A. Smith, Arthur H. Winter*

Iowa State University,

AUTHOR INFORMATION

*Corresponding author: Prof. Arthur Winter

E-mail: winter@iastate.edu

Contents

1 General Information 4

2 Compound Preparation 5

2.1 Synthetic scheme 5

2.2 Synthetic procedures and characterization.. 5

2.2.1 Synthesis of methyl 3-((tert-butyldimethylsilyl)oxy)isonicotinate (a) 5

2.2.2 Synthesis of (3-((tert-butyldimethylsilyl)oxy)pyridin-4-yl)methanol (b) 6

2.2.3 Synthesis of (3-((tert-butyldimethylsilyl)oxy)pyridin-4-yl)methyl acetate (c).................... 7

2.2.4 Synthesis of (3-methoxypyridin-4-yl)methyl acetate (2-c).. 7

2.2.5 Synthesis of (3-((tert-butyldimethylsilyl)oxy)pyridin-4-yl)methyl 2-oxo-2H-chromene-3-carboxylate (c-Cou) 8

2.2.6 Synthesis of (3-((tert-butyldimethylsilyl)oxy)pyridin-4-yl)methyl 4-methylbenzenesulfonate (c-OTs).. 8

2.2.7 Synthesis of pyridin-4-ylmethyl acetate (3-c).. 9

2.2.8 Synthesis of (3-hydroxypyridin-4-yl)methyl acetate (d).. 10

2.2.9 Synthesis of (3-hydroxypyridin-4-yl)methyl 4-methylbenzenesulfonate (d-OTs) 10

2.2.10 Synthesis of 4-(acetoxymethyl)-3-hydroxy-1-methylpyridin-1-ium (1-OAc) 11

2.2.11 Synthesis of 4-(acetoxymethyl)-3-methoxy-1-methylpyridin-1-ium (2-OAc) 11

2.2.12 Synthesis of 3-hydroxy-1-methyl-4-((tosyloxy)methyl)pyridin-1-ium tetrafluoroborate (1-OTs) 12

2.2.13 Synthesis of 4-(acetoxymethyl)-1-methylpyridin-1-ium tetrafluoroborate (3-OAc)........ 12

2.2.14 Synthesis of 3-hydroxy-1-methyl-4-(((2-oxo-2H-chromene-3-carbonyl)oxy)methyl)pyridin-1-ium tetrafluoroborate (1-Cou) 13

2.2.15 N-methyl-3-oxo-4-methanol-pyridinium (1z-OH).. 13

2.2.16 Synthesis of 4-(aldehyde)-3-hydroxy-1-methylpyridin-1-ium (1-CHO).......................... 14

2.2.17 Synthesis of 4-(carboxy)-3-hydroxy-1-methylpyridin-1-ium (1-COOH)......................... 14

2.3 Characterization data.. 15

2.4 HRMS of the synthesized compounds.. 32

3 Photorelease studies 36

3.1 General information 36

3.2 Molar Absorption Coefficient determination.. 40

3.3 Photorelease under air and inert conditions 40

3.4 Thermal stability of the compound 1z-OAc... 42

3.5 Photoproduct analysis 43

3.5.1 LC-MS studies 43

3.5.2 NMR Spectroscopic Studies 43

4 Spectroscopy studies 44

4.1 Absorption and emission profiles.. 44

4.1.1 Spectroscopy studies... 44

4.2 pH dependant spectroscopy studies.. 45

4.3 Changes in spectroscopies with photorelease 46
5 Cell studies 47
5.1 Cell sample preparation for fluorescence imaging 47
5.2 Live cell fluorescence imaging 48
6 Laser Flash Photolysis studies 50
7 DFT calculations 54
8 References 64

1 General Information

Unless otherwise noted, all the chemicals purchased from commercial sources were used without further purification. The synthesized compounds were purified either by using silica gel columns or recrystallization. Flash column chromatography was performed using silica gel Merck 60 (230–400 mesh). ^{1}H and ^{13}C NMR were obtained using AVII-600 NMR and MR-400 NMR. High-resolution mass spectra (HRMS) were obtained using Agilent QTOF 6540 with the electrospray ionization (ESI) method. UV–vis spectra were recorded on an Agilent 8453 spectrometer, and fluorescence data were recorded in HORIBA fluoromax-4 spectrophotometer. IR spectra were obtained using Bruker Tensor 37 FTIR.

Solvent preparation for spectroscopic studies:

pH 7 buffer was prepared by mixing anhydrous dibasic sodium phosphate (4.101 g) and monobasic sodium phosphate (2.533 g) in 500 mL of either D_2O or distilled water. The pH was adjusted to 7 using KOH and HCl solutions with Orion Star A215 pH/ conductivity meter.

The pH 2 buffer was prepared by dissolving monobasic potassium phosphate (H_2PO_4) 0.136 g in D_2O or distilled water and then adjusting the pH with an HCl solution.

2 Compound Preparation

2.1 Synthetic scheme

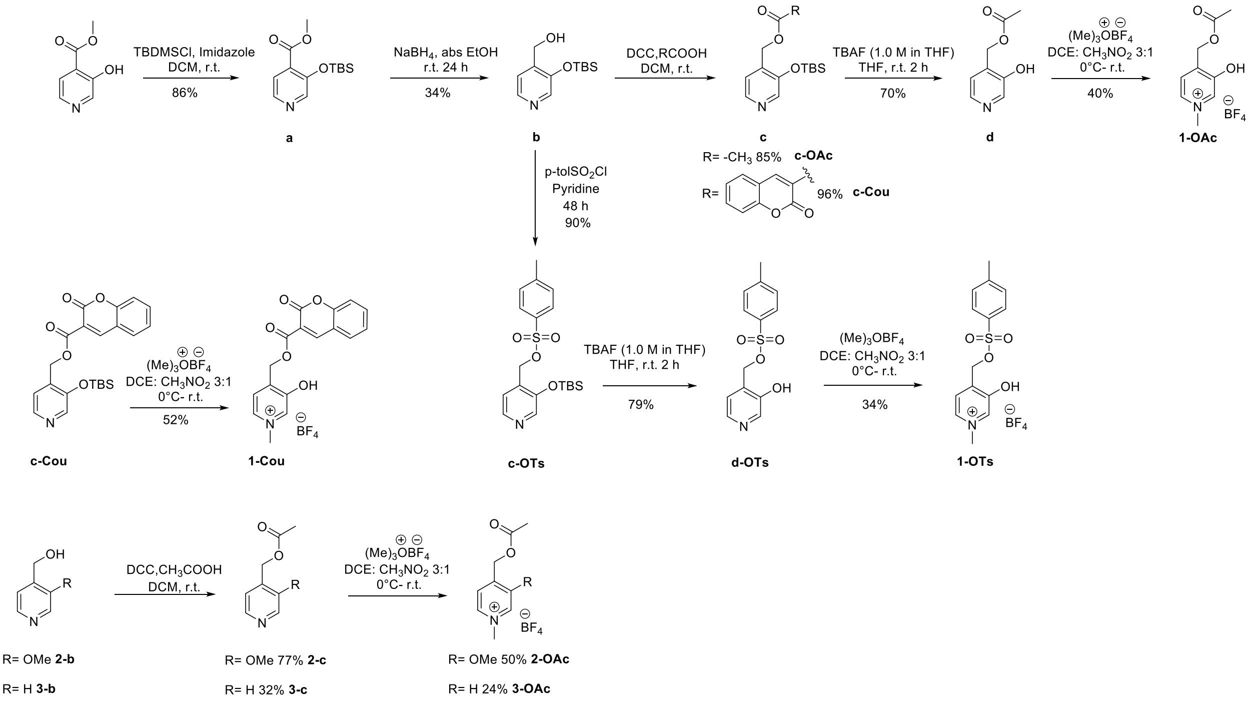

Scheme S1: Synthesis of compounds

2.2 Synthetic procedures and characterization

2.2.1 Synthesis of methyl 3-((tert-butyldimethylsilyl)oxy)isonicotinate (a)

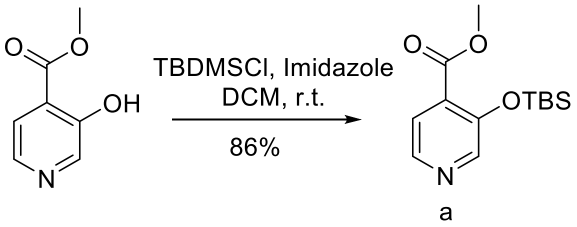

Methyl- 3-hydroxy isonicotinate (0.655 g, 4.28 mmol, 1 eq) was dissolved in 20 mL dry dichloromethane (DCM), followed by imidazole (0.350 g, 5.13 mmol, 1.2 eq). The reaction was cooled to 0˚C. Then tert-butyldimethylsilyl chloride (TBDMSCl) (0.710 g, 4.71 mmol, 1.1 eq) was added and stirred for 4 h. A large excess of H_2O was added to the reaction mixture. The solution was neutralized with saturated sodium bicarbonate ($NaHCO_3$) and extracted with ethylacetate (EtOAc) (3 × 50 mL). The combined organic layers were washed three times with brine. The organic layer was dried over anhydrous sodium sulfate (Na_2SO_4), filtered, and concentrated *in vacuo*. The resultant crude residue was purified using flash chromatography

with a gradient of 30% - 50% EtOAc in Hexane as the eluent. A white solid was obtained with an 86% (0.984 g) yield.

^{1}H NMR (600 MHz, CDCl$_3$) δ 8.26 (s, 1H), 8.20 (d, *J* = 4.9 Hz, 1H), 7.49 (d, *J* = 4.9 Hz, 1H), 3.82 (s, 3H), 0.94 (s, 9H), 0.17 (s, 6H).

^{13}C{^{1}H} NMR (101 MHz, CDCl$_3$) δ 165.53, 150.14, 143.98, 142.33, 128.90, 123.83, 123.81, 52.24, 25.50, 18.20, -4.43

HRMS (ESI/Q-TOF) m/z: $[M + H]^+$ Calcd for $C_{13}H_{21}NO_3Si$ 268.1369; Found 268.1364.

2.2.2 Synthesis of (3-((tert-butyldimethylsilyl)oxy)pyridin-4-yl)methanol (b)

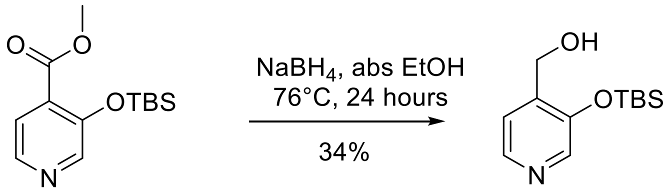

a b

To a solution of **a** (1.300 g, 4.86 mmol, 1 eq) in absolute ethanol (50 mL), $NaBH_4$ (1.854 g, 49 mmol, 10 eq) was added in one portion and stirred for 1 h at room temperature. The solution was then refluxed for 12 h and cooled to room temperature, and 50 mL of saturated ammonium chloride (NH_4Cl) was added. Ethanol was removed under a vacuum, and the precipitated solid was dissolved in a minimum amount of water and extracted three times with EtOAc. The combined organic layer was washed with brine, dried with Na_2SO_4, and concentrated under a vacuum. The resultant solid was purified via column chromatography with 50% - 80% EtOAc in hexane as the eluent. A white solid was obtained with a 34% (0.395 g) yield.

^{1}H NMR (600 MHz, CDCl$_3$) δ 8.16 (s, 1H), 8.07 (d, J = 4.9 Hz, 1H), 7.33 (d, J = 4.9 Hz, 1H), 4.88 (s, 2H), 0.96 (s, 9H), 0.15 (s, 6H).

^{13}C{^{1}H} NMR (151 MHz, CDCl$_3$) δ 152.87, 139.83, 137.82, 135.91, 121.74, 61.48, 26.21, 18.66, -5.11

HRMS (ESI/Q-TOF) m/z: $[M + H]^+$ Calcd for $C_{12}H^{21}NO_2Si$ 240.1419; Found 240.1414.

General procedure 1: attaching the leaving group

Glacial acetic acid (1.1 eq), respective alcohol (1 eq), and 4-dimethylaminopyridine (DMAP) (0.011 eq) were dissolved in DCM (50 mL) at 0˚C. To which a solution of N, N '-Dicyclohexylcarbodiimide (DCC) (1.2 eq) in DCM (10 mL) was slowly added. The reaction mixture was stirred at room temperature for 24 h and then filtered. The filtrate was washed with saturated $NaHCO_3$, and dried over anhydrous Na_2SO_4, followed by solvent removal in vacuo. The resultant solid was purified via flash chromatography.

2.2.3 Synthesis of (3-((tert-butyldimethylsilyl)oxy)pyridin-4-yl)methyl acetate (c)

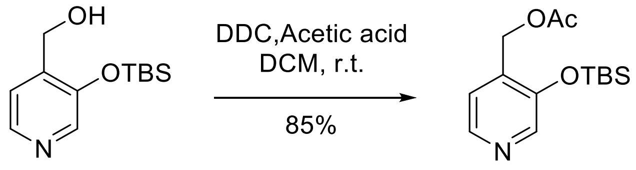

b c

Following the general procedure 1, glacial acetic acid (0.40 mL, 6.9 mmol, 1.1 eq), **b** (1.500 g, 6.27 mmol, 1 eq), DMAP (0.008 g, 0.069 mmol, 0.011 eq), DCC (1.550 g, 7.52 mmol, 1.2 eq) were reacted. The resultant solid was purified via flash chromatography with 30% - 50% EtOAc in hexanes as the eluent. A colorless liquid was obtained with an 85% (1.650 g) yield.

^{1}H NMR (600 MHz, $CDCl_3$) δ 8.39 (d, J = 4.9 Hz, 1H), 8.24 (s, 1H), 7.43 (d, J = 5.0 Hz, 1H), 4.58 (s, 2H), 2.22 (s, 3H), 0.86 (s, 9H), 0.02 (s, 6H).

^{13}C{^{1}H} NMR (151 MHz, $CDCl_3$) δ 167.97, 146.99, 143.78, 142.88, 142.10, 121.20, 58.68, 25.43, 20.16, -5.85.

HRMS (ESI/Q-TOF) m/z: $[M + H]^+$ Calcd for $C_{14}H_{23}NO_3Si$ 282.1525; Found 282.1523.

2.2.4 Synthesis of (3-methoxypyridin-4-yl)methyl acetate (2-c)

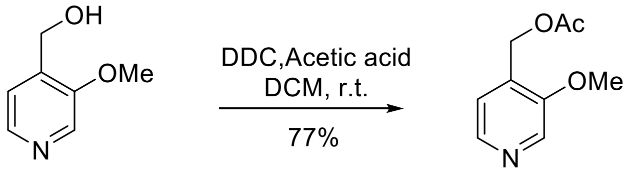

2-c

Following the general procedure 1, glacial acetic acid (0.40 mL, 6.9 mmol, 1.1 eq), 3-methoxy-4-(hydroxymethyl)pyridine (0.872 g, 6.27 mmol, 1 eq), DMAP (0.008 g, 0.069 mmol, 0.011 eq), DCC (1.550 g, 7.524 mmol, 1.2 eq) were reacted. The resultant solid was purified via column chromatography with 30% - 50% EtOAc in hexanes as the eluent. A white solid was obtained with a 77% (0.962 g) yield.

^{1}H NMR (600 MHz, MeOD) δ 8.26 (s, 1H), 8.18 (d, J = 4.8 Hz, 1H), 7.36 (d, J = 4.9 Hz, 1H), 5.16 (s, 2H), 3.97 (s, 3H), 2.14 (s, 3H).

^{13}C{^{1}H} NMR (151 MHz, MeOD) δ 172.19, 154.73, 143.01, 135.72, 133.21, 123.26, 60.96, 56.81, 20.61.

HRMS (ESI/Q-TOF) m/z: $[M + H]^+$ Calcd for $C_9H_{11}NO_3$ 182.0817; Found 182.0818.

2.2.5 Synthesis of (3-((tert-butyldimethylsilyl)oxy)pyridin-4-yl)methyl 2-oxo-2H-chromene-3-carboxylate (c-Cou)

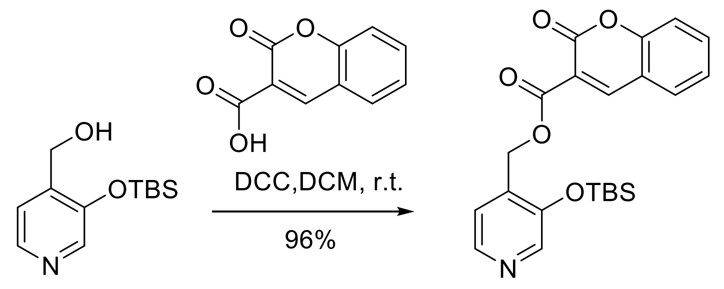

b **c-Cou**

Following the general procedure 1, coumarin-3-carboxylic acid (0.435 g, 2.3 mmol, 1.1 eq), b (0.500 g, 2.1 mmol, 1 eq), DMAP (0.003 g, 0.0231 mmol, 0.11 eq), DCC (0.520 g, 2.5 mmol, 1.2 eq) were reacted. The resultant solid was purified via column chromatography with 30% - 50% EtOAc in hexanes as the eluent. A white solid was obtained with a 96% (0.840 g) yield.

^{1}H NMR (600 MHz, $CDCl_3$) δ 8.74 (s, 1H), 8.56 – 8.53 (m, 2H), 7.74 – 7.71 (m, 1H), 7.69 (d, *J* = 7.7 Hz, 1H), 7.64 (d, *J* = 5.0 Hz, 1H), 7.44 – 7.38 (m, 2H), 4.84 (s, 2H), 0.94 (s, 9H), 0.11 (s, 6H).

^{13}C{^{1}H} NMR (151 MHz, $CDCl_3$) δ 161.24, 156.33, 155.76, 150.94, 147.24, 144.29, 142.40, 135.45, 130.06, 125.33, 122.16, 117.86, 117.17, 116.97, 59.46, 25.99, 18.46, -5.25.

HRMS (ESI/Q-TOF) m/z: $[M + H]^+$ Calcd for $C_{22}H_{25}NO_5Si$ 412.1580; Found 412.1572.

2.2.6 Synthesis of (3-((tert-butyldimethylsilyl)oxy)pyridin-4-yl)methyl 4-methylbenzenesulfonate (c-OTs)

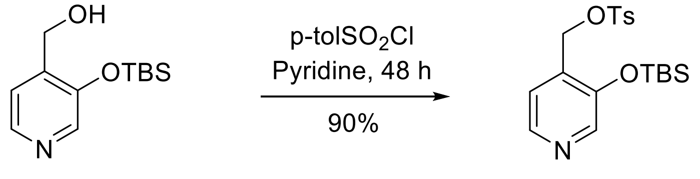

b **c-OTs**

b (0.50 g, 2.1 mmol, 1 eq) and p-toluenesulfonylchloride (0.498 g, 2.6 mmol, 1.2 eq) were dissolved in pyridine (10 mL) and stirred for 48 h. Pyridine was then co-evaporated with heptane, followed by suspending the residue in 50% EtOAc in hexane (10 mL) and filtering. The filtrate was concentrated under a vacuum. The resulting liquid was purified by flash chromatography with 20%-50% EtOAc in hexane gradient as eluent to give a clear liquid with 90% (1.000 g) yield.

^{1}H NMR (400 MHz, $CDCl_3$) δ 8.47 (d, J = 4.9 Hz, 1H), 8.04 (s, 1H), 7.73 (d, J = 8.4 Hz, 2H), 7.51 (dd, J = 5.0, 0.7 Hz, 1H), 7.35 (dt, J = 8.0, 0.8 Hz, 2H), 4.61 (d, J = 1.1 Hz, 2H), 2.46 (s, 3H), 0.92 (s, 9H), 0.05 (s, 6H).

^{13}C{^{1}H} NMR (101 MHz, $CDCl_3$) δ 148.53, 146.30, 144.77, 143.37, 143.22, 132.27, 130.30, 128.58, 122.09, 58.85, 25.93, 21.87, 18.40, -5.40.

HRMS (ESI/Q-TOF) m/z: $[M + H]^+$ Calcd for $C_{19}H_{27}NO_4SSi$ 394.1508; Found 394.1512.

2.2.7 Synthesis of pyridin-4-ylmethyl acetate (3-c)

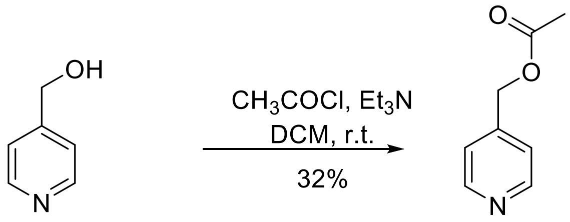

3-c

Acetyl chloride (0.54 mL, 7.53 mmol, 1.4 eq) dissolved in 2.3 mL DCM was added dropwise to a solution of 4-(hydroxymethyl)pyridine (0.583 g, 5.35 mmol, 1 eq), triethylamine (1.3 mL, 9.3 mmol, 1.7 eq), in DCM (6 mL). The solution was stirred for 24 h and was concentrated under a vacuum. The resultant liquid was purified via flash chromatography with 50%-80% EtOAc in hexane as the eluent. A colorless liquid was obtained with a 32% (0.256 g) yield.

^{1}H NMR (600 MHz, $CDCl_3$) δ 8.51 (s, 2H), 7.16 (s, 2H), 5.03 (s, 2H), 2.06 (s, 3H).

^{13}C{^{1}H} NMR (101 MHz, MeOD) δ 171.76, 158.20, 146.56, 126.26, 64.31, 20.39.

HRMS (ESI/Q-TOF) m/z: $[M + H]^+$ Calcd for $C_8H_9NO_2$ 152.0711; Found 152.0707

General procedure 2: TBS deprotection

Tetrabutylammonium fluoride (TBAF) (1.0 M in THF, 1.2 eq) was slowly added to a stirred solution of the corresponding leaving group attached compound (1 eq) in THF (4 mL). After all the starting material was consumed, the reaction was quenched with water and was extracted with ethyl acetate (3x20 mL). Organic layers were combined, washed with brine, and dried

over Na_2SO_4. The crude solid residue obtained after removing the solvent was purified via flash chromatography.

2.2.8 Synthesis of (3-hydroxypyridin-4-yl)methyl acetate (d)

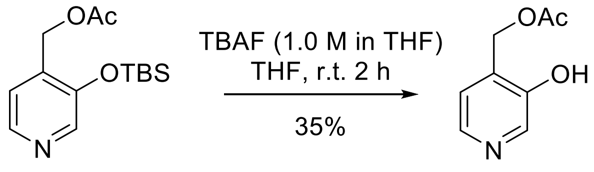

c d

Following the general procedure 2, TBAF (1.0 M in THF, 1.2 mL, 1.20 mmol, 1.2 eq), **c** (0.169 g, 0.60 mmol, 1 eq) were reacted. The crude solid residue obtained after removing the solvent was purified with 80% ethyl acetate in hexane followed by 2% methanol in chloroform. A white powder was obtained with a 35% (0.035 g) yield.

^{1}H NMR (600 MHz, $CDCl_3$) δ 8.24 (s, 1H), 8.09 (d, J = 4.9 Hz, 1H), 7.31 (d, J = 5.0 Hz, 1H), 5.23 (s, 2H), 2.17 (s, 3H).

$^{13}C\{^1H\}$ NMR (151 MHz, $CDCl_3$) δ 171.09, 152.97, 139.28, 136.07, 133.01, 123.59, 60.52, 20.80.

HRMS (ESI/Q-TOF) m/z: $[M + H]^+$ Calcd for $C_8H_9NO_3$ 168.0661; Found 168.0660.

2.2.9 Synthesis of (3-hydroxypyridin-4-yl)methyl 4-methylbenzenesulfonate (d-OTs)

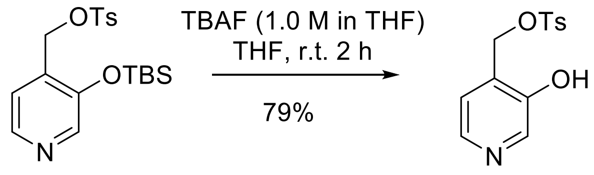

c-OTs **d-OTs**

Following the general procedure 2, TBAF (1.0 M in THF, 3.0 mL, 3.0 mmol, 1.2 eq), **c-OTs** (1.00 g, 2.5 mmol, 1 eq) were reacted in THF (15mL). The crude solid residue obtained after removing the solvent was purified with 85% ethyl acetate in hexane. A white powder was obtained with a 79% (0.550 g) yield.

^{1}H NMR (400 MHz, $CDCl_3$) δ 8.48 (d, J = 5.0 Hz, 1H), 7.96 (s, 1H), 7.75 (d, J = 8.4 Hz, 2H), 7.56 – 7.52 (m, 1H), 7.40 – 7.35 (m, 2H), 4.70 (s, 2H), 2.48 (s, 3H).

$^{13}C\{^1H\}$ NMR (101 MHz, $CDCl_3$) δ 148.69, 146.57, 144.22, 143.79, 143.64, 131.80, 130.38, 128.68, 123.48, 58.66, 21.93.

HRMS (ESI/Q-TOF) m/z: $[M + H]^+$ Calcd for $C_{13}H_{13}NO_4S$ 280.0643; Found 280.0639.

General procedure 3: methylation

To an ice-cold solution of the corresponding d compound (1 eq) in DCE (6 mL), a solution of trimethyloxonium tetrafluoroborate (1.1eq) in nitromethane (2 mL) was slowly added. The reaction was stirred at room temperature for 12 h unless otherwise noted. The solvent was removed in a vacuum, and the resultant oil was washed with the corresponding solvent mixture until the product crashed out.

2.2.10 Synthesis of 4-(acetoxymethyl)-3-hydroxy-1-methylpyridin-1-ium (1-OAc)

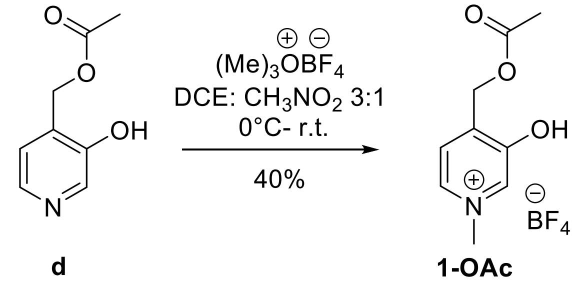

Following the general procedure 3, compound **d** (0.390 g, 2.33 mmol, 1 eq) and trimethyloxonium tetrafluoroborate (0.380 g, 2.56 mmol, 1.1eq) were reacted. The resultant brown oil was washed with ethyl acetate and hexane mixture to yield 40% (0.251 g) white powder.

^{1}H NMR (400 MHz, D_2O) δ 8.25 (s, 1H), 8.23 (s, 1H), 7.83 (d, *J* = 6.1 Hz, 1H), 5.31 (s, 2H), 4.25 (s, 3H), 2.19 (s, 3H).

^{13}C{^{1}H} NMR (101 MHz, D_2O) δ 174.73, 154.86, 143.07, 137.91, 132.91, 126.58, 61.55, 21.35.

HRMS (ESI/Q-TOF) m/z: $[M]^+$ Calcd for $C_9H_{12}NO_3$ 182.0817; Found 182.0817.

2.2.11 Synthesis of 4-(acetoxymethyl)-3-methoxy-1-methylpyridin-1-ium (2-OAc)

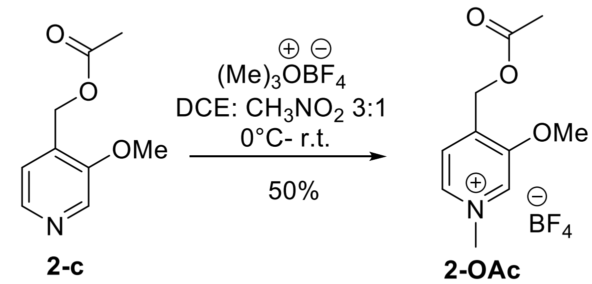

Following the general procedure 3, **2-c** (0.4220 g, 2.33 mmol, 1 eq), trimethyloxonium tetrafluoroborate (0.3790, 2.563 mmol, 1.1eq) was reacted. The resultant brown oil was recrystallized with DCM to give a white powder at 50% (0.330 g) yield.

^{1}H NMR (600 MHz, CDCl3) δ 8.07 (dd, J = 5.3, 0.8 Hz, 1H), 6.76 – 6.73 (m, 1H), 6.64 (dd, J = 1.5, 0.8 Hz, 1H), 5.00 (s, 2H), 3.87 (s, 3H), 2.08 (s, 3H).

^{13}C{^{1}H} NMR (151 MHz, CDCl3) δ 170.41, 164.50, 147.69, 147.05, 115.05, 108.79, 64.12, 53.41, 20.71.

HRMS (ESI/Q-TOF) m/z: $[M]^+$ Calcd for $C_{10}H_{14}NO_3$ 196.0974; Found 196.0964.

2.2.12 Synthesis of 3-hydroxy-1-methyl-4-((tosyloxy)methyl)pyridin-1-ium tetrafluoroborate (1-OTs)

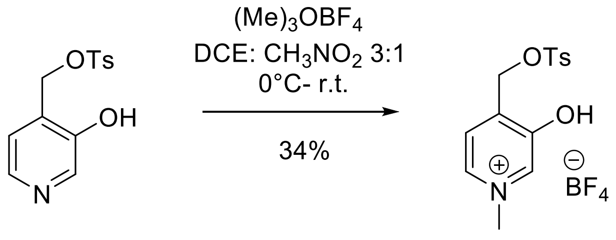

d-OTs **1-OTs**

Following the general procedure 3, d-OTs (0.55 g, 1.97 mmol, 1 eq), trimethyloxonium tetrafluoroborate (0.360, 2.167 mmol, 1.1eq) were reacted. The resultant brown oil was sonicated with ethyl acetate to give a white powder in 34% (0.255 g) yield.

^{1}H NMR (400 MHz, D_2O) δ 8.81 (d, *J* = 1.4 Hz, 1H), 8.78 (dd, *J* = 6.3, 1.4 Hz, 1H), 8.15 (d, *J* = 6.3 Hz, 1H), 7.87 – 7.83 (m, 2H), 7.57 – 7.53 (m, 2H), 4.48 (s, 2H), 4.39 (s, 3H), 2.49 (s, 3H).

^{13}C{^{1}H} NMR (101 MHz, D_2O) δ 155.31, 148.86, 144.42, 144.17, 139.58, 130.76, 129.14, 128.49, 125.94, 57.34, 48.10, 20.97.

HRMS (ESI/Q-TOF) m/z: $[M]^+$ Calcd for $C_{14}H_{16}NO_4S$ 294.0800; Found 294.0801.

2.2.13 Synthesis of 4-(acetoxymethyl)-1-methylpyridin-1-ium tetrafluoroborate (3-OAc)

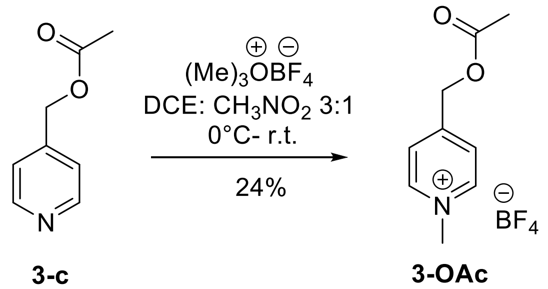

3-c **3-OAc**

Following the general procedure 3, **3-c** (0.256 g, 1.7 mmol, 1 eq), trimethyloxonium tetrafluoroborate (0.262 g, 1.32 mmol, 1.1eq) were reacted. The resultant brown oil was sonicated with ethyl acetate to give a white powder in 24% (0.103 g) yield.

^{1}H NMR (600 MHz, D_2O) δ 8.69 (d, *J* = 6.3 Hz, 2H), 7.95 (d, *J* = 6.3 Hz, 2H), 5.38 (s, 3H), 4.32 (s, 4H), 2.19 (s, 3H).

^{13}C{^{1}H} NMR (151 MHz, D_2O) δ 173.37, 155.87, 144.93, 125.11, 63.73, 47.71, 20.07.

HRMS (ESI/Q-TOF) m/z: $[M]^+$ Calcd for $C_9H_{12}NO_2$ 166.0868; Found 166.0866.

2.2.14 Synthesis of 3-hydroxy-1-methyl-4-(((2-oxo-2H-chromene-3-carbonyl)oxy)methyl)pyridin-1-ium tetrafluoroborate (1-Cou)

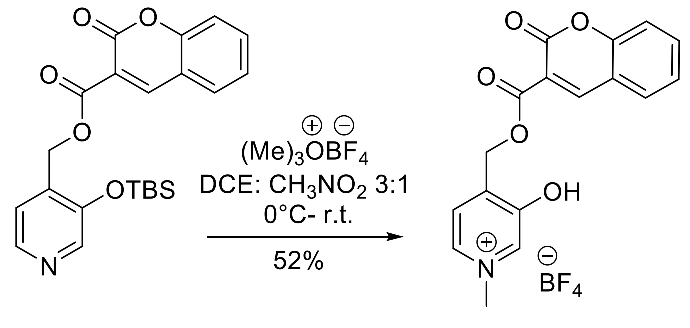

c-Cou **1-Cou**

To an ice-cold solution of c-Cou (0.200 g, 0.486 mmol, 1 eq) in DCE (6 mL), a solution of trimethyloxonium tetrafluoroborate (0.180 g, 1.215 mmol, 2.5 eq) in nitromethane (2 mL) was slowly added. The reaction was stirred at room temperature for 30 minutes, and the formed solid was filtered and washed with DCM to give a white powder in 52% (0.101 g).

^{1}H NMR (400 MHz, D_2O) δ 10.12 (s, 1H), 9.66 – 9.55 (m, 2H), 9.29 (d, *J* = 6.1 Hz, 1H), 9.07 (d, *J* = 8.3 Hz, 1H), 9.03 – 8.98 (m, 1H), 8.69 – 8.60 (m, 2H), 6.74 (s, 2H), 5.55 (s, 3H)

^{13}C{^{1}H} NMR (101 MHz, $(CD_3)_2CO$) δ 339.54, 334.56, 331.55, 330.83, 327.95, 318.22, 313.26, 312.35, 308.16, 307.26, 302.33, 301.99, 294.49, 293.11, 292.87, 237.69, 224.57.

HRMS/ESI+ For compound **1-Cou**-BF_4 $[M]^+$, 312.0871 was calculated, and 312.0879 was found.

HRMS (ESI/Q-TOF) m/z: $[M]^+$ Calcd for $C_{17}H_{14}NO_5$ 312.0871; Found 312.0879.

2.2.15 N-methyl-3-oxo-4-methanol-pyridinium (1z-OH)

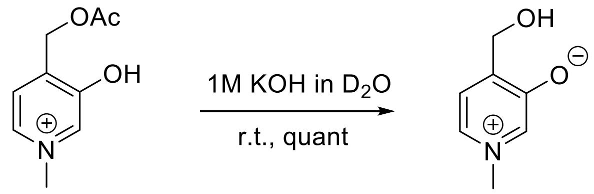

1-OAc **1z-OH**

Despite the efforts, pure compound 1z-OH couldn't be synthetically isolated; therefore, it was obtained by complete hydrolysis of compound **1-OAc**. Compound **1-OAc** was dissolved in 1M KOH (excess) and stirred until complete hydrolysis followed by NMR (typically within 5 minutes).

1H NMR (600 MHz, $CDCl_3$) δ 8.07 (dd, J = 5.3, 0.8 Hz, 1H), 6.76 – 6.73 (m, 1H), 6.64 (dd, J = 1.5, 0.8 Hz, 1H), 5.00 (s, 2H), 3.87 (s, 3H), 2.08 (s, 3H).

13C{^{1}H} NMR (151 MHz, $CDCl_3$) δ 170.41, 164.50, 147.69, 147.05, 115.05, 108.79, 64.12, 53.41, 20.71.

HRMS (ESI/Q-TOF) m/z: $[M+H]^+$ Calcd for $C_7H_{10}NO_2$ 140.0712; Found 140.0711.

2.2.16 Synthesis of 4-(aldehyde)-3-hydroxy-1-methylpyridin-1-ium (1-CHO)

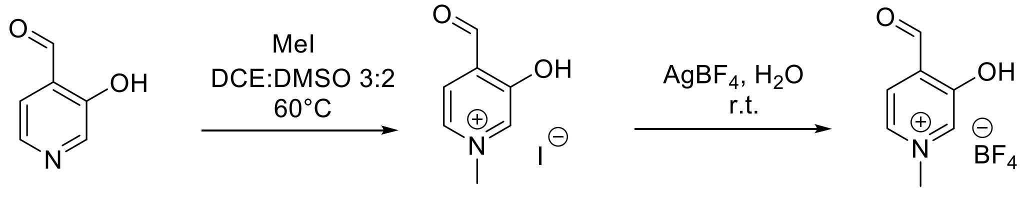

h   **1-CHO**

To a round bottom flask charged with 3-hydroxynicotialdehyde (0.500 g, 4 mmol, 1 eq) was added DCE (3.0 mL) and DMSO (2.0 mL) and stirred until all the solid was dissolved. MeI was added to the solution, heated at 60˚C for 10 minutes, and let it come to room temperature. A bright orange solid crashed out from the solution. The crashedout solid was filtered to get the compound **h**, which was then dissolved in DI water and stirred at r.t for 5 hours after excess $AgBF_4$ was added. Resultanat white solid was filtered. To the filtrate sat.NaCl (2 mL)was added. The formed white solid was filtered and the filtrate was concentrated under a vacuum to give a white powder in 75% (0.675 g). The product was used without further purification.

^{1}H NMR (600 MHz, MeOD) δ 8.33 (dd, *J* = 6.1, 1.5 Hz, 1H), 8.27 (d, *J* = 1.5 Hz, 1H), 7.98 (d, *J* = 6.1 Hz, 1H), 5.87 (s, 1H), 4.32 (s, 3H).

^{13}C{^{1}H} NMR (151 MHz, MeOD) δ 155.55, 145.54, 138.06, 134.16, 126.05, 125.83, 92.64.

HRMS (ESI/Q-TOF) m/z: $[M]^+$ Calcd for $C_7H_8NO_2$ 138.0555; Found 138.0553.

2.2.17 Synthesis of 4-(carboxy)-3-hydroxy-1-methylpyridin-1-ium (1-COOH)

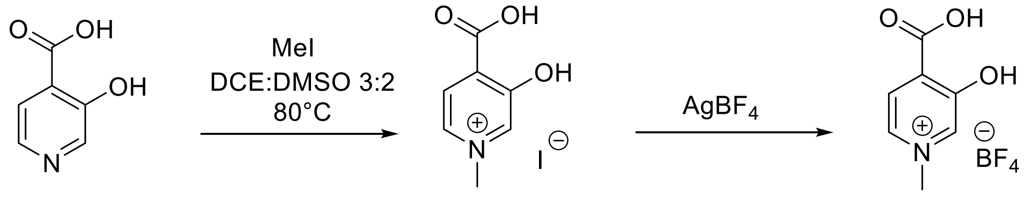

j   **1-COOH**

A similar procedure was followed as in synthesizing compound 1-CHO.

^{1}H NMR (600 MHz, D_2O) δ 8.26 (s, 1H), 8.08 (d, *J* = 6.0 Hz, 1H), 7.94 (d, *J* = 6.0 Hz, 1H), 4.30 (s, 3H).

^{13}C{^{1}H} NMR (151 MHz, D_2O) δ 170.12, 158.94, 136.02, 131.92, 129.62, 127.98, 27.42.

HRMS (ESI/Q-TOF) m/z: $[M]^+$ Calcd for $C_7H_8NO_3$ 154.0504; Found 154.0506.

2.3 Characterization data

^{1}H NMR (600 MHz, $CDCl_3$) of a

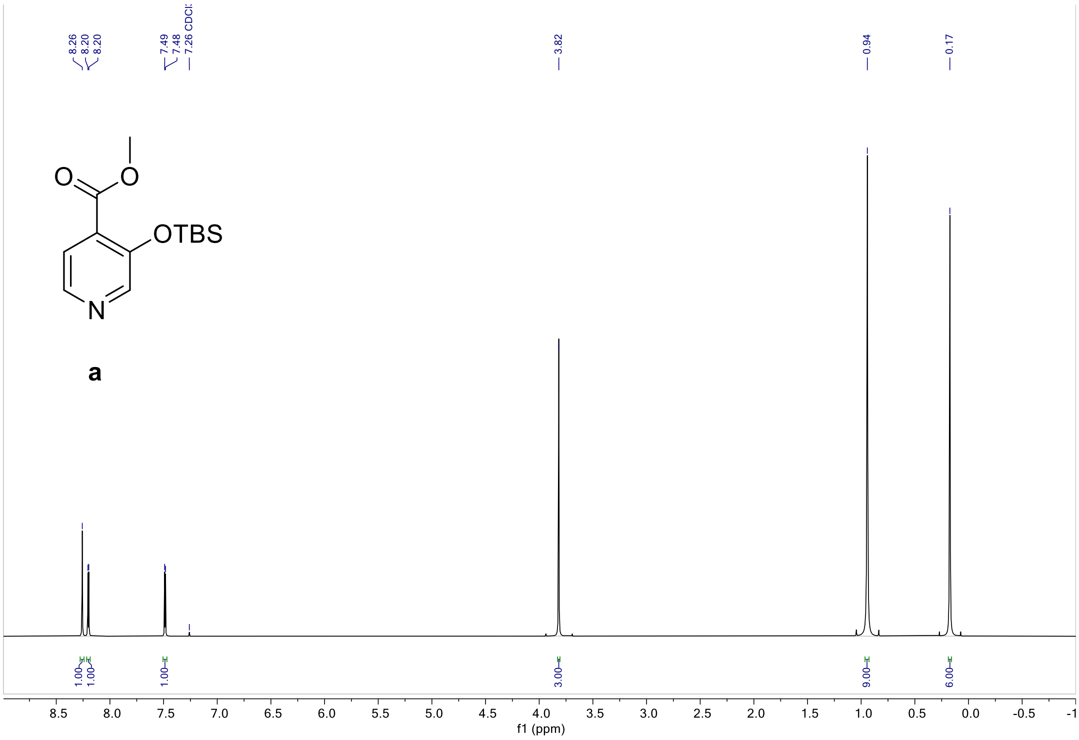

$^{13}C\{^{1}H\}$ NMR (101 MHz, $CDCl_3$) of a

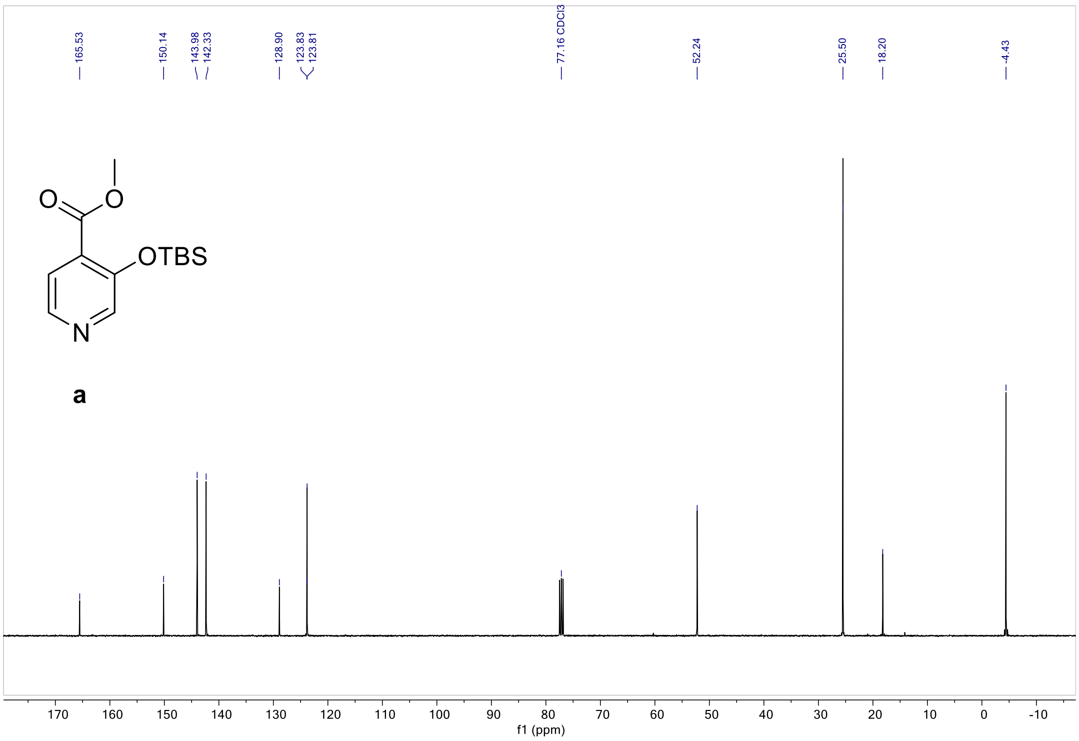

^{1}H NMR (600 MHz, CDCl₃) of b

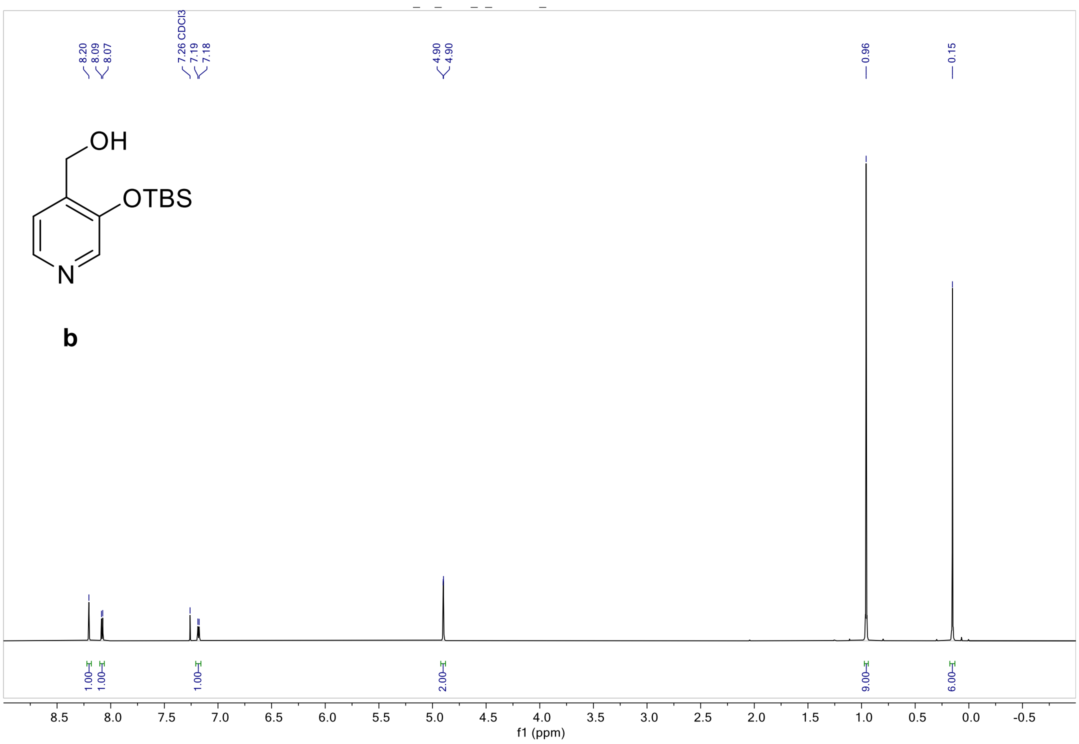

^{13}C NMR (151 MHz, CDCl₃) of b

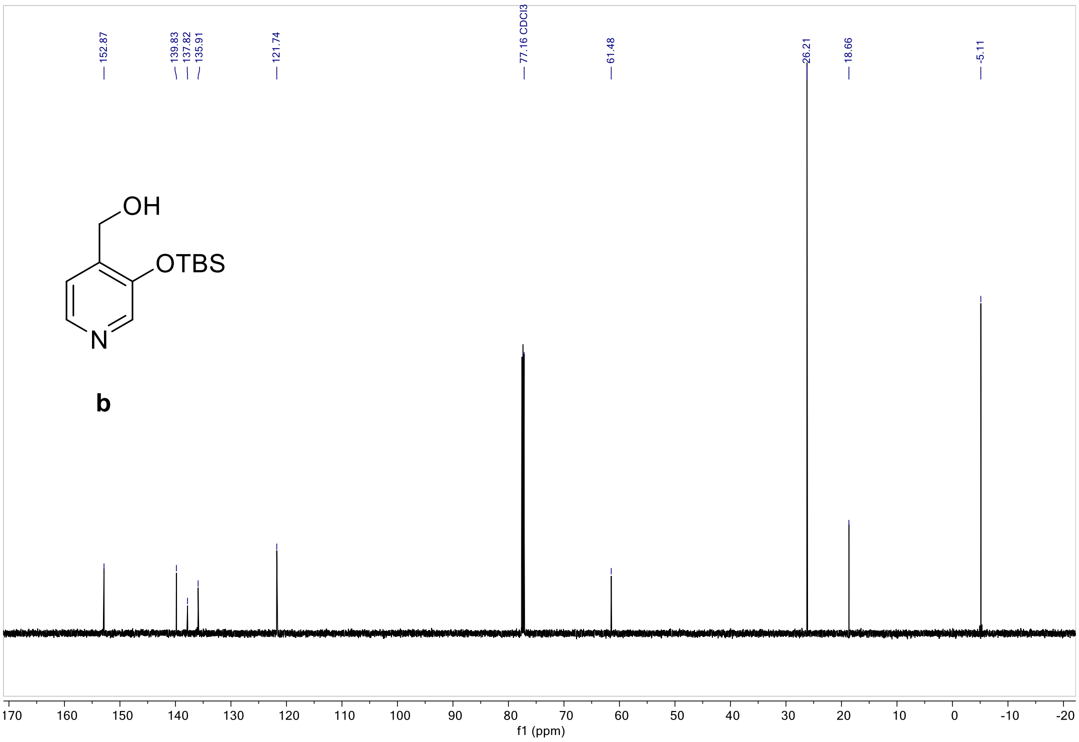

^{1}H NMR (600 MHz, $CDCl_3$) of c

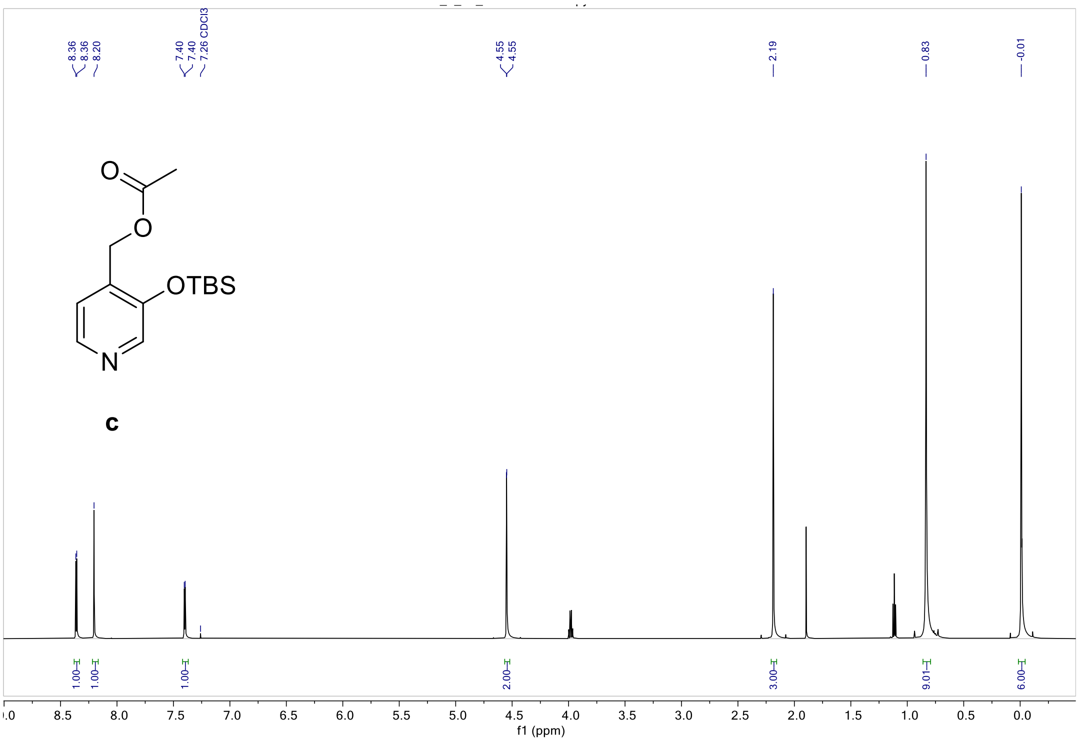

^{13}C NMR (151 MHz, $CDCl_3$) δ of c

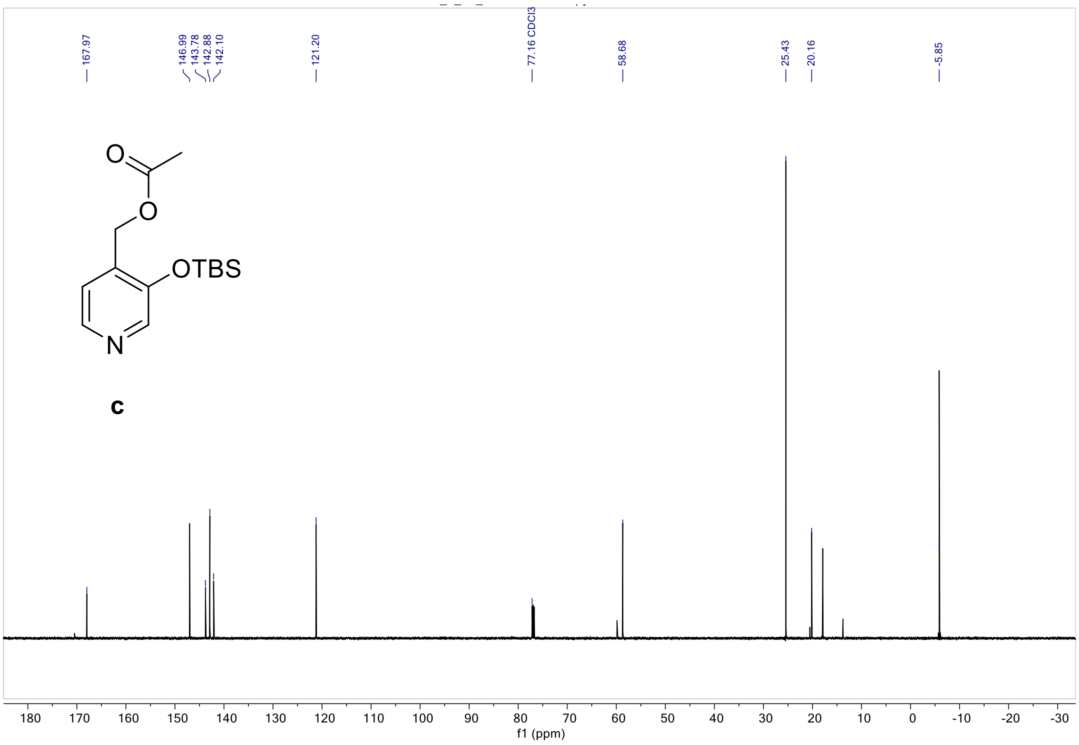

^{1}H NMR (400 MHz, $CDCl_3$) of c-OTs

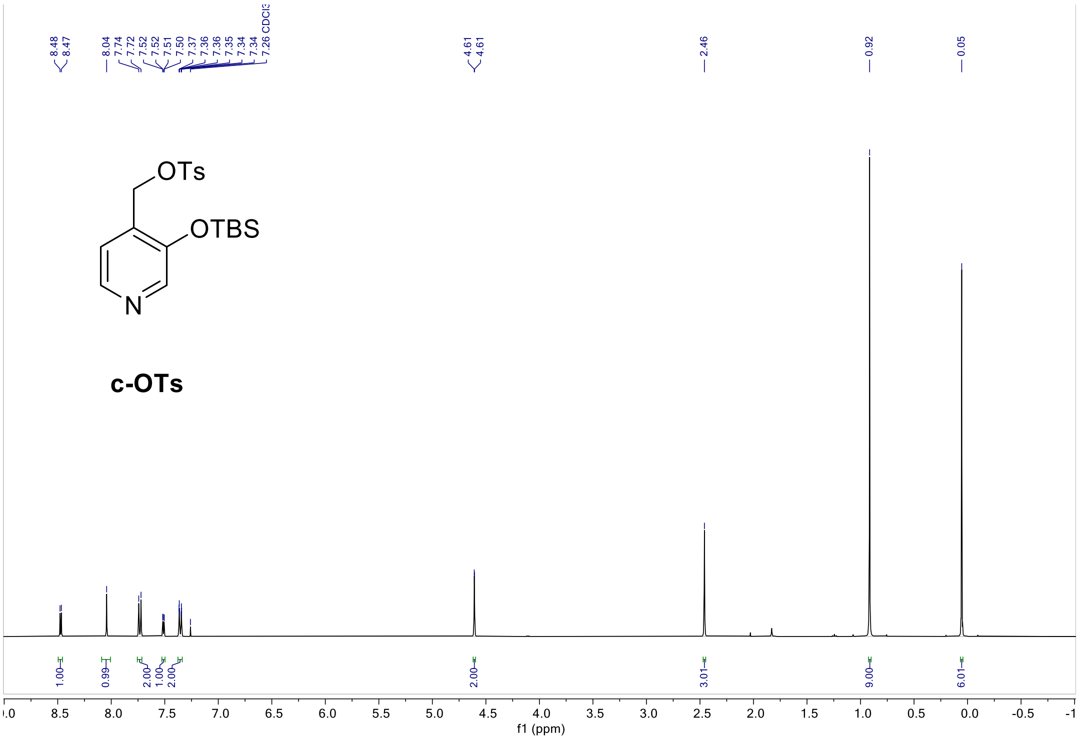

^{13}C NMR (101 MHz, $CDCl_3$) of c-OTs

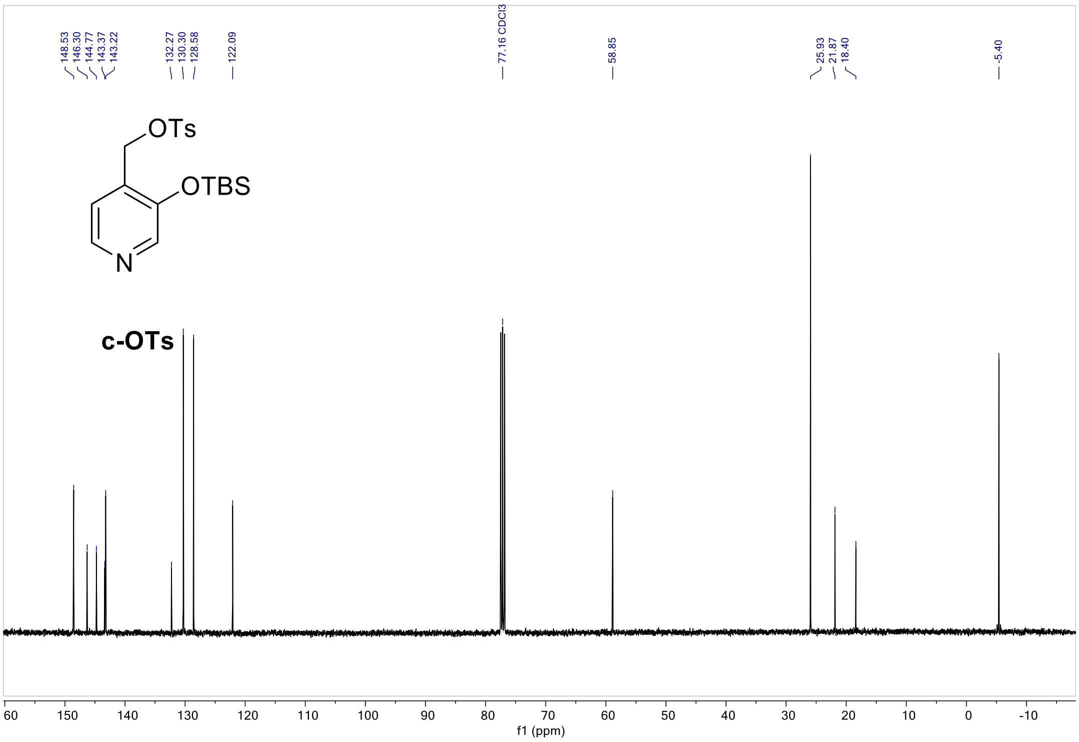

^{1}H NMR (600 MHz, $CDCl_3$) of c-Cou

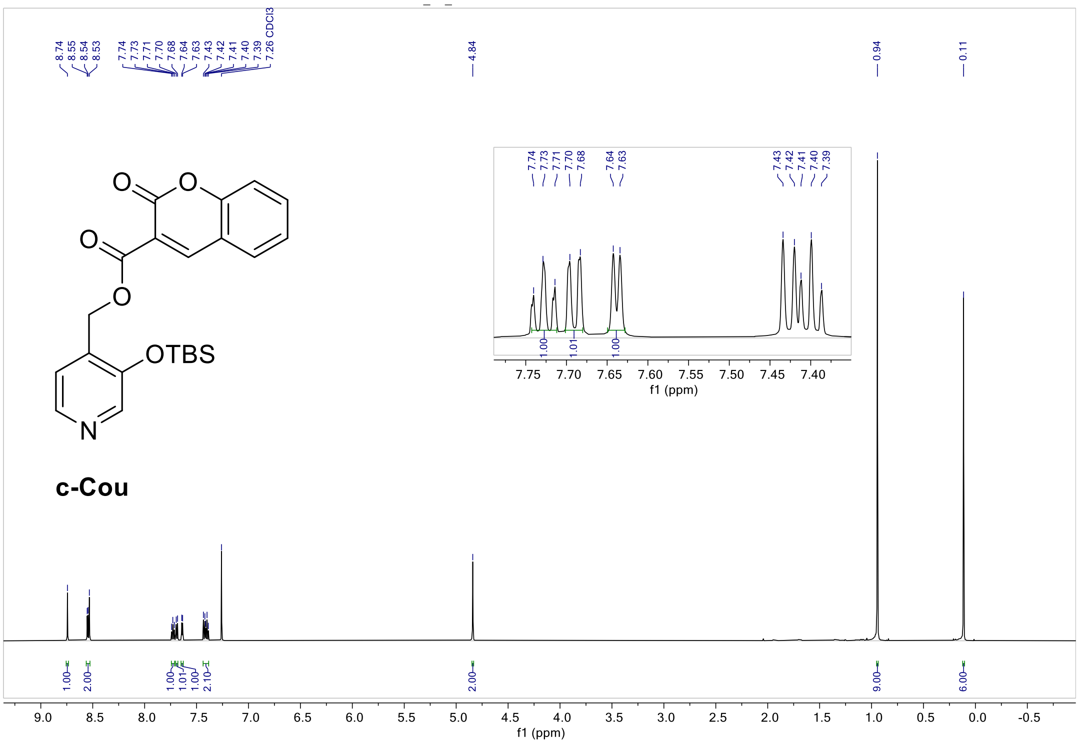

^{13}C NMR (151 MHz, $CDCl_3$) of c-Cou

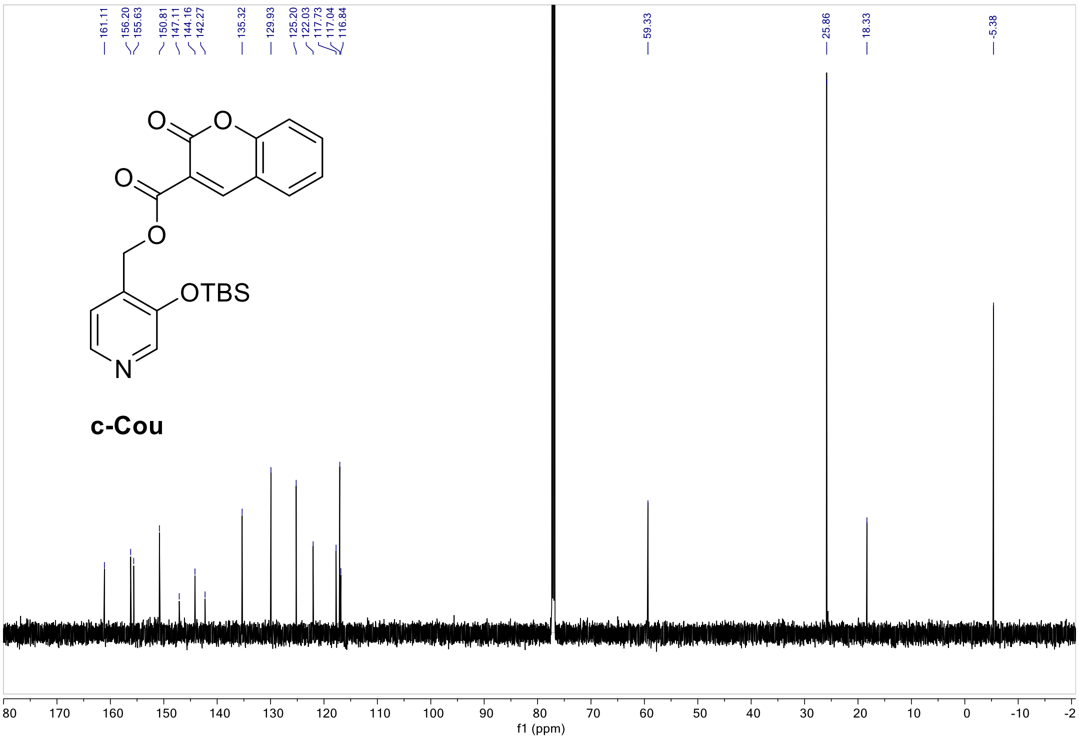

^{1}H NMR (600 MHz, $CDCl_3$) of d

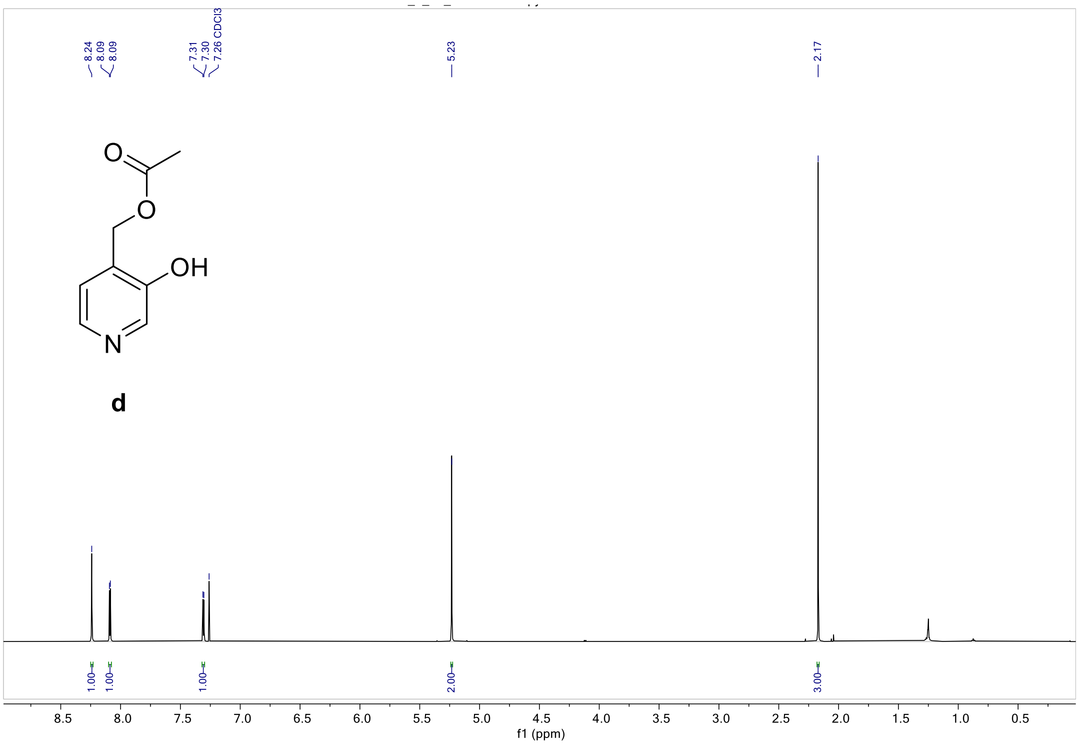

^{13}C NMR (151 MHz, $CDCl_3$) of d

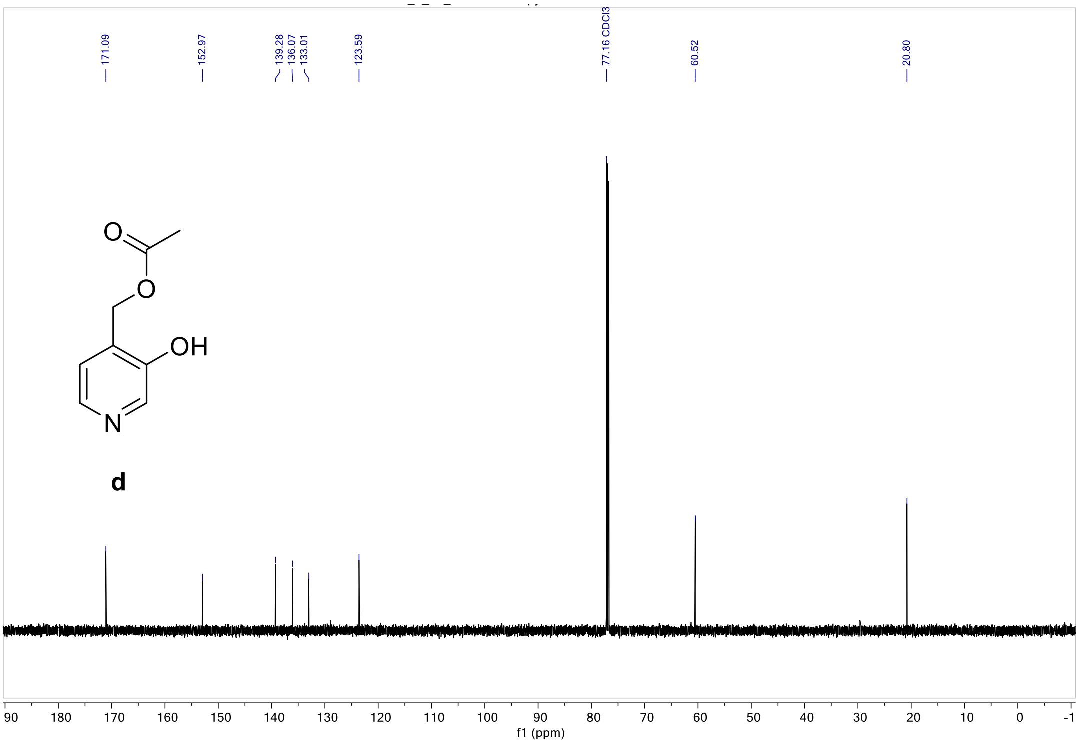

^{1}H NMR (400 MHz, $CDCl_3$) of d-OTs

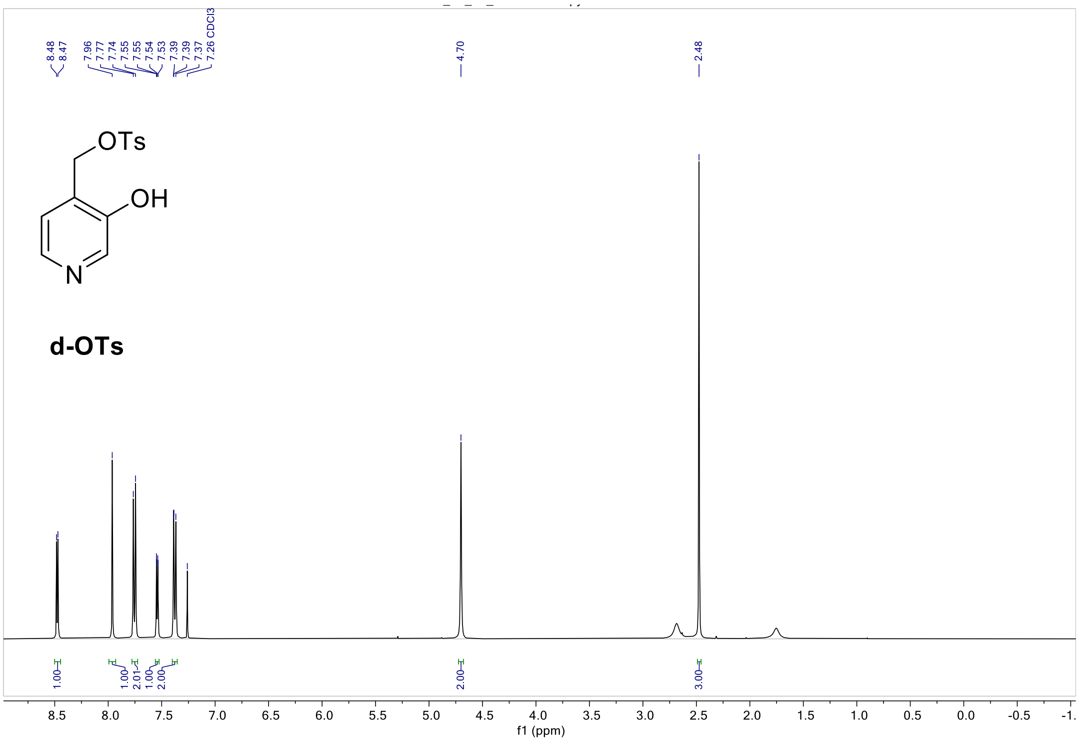

^{13}C NMR (101 MHz, $CDCl_3$) of d-OTs

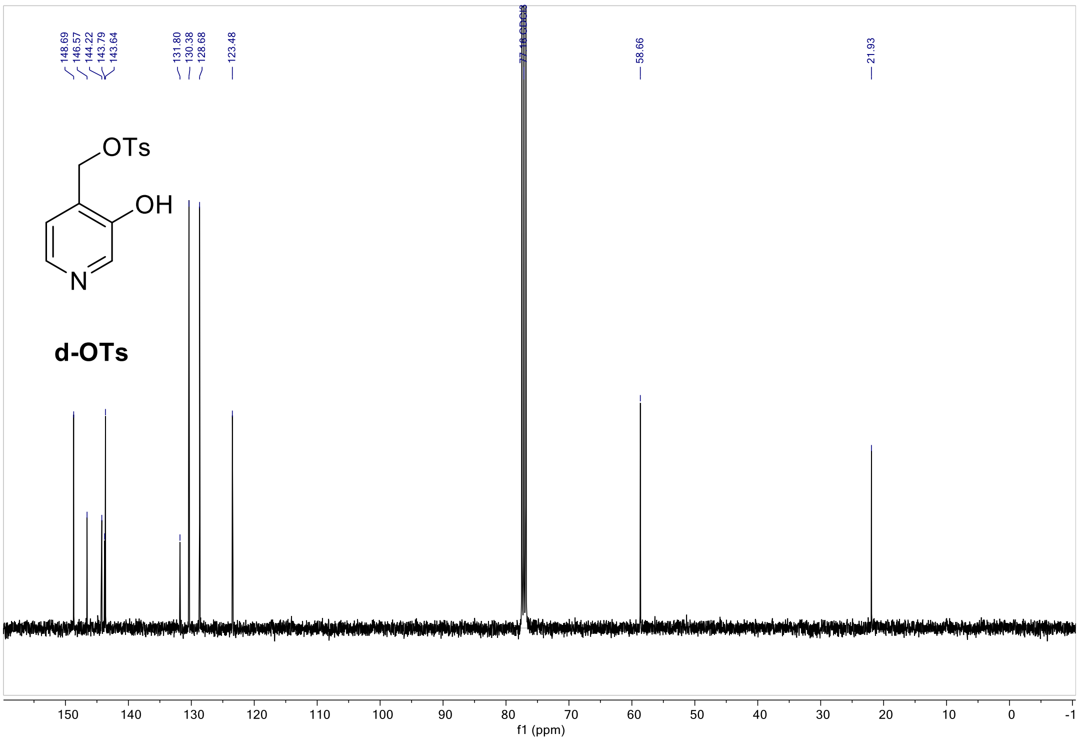

^{1}H NMR (600 MHz, MeOD) of 2-c

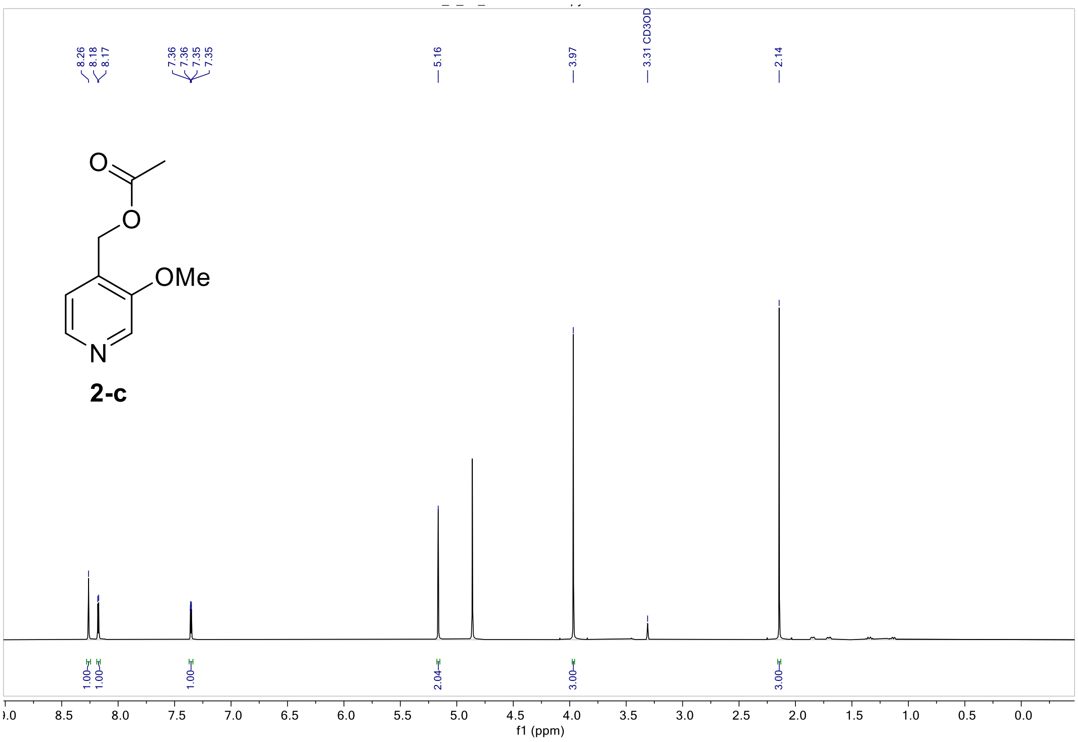

^{13}C NMR (151 MHz, MeOD) of 2-c

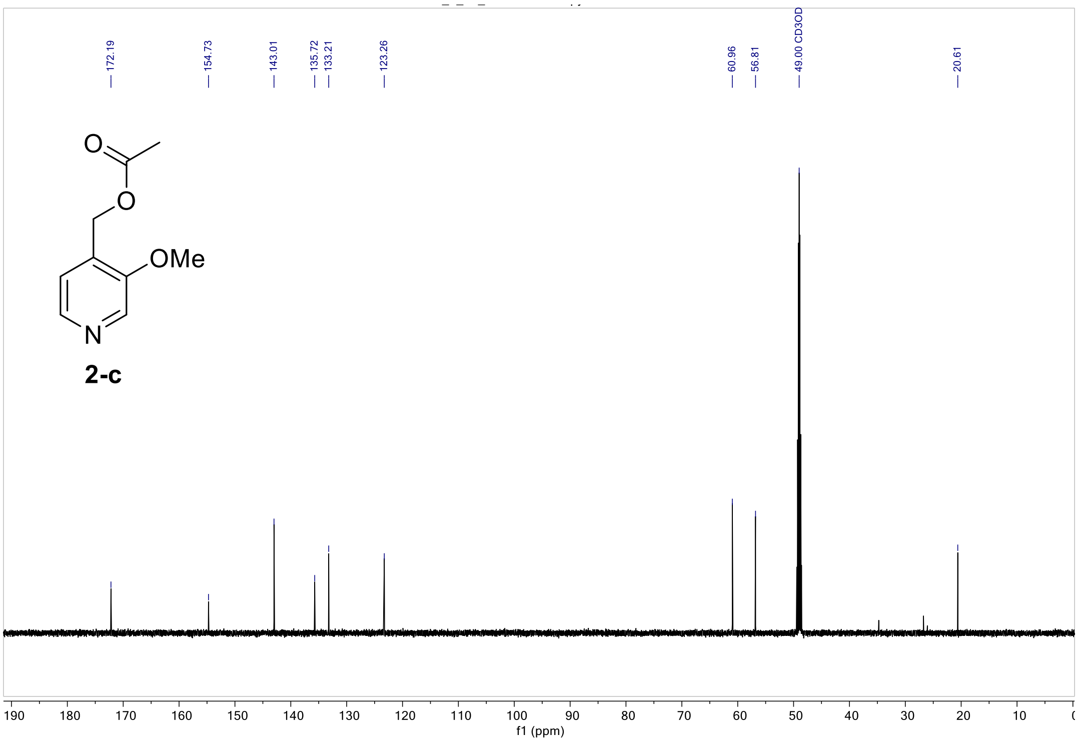

^{1}H NMR (600 MHz, $CDCl_3$) of 3-c

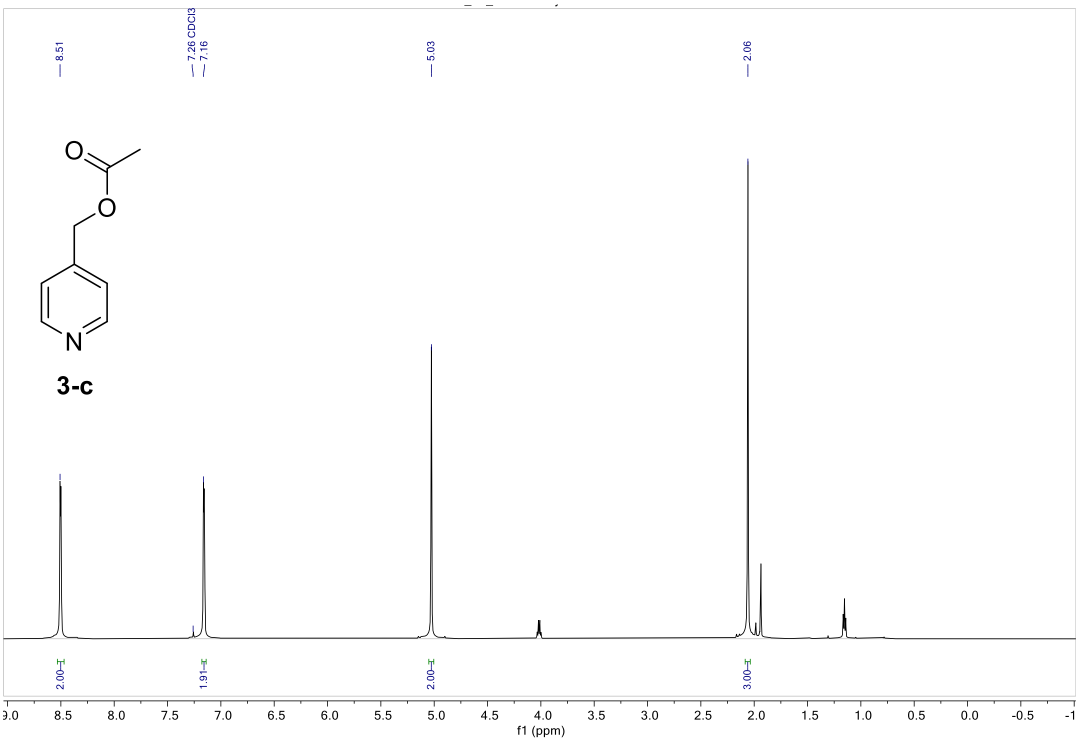

^{13}C NMR (101 MHz, MeOD) of 3-c

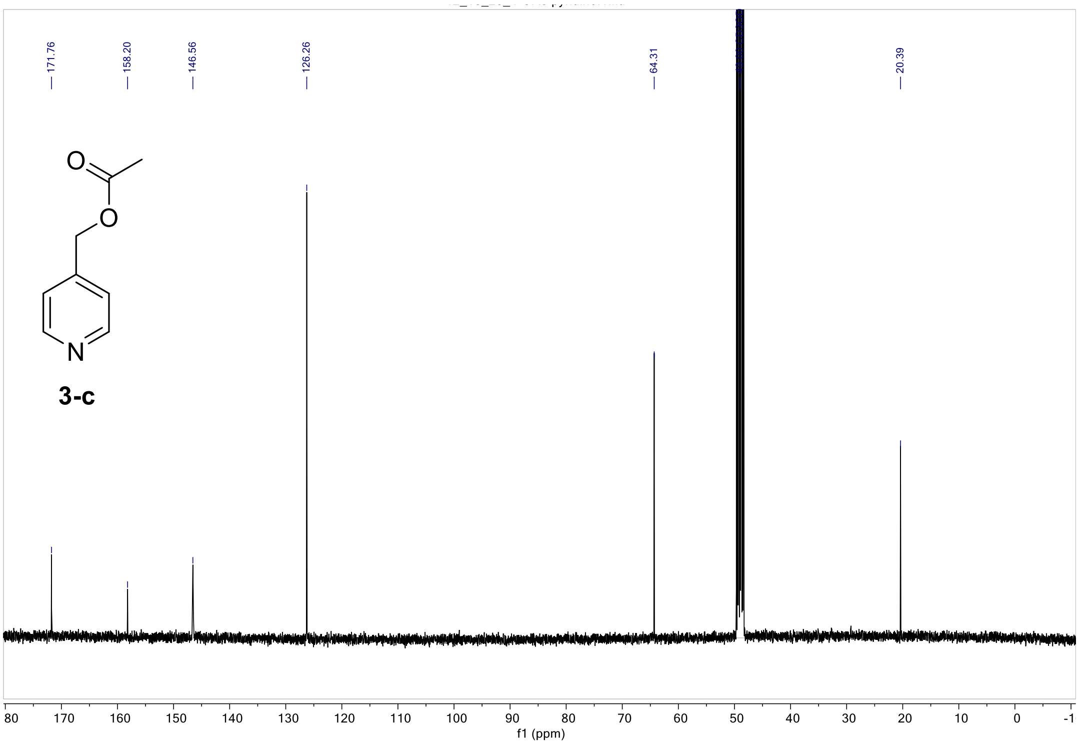

^{1}H NMR (400 MHz, D_2O) of 1-OAc

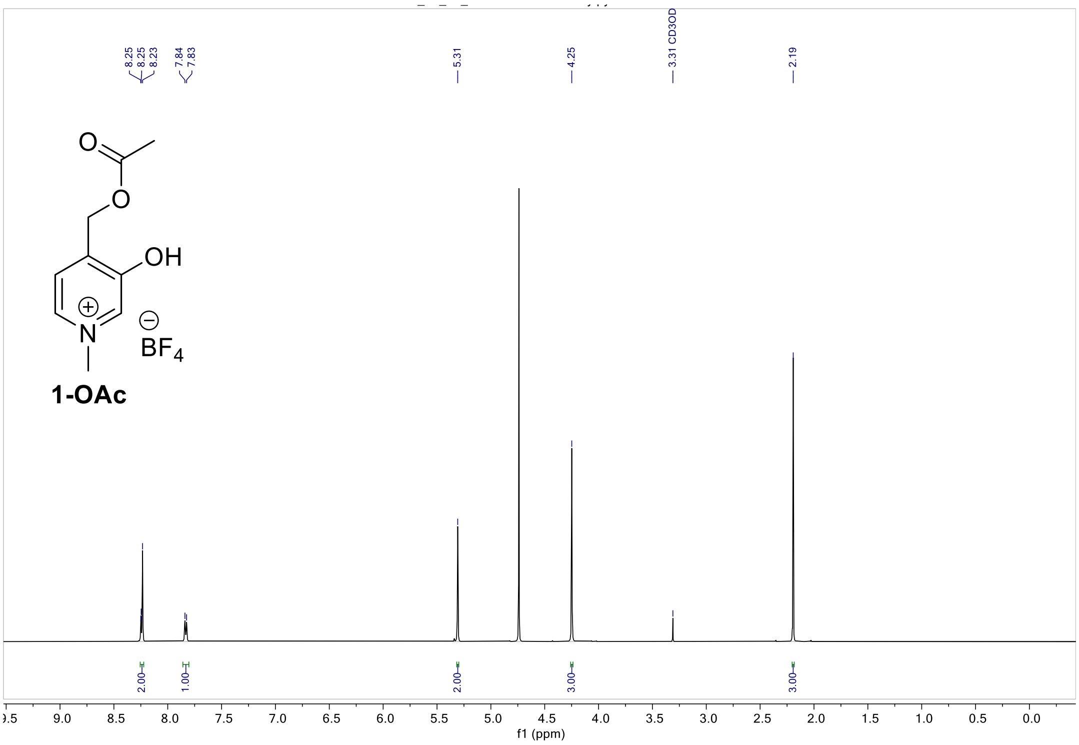

^{13}C NMR (101 MHz, D_2O) of 1-OAc

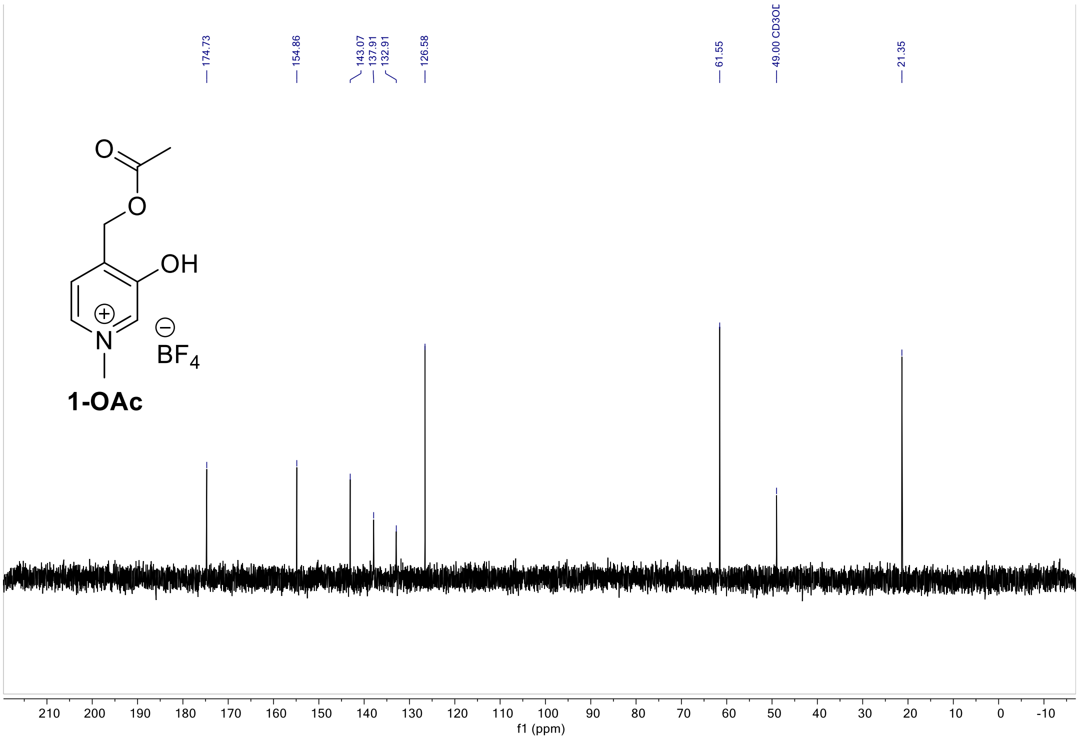

^{1}H NMR (400 MHz, D_2O) of 1-OTs

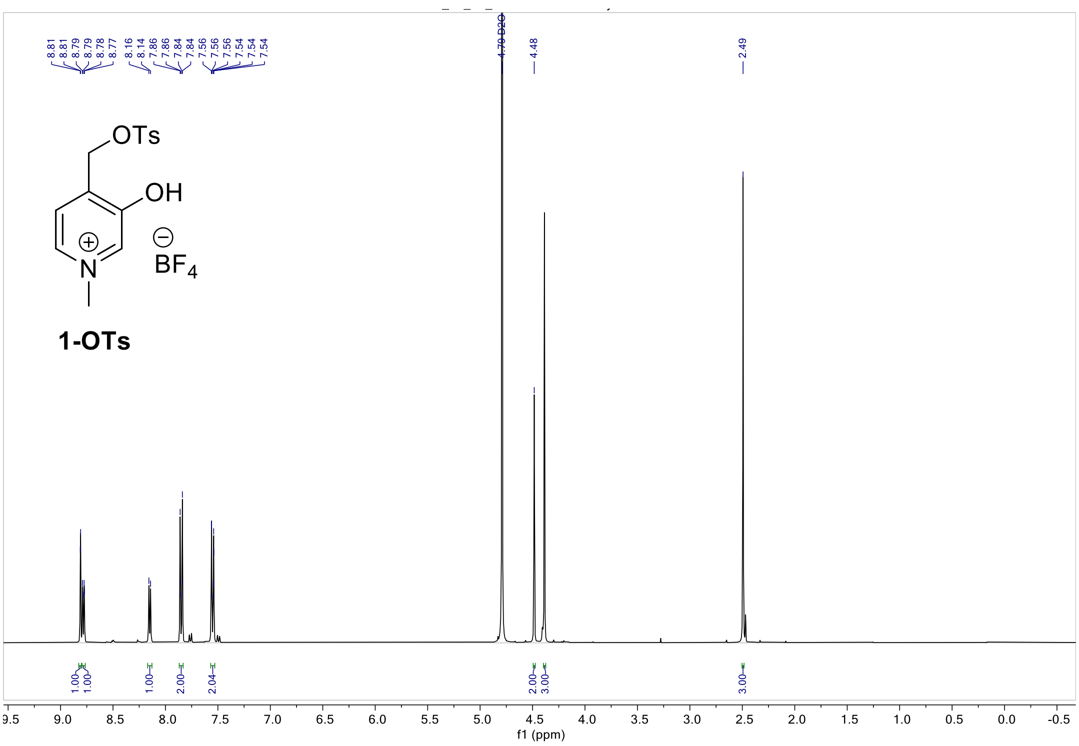

^{13}C NMR (101 MHz, D_2O) of 1-OTs

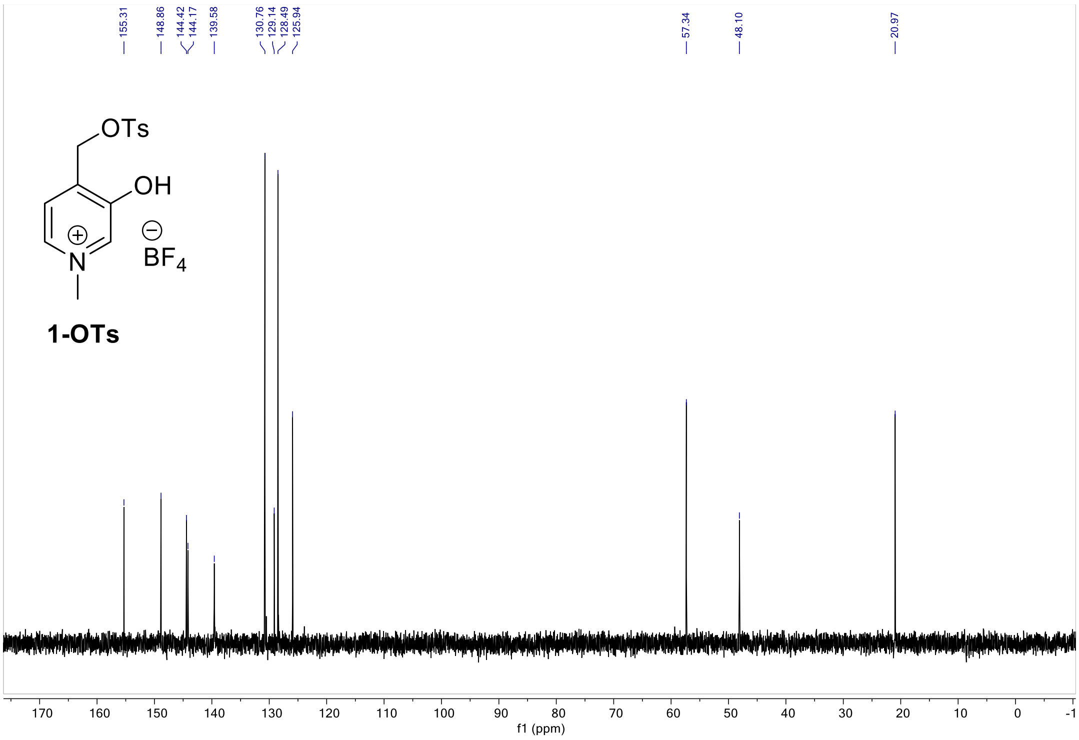

^{1}H NMR (400 MHz, D_2O) of 1-Cou

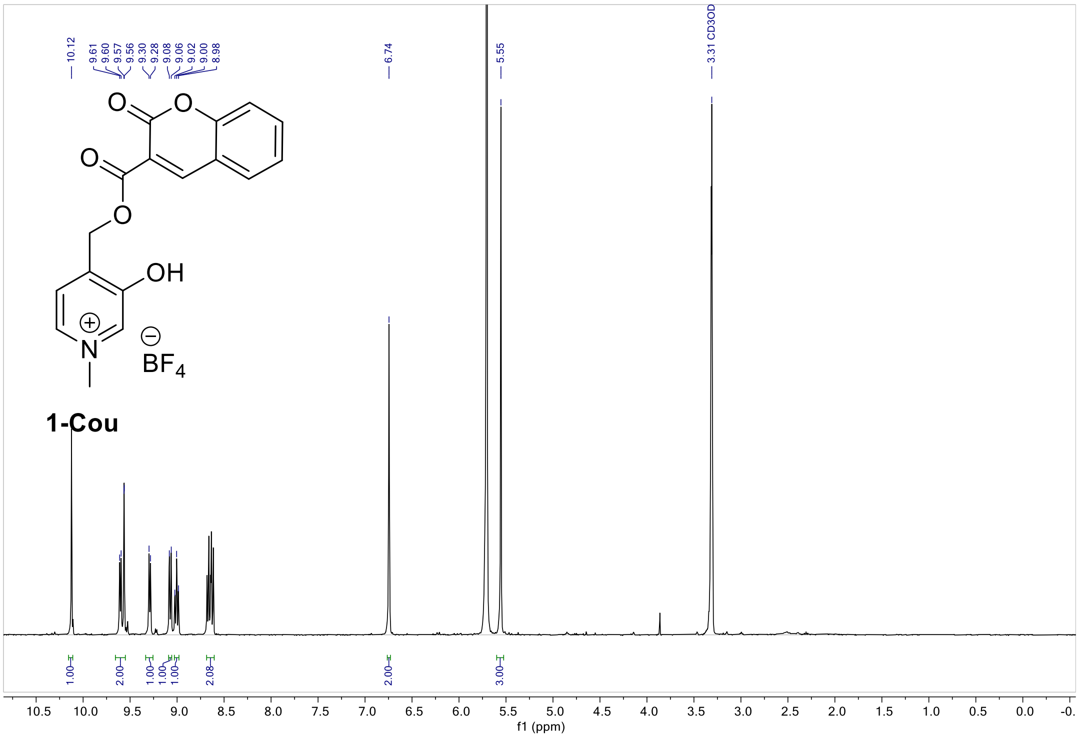

^{13}C NMR (101 MHz, $(CD_3)_2CO$) of 1-Cou

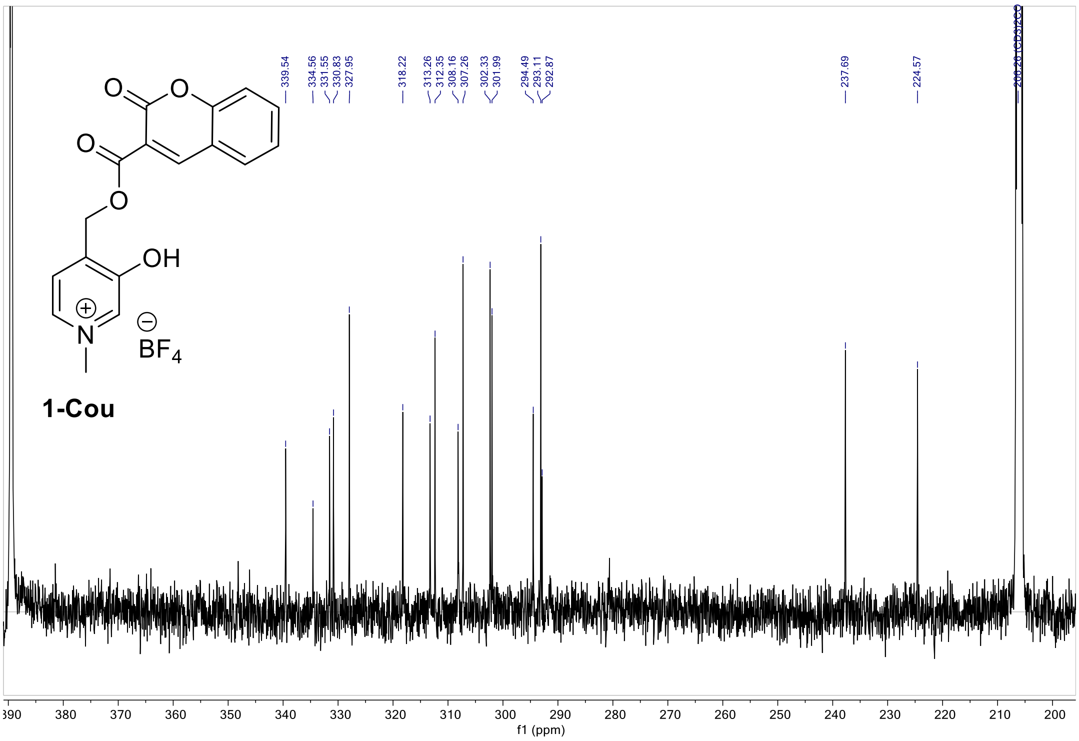

^{1}H NMR (600 MHz, CDCl3) of 2-OAc

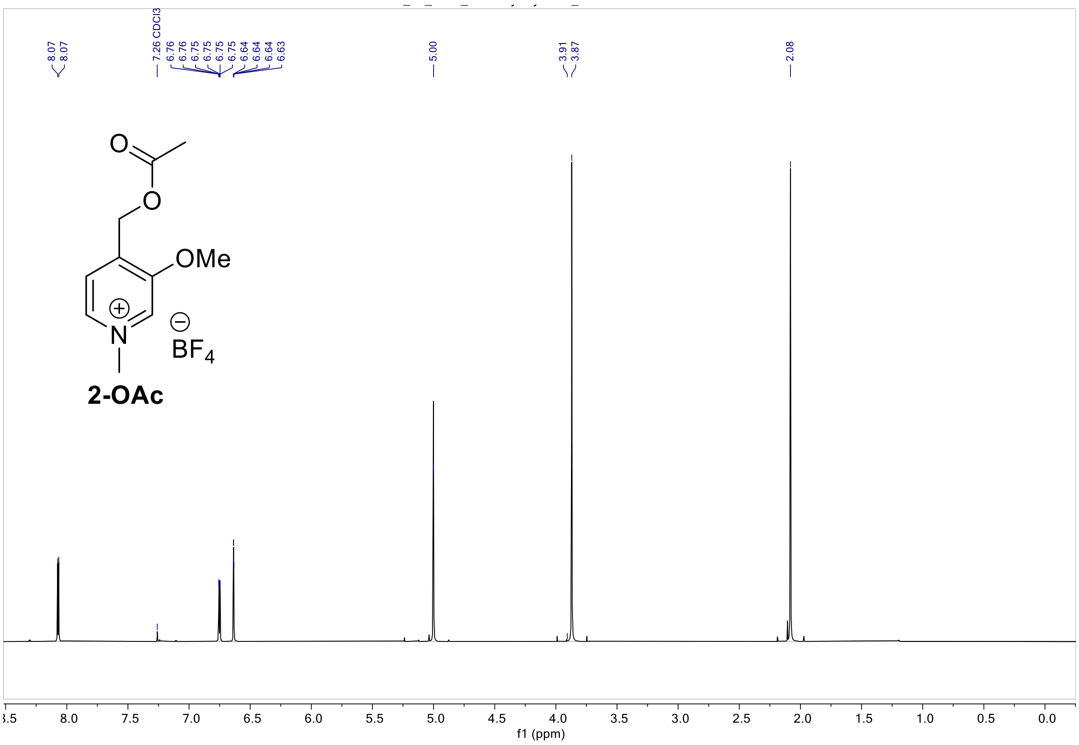

^{13}C NMR (151 MHz, CDCl3) of 2-OAc

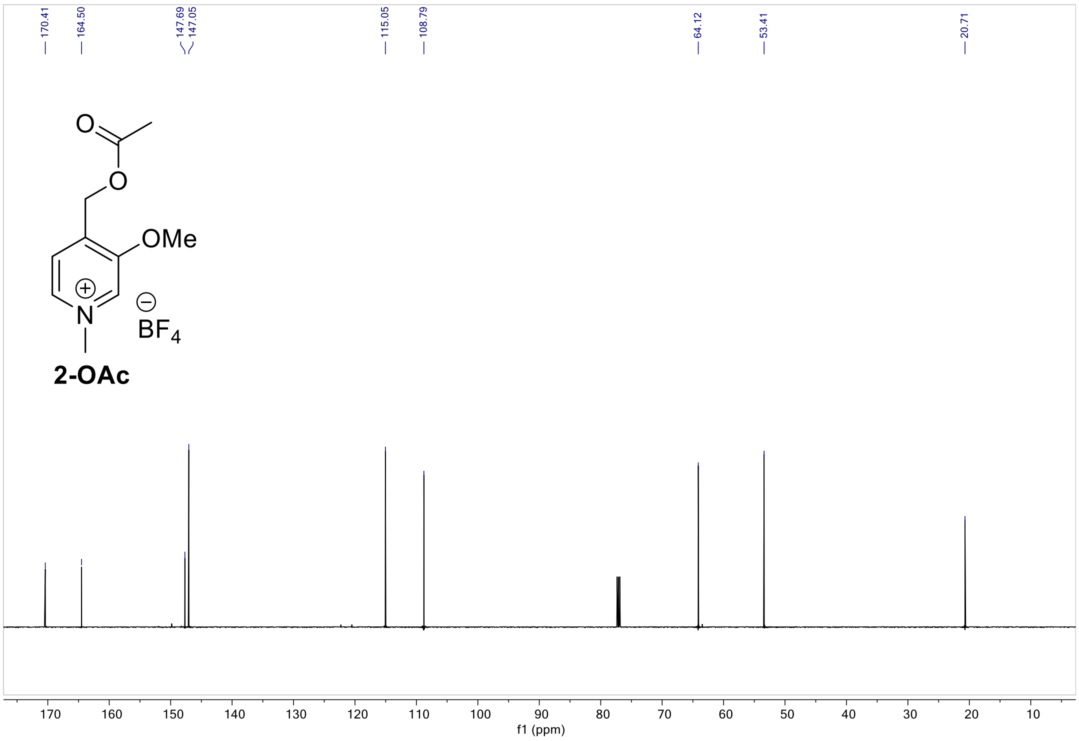

^{1}H NMR (600 MHz, D_2O) of 3-OAc

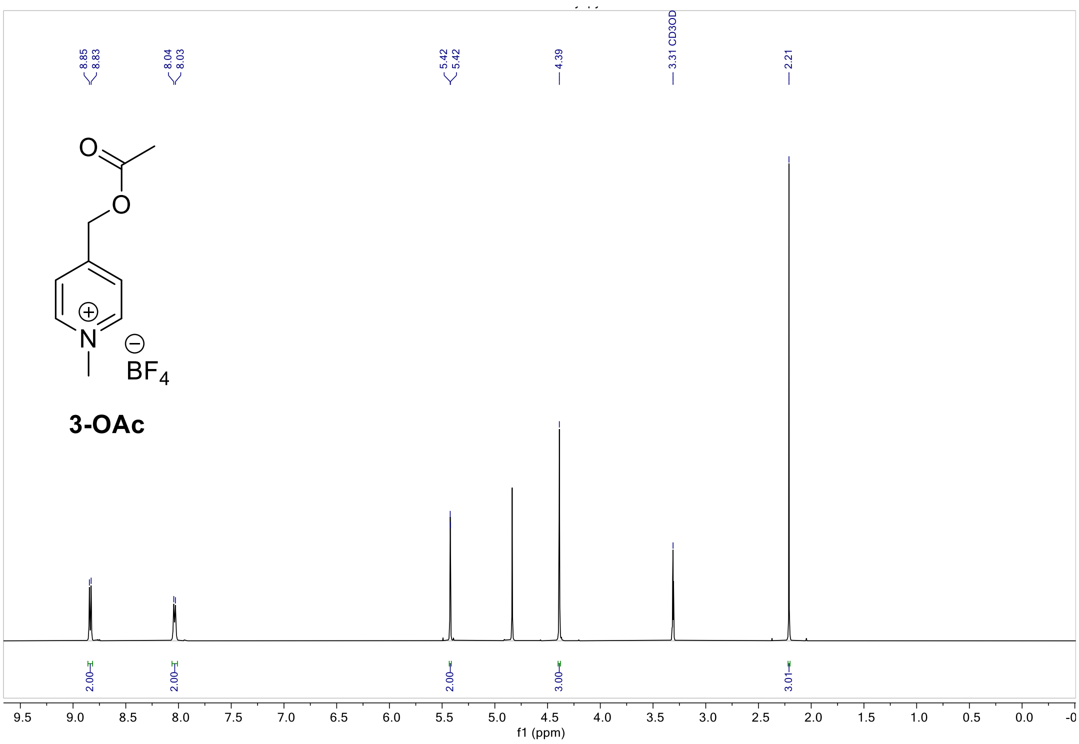

^{13}C NMR (151 MHz, D_2O) of 3-OAc

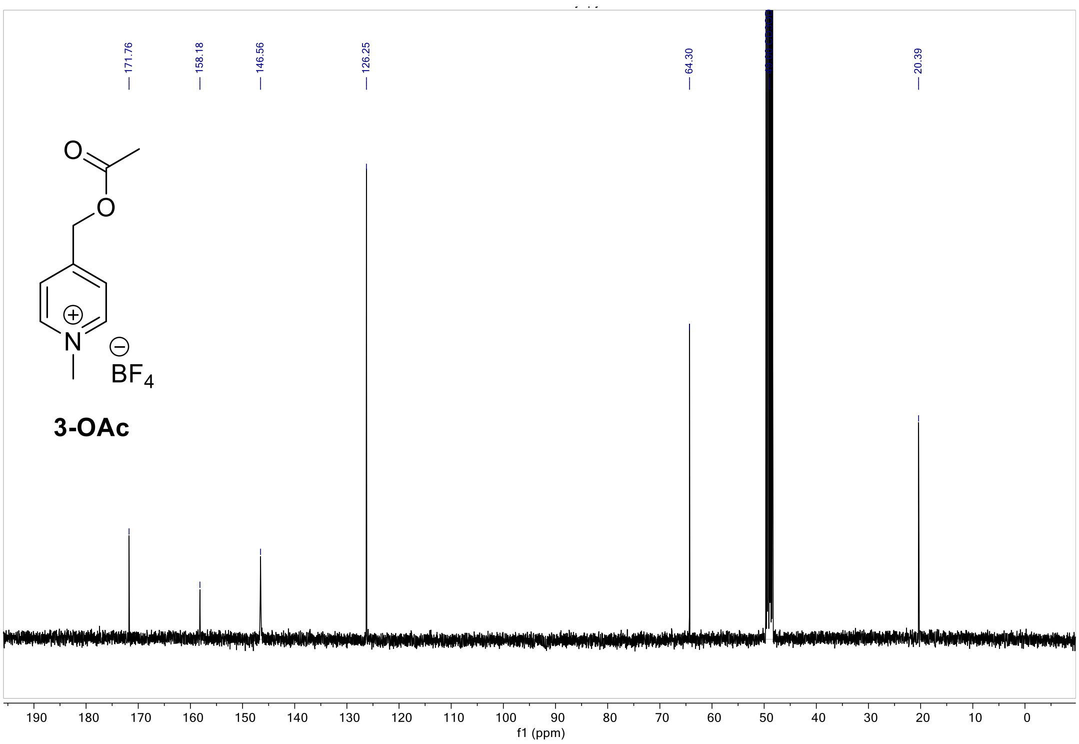

^{1}H NMR (600 MHz, CDCl3) of 1z-OH

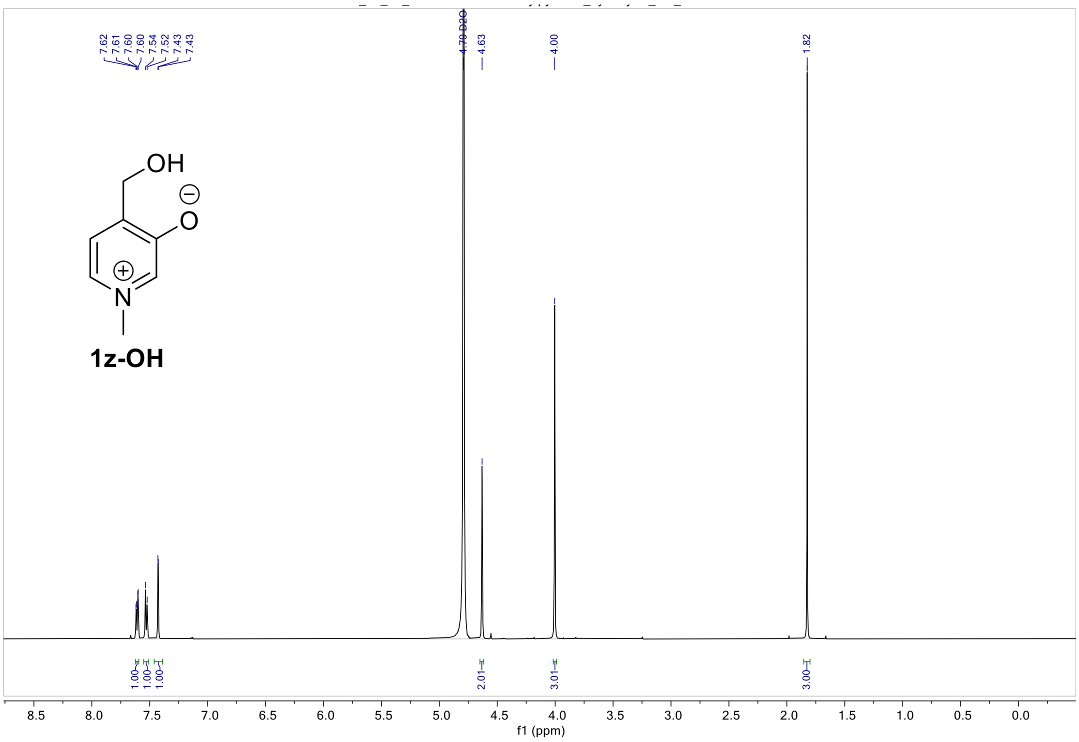

^{13}C NMR (151 MHz, CDCl3) of 1z-OH

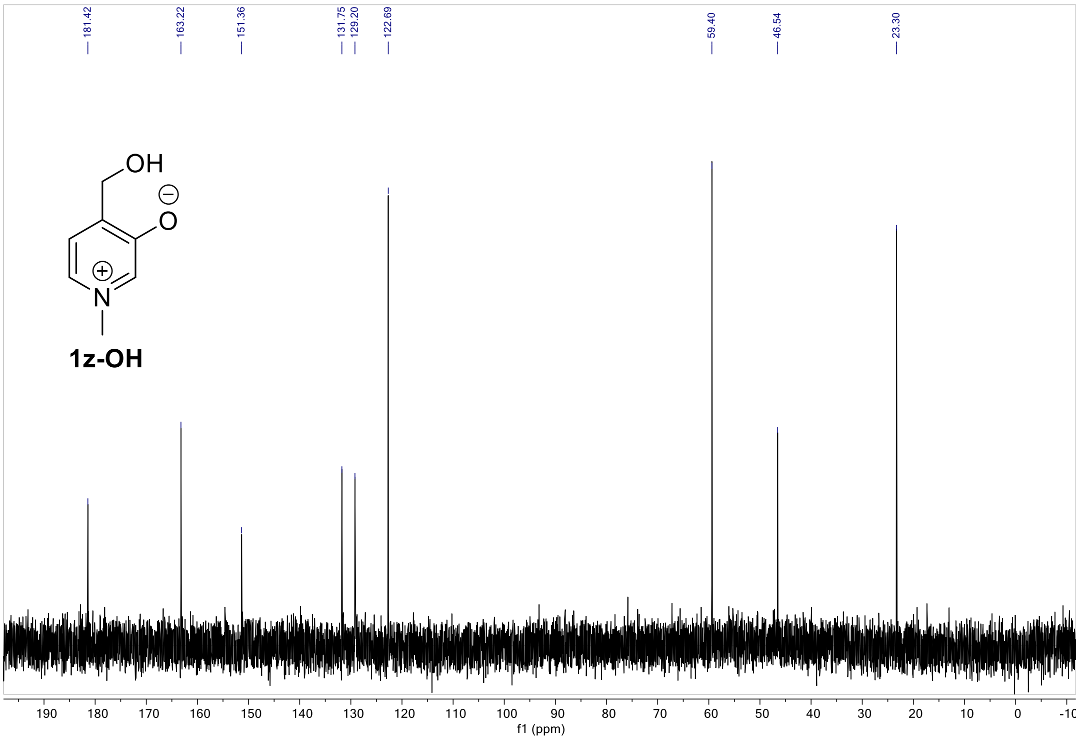

^{1}H NMR (600 MHz, MeOD) of 1-CHO

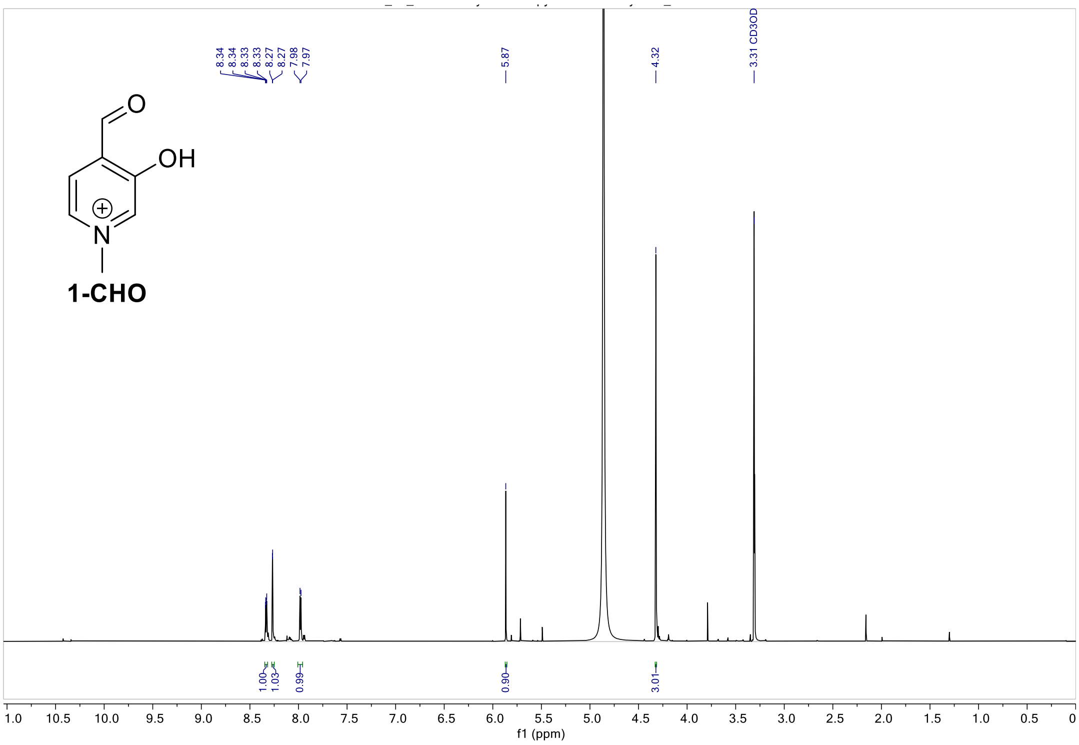

^{13}C NMR (151 MHz, MeOD) of 1-CHO

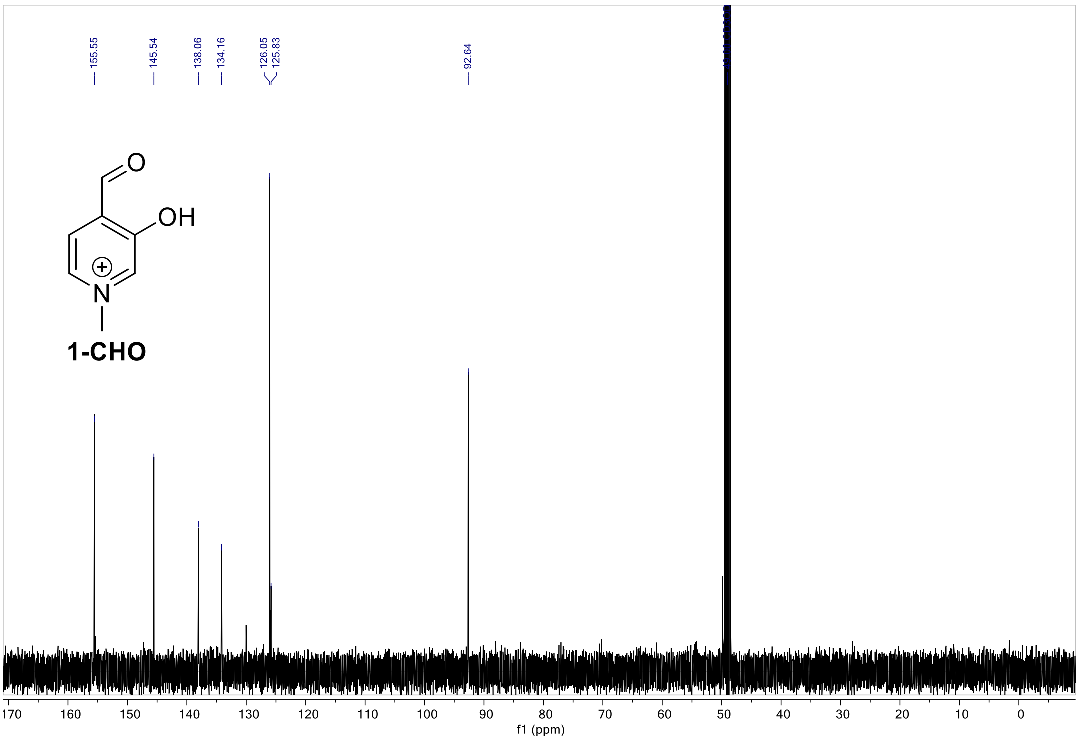

^{1}H NMR (600 MHz, D_2O) of 1-COOH

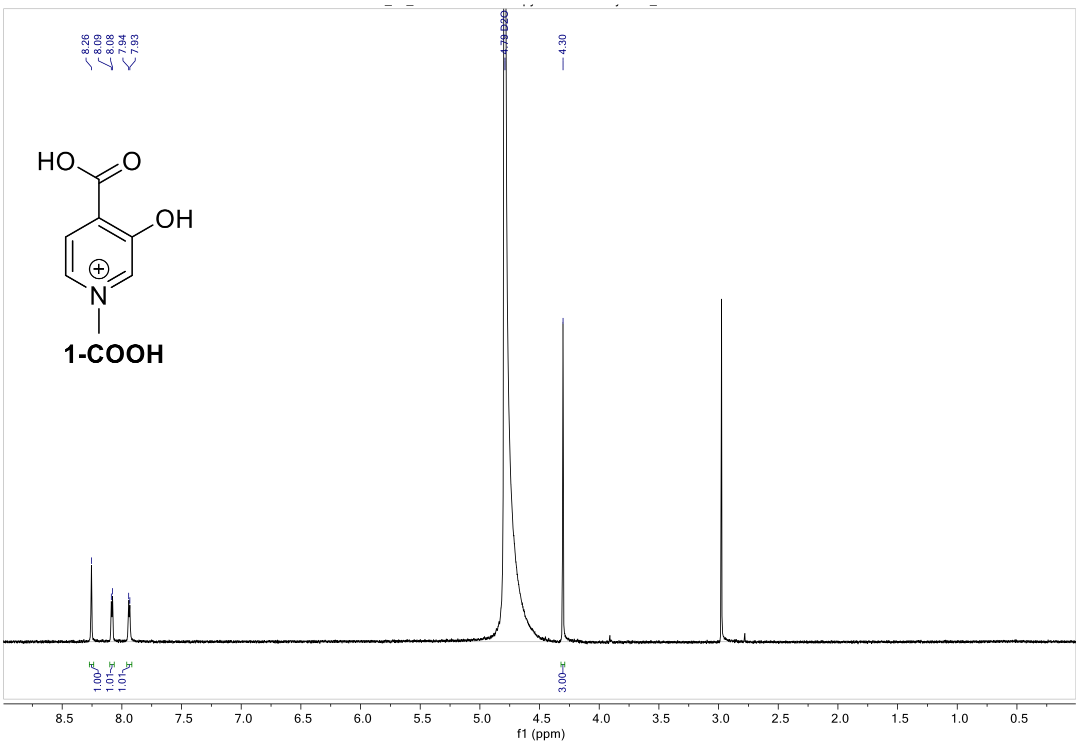

^{13}C NMR (151 MHz, D_2O) of 1-COOH

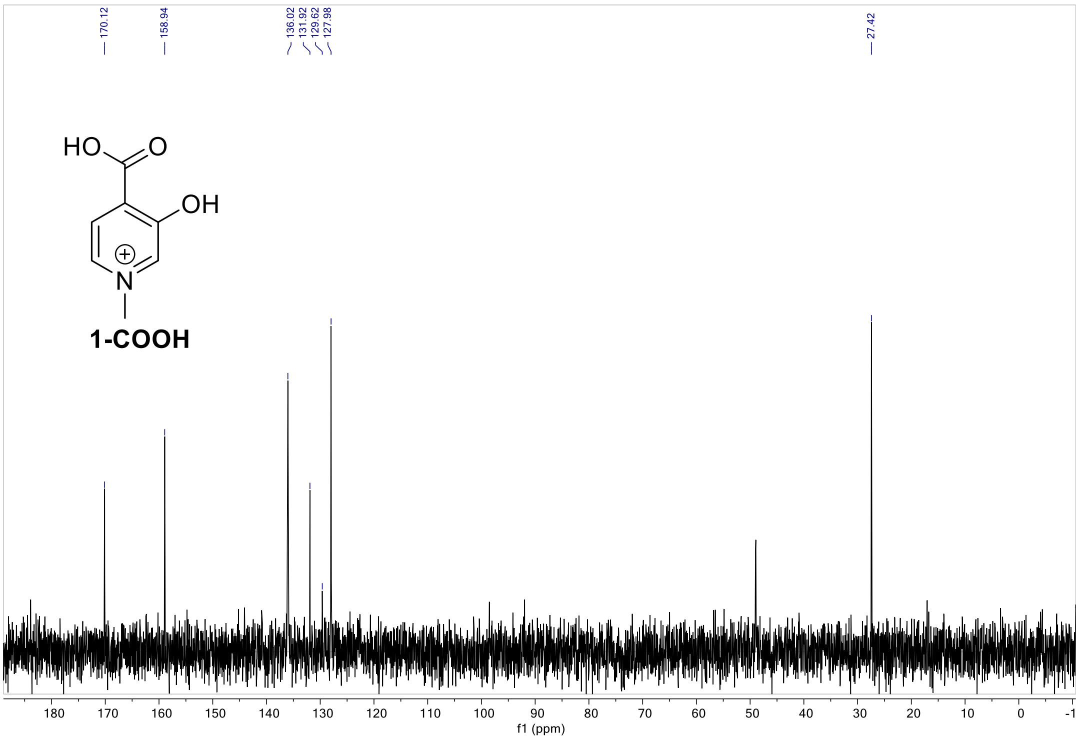

2.4 HRMS of the synthesized compounds

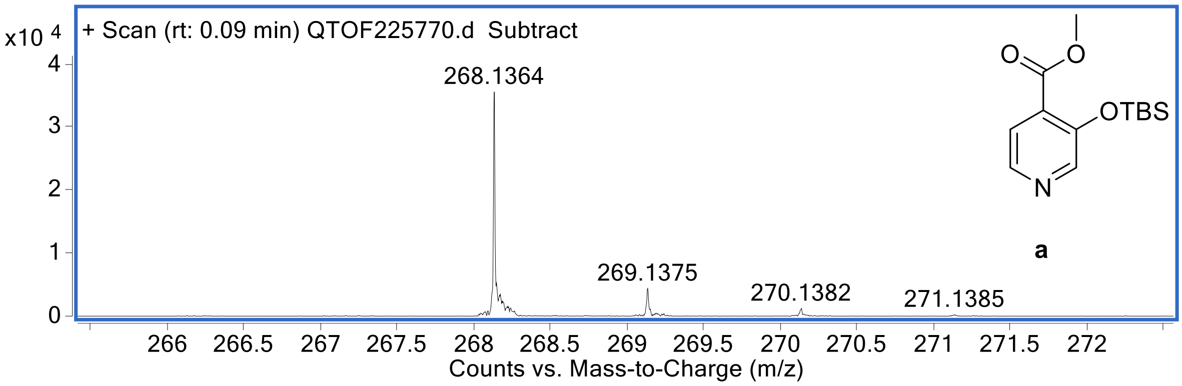

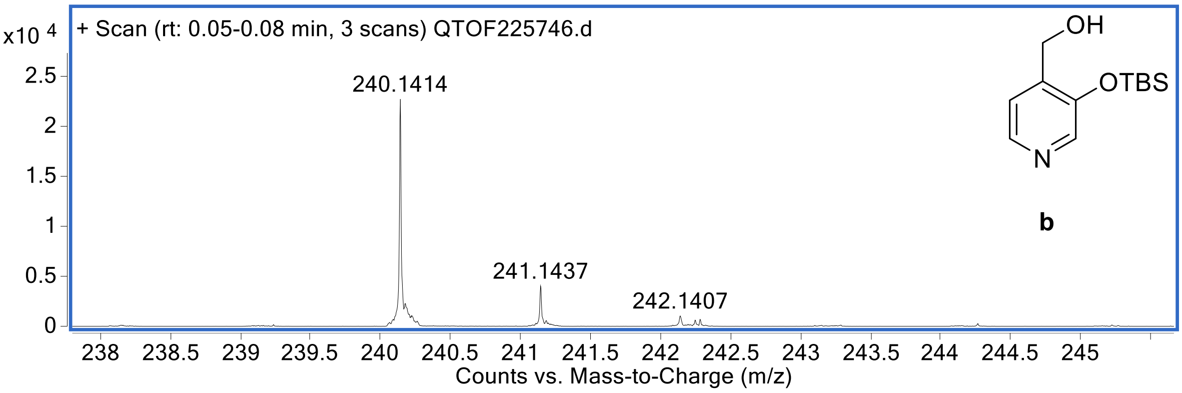

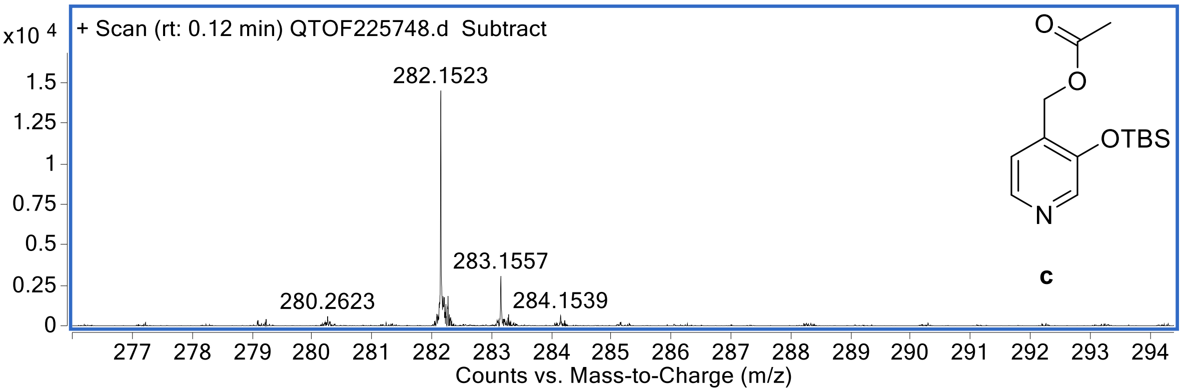

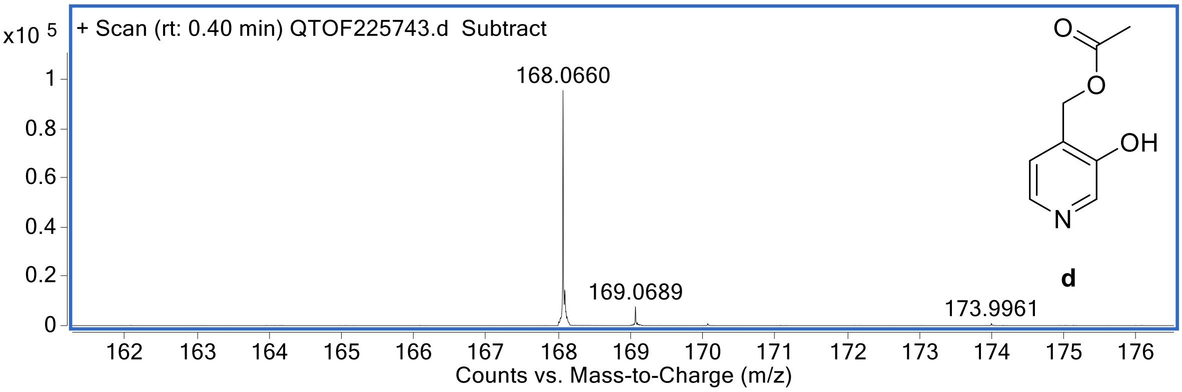

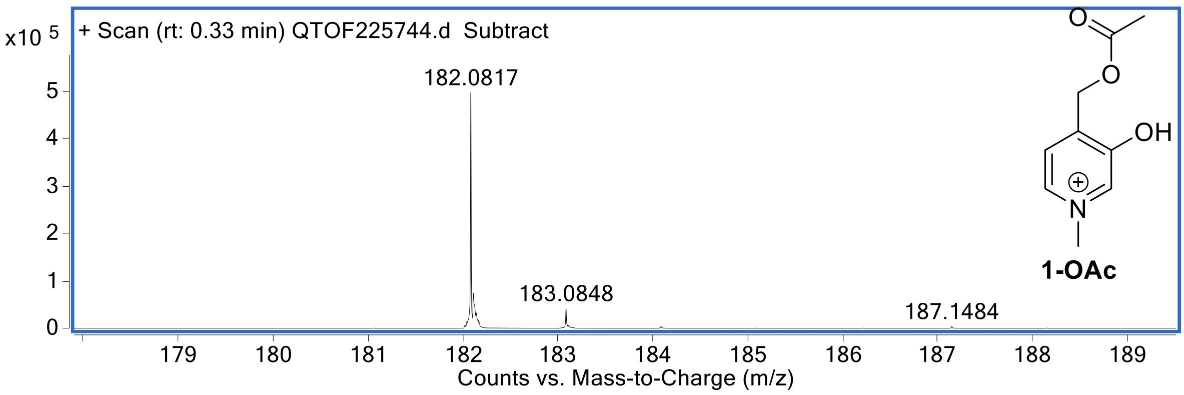
x10 5
+ Scan (rt: 0.33 min) QTOF225744.d Subtract
182.0817
183.0848
187.1484
Counts vs. Mass-to-Charge (m/z)
OH
N
1-OAc

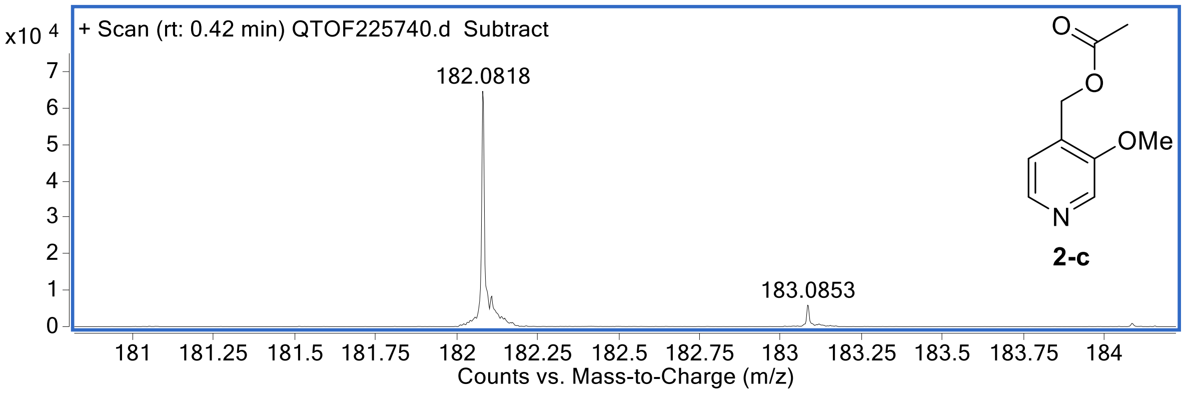
x10 4
+ Scan (rt: 0.42 min) QTOF225740.d Subtract
182.0818
183.0853
Counts vs. Mass-to-Charge (m/z)
OMe
N
2-c

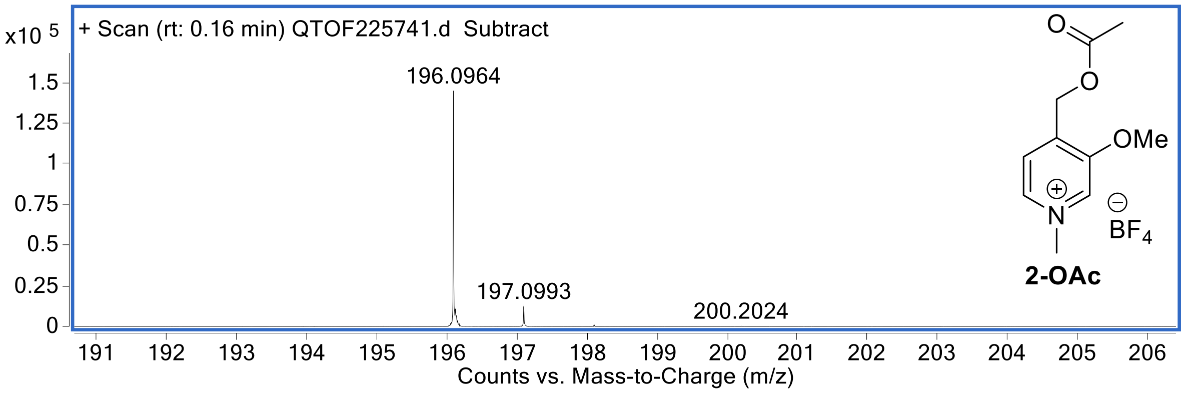
x10 5
+ Scan (rt: 0.16 min) QTOF225741.d Subtract
196.0964
197.0993
200.2024
Counts vs. Mass-to-Charge (m/z)
OMe
N
BF4
2-OAc

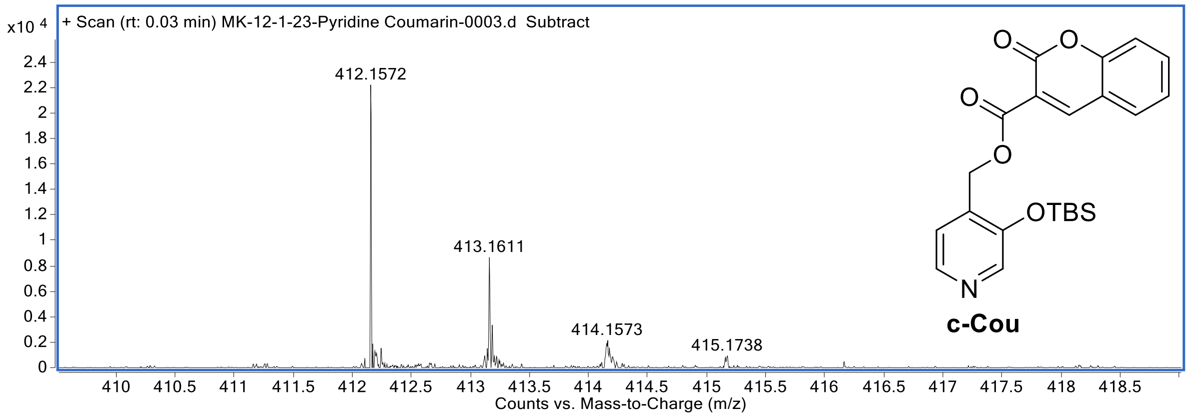
x10 4
+ Scan (rt: 0.03 min) MK-12-1-23-Pyridine Coumarin-0003.d Subtract
412.1572
413.1611
414.1573
415.1738
Counts vs. Mass-to-Charge (m/z)
OTBS
N
c-Cou

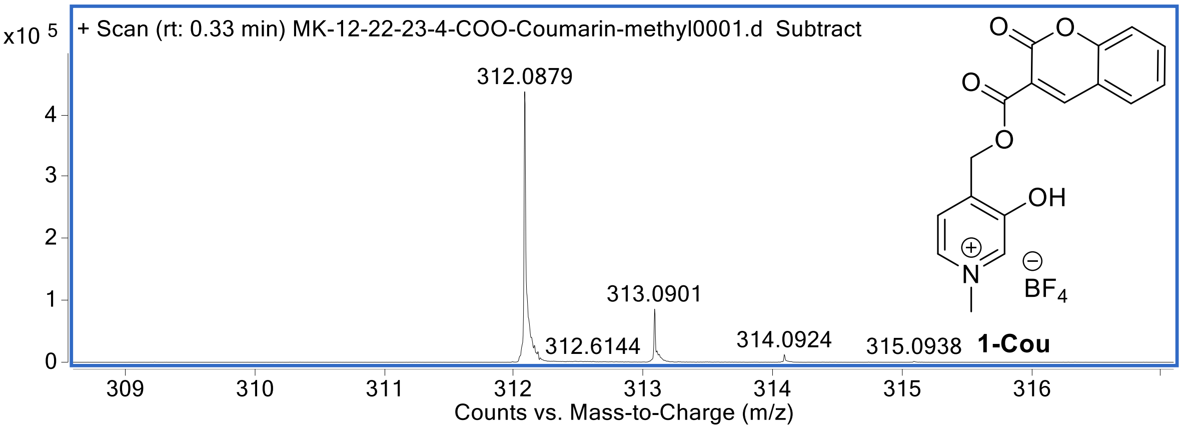

x10 5
+ Scan (rt: 0.33 min) MK-12-22-23-4-COO-Coumarin-methyl0001.d Subtract
312.0879
313.0901
312.6144
314.0924
315.0938
1-Cou
O
O
O
O
OH
N
BF4
Counts vs. Mass-to-Charge (m/z)

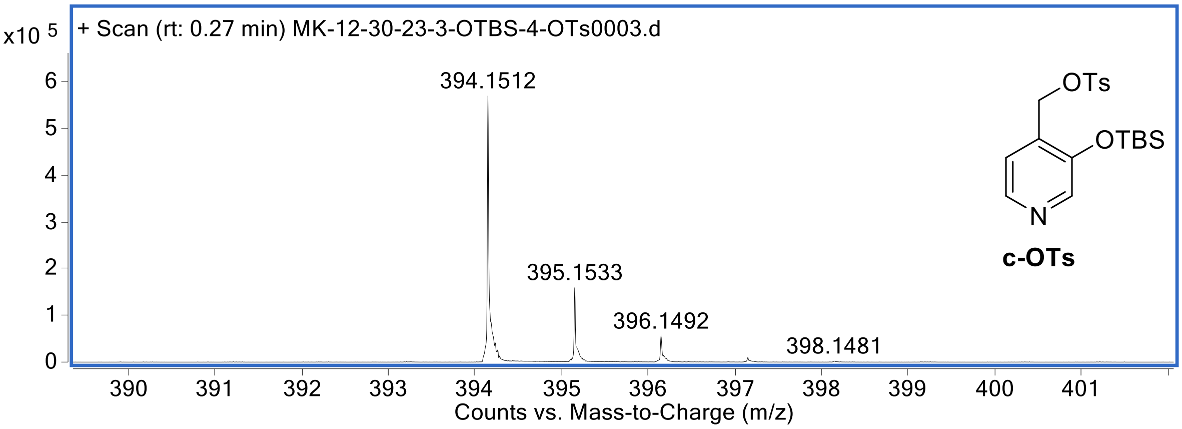

x10 5
+ Scan (rt: 0.27 min) MK-12-30-23-3-OTBS-4-OTs0003.d
394.1512
395.1533
396.1492
398.1481
OTs
OTBS
N
c-OTs
Counts vs. Mass-to-Charge (m/z)

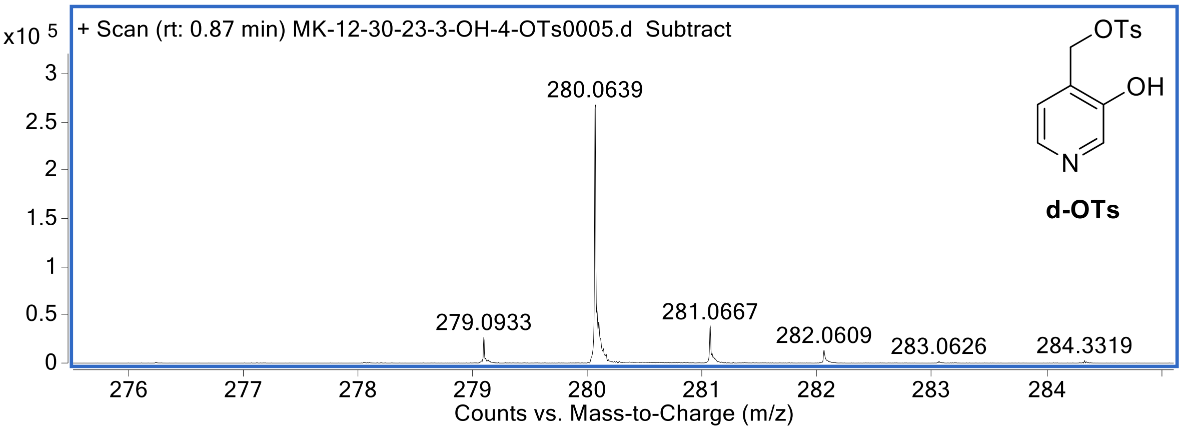

x10 5
+ Scan (rt: 0.87 min) MK-12-30-23-3-OH-4-OTs0005.d Subtract
280.0639
279.0933
281.0667
282.0609
283.0626
284.3319
OTs
OH
N
d-OTs
Counts vs. Mass-to-Charge (m/z)

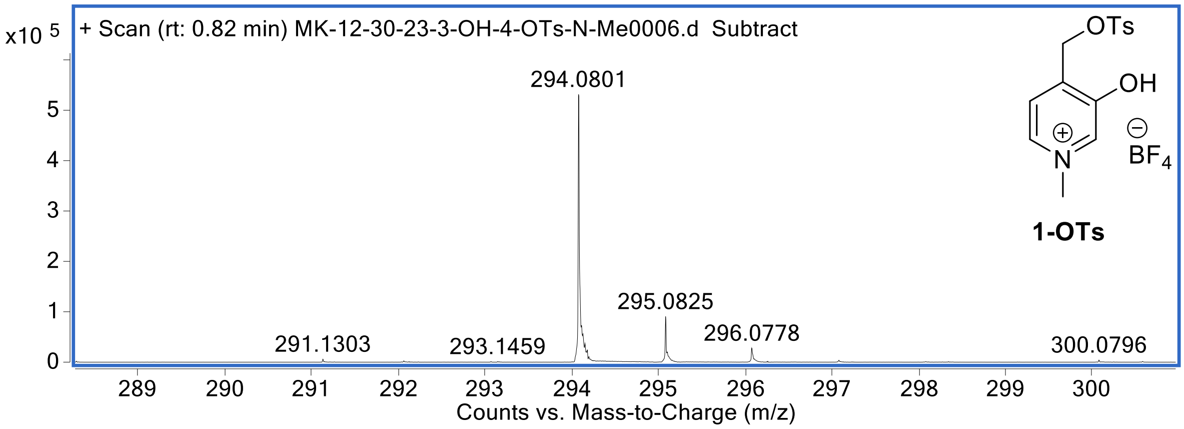
+ Scan (rt: 0.82 min) MK-12-30-23-3-OH-4-OTs-N-Me0006.d Subtract
x10 5
294.0801
295.0825
296.0778
291.1303
293.1459
300.0796
OTs
OH
N
BF4
1-OTs
Counts vs. Mass-to-Charge (m/z)

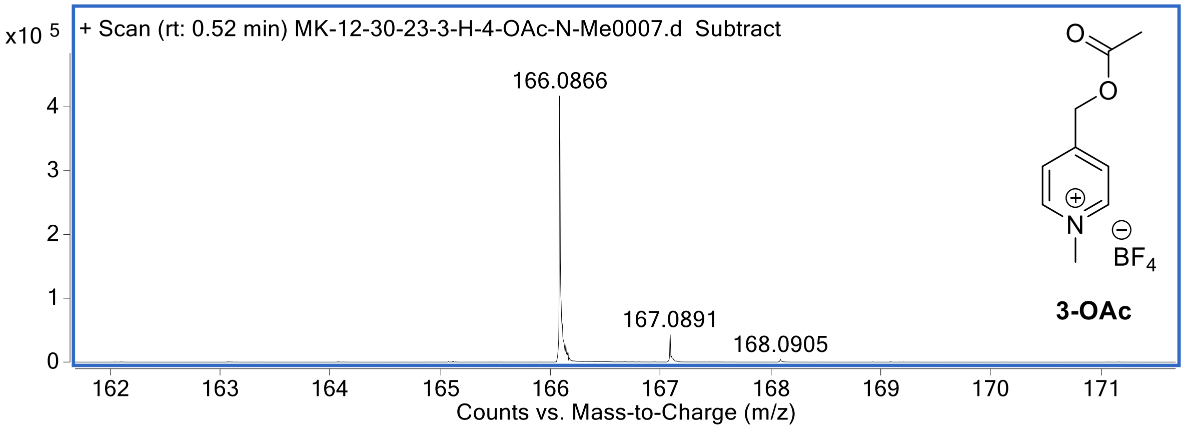
+ Scan (rt: 0.52 min) MK-12-30-23-3-H-4-OAc-N-Me0007.d Subtract
x10 5
166.0866
167.0891
168.0905
O
O
N
BF4
3-OAc
Counts vs. Mass-to-Charge (m/z)

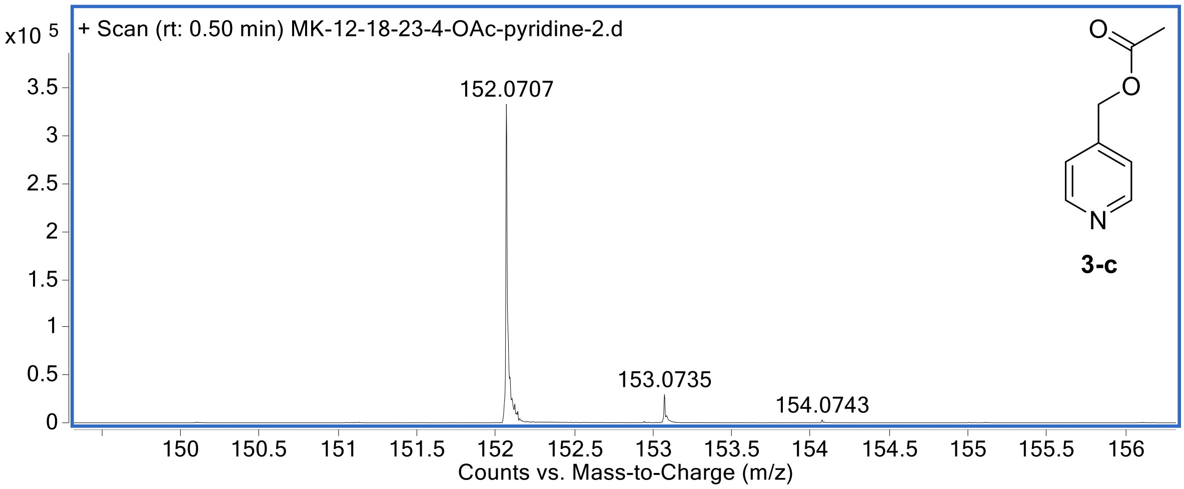
+ Scan (rt: 0.50 min) MK-12-18-23-4-OAc-pyridine-2.d
x10 5
152.0707
153.0735
154.0743
O
O
N
3-c
Counts vs. Mass-to-Charge (m/z)

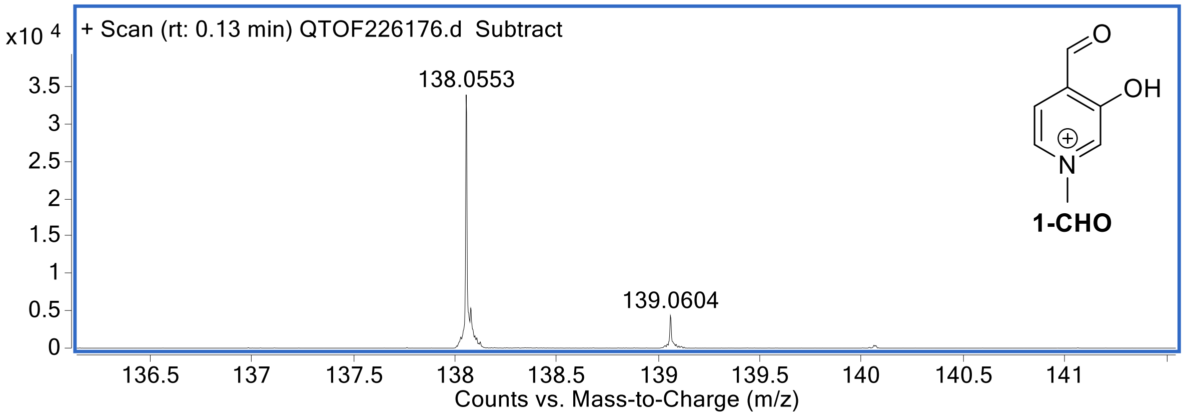

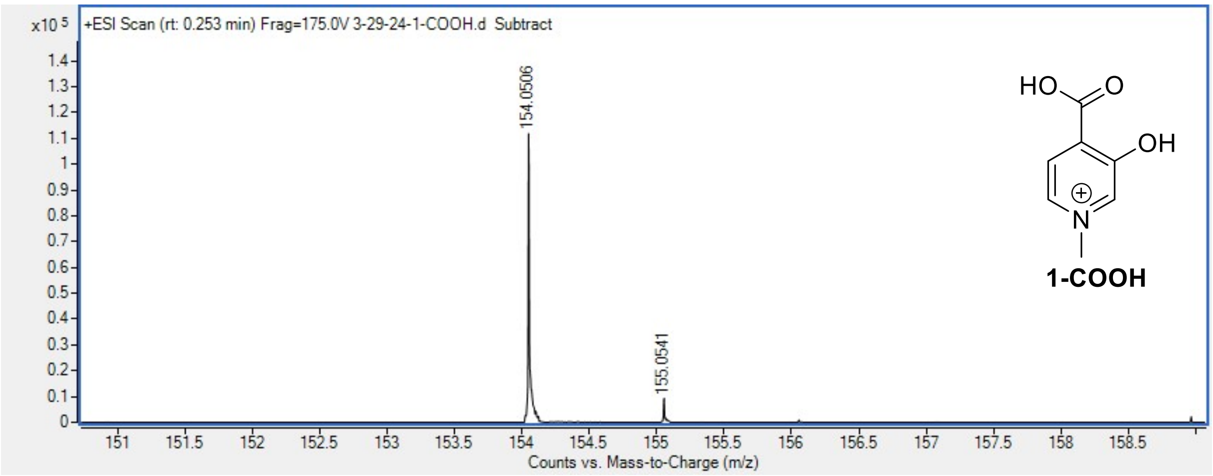

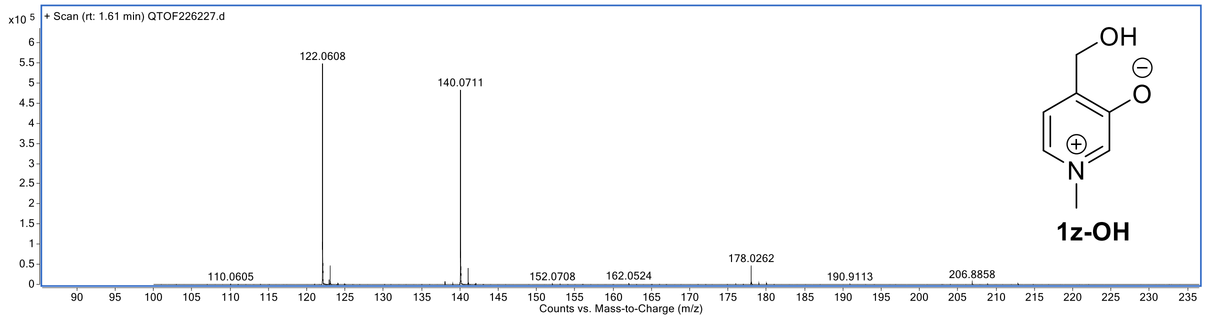

3 Photorelease studies

3.1 General information

Quantum yield of release studies

Procedure for quantum yield of release was adopted from previously published work.[1,2] Quantum yield of release was determined by quantitative ^{1}H NMR, Rayonet Photochemical Reactor (an older version of Rayonet RMR-600 Photochemical Reactor) equipped with four 6

watt 300 nm (for 1-OAc, 1z-OAc, 2-OAc & 1-Cou) and 254 nm (for 1-OTs and 3-OAc) lamps as the irradiation source, and potassium ferrioxalate as the actinometer.

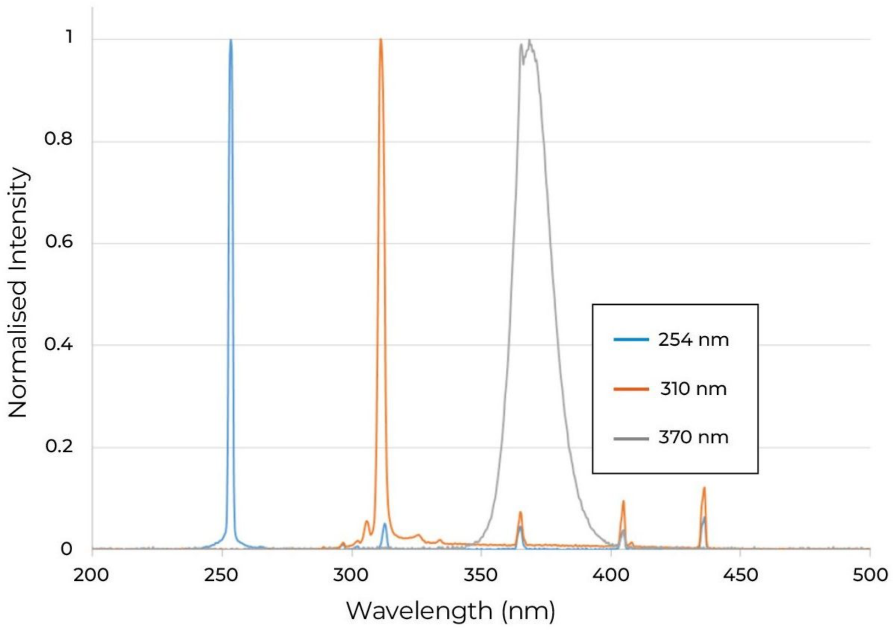

Measurements for actinometer:

Preparation and manipulation of ferrioxalate solutions and samples were carried out in a dark room equipped with a red light. To prepare the actinometer three volumes of 1.5 M $K_2C_2O_4$ solution were mixed with one volume of 1.5 M $FeCl_3$ solution in water. The precipitated $K_3Fe(C_2O_4)_3{\cdot}3H_2O$ was recrystallized thrice with hot water and dried at room temperature. The ferrioxalate solution was prepared by dissolving 2.95 g of the precipitate in 800 mL water and 100 mL of 1.0 N H_2SO_4 followed by dilution with water to 1L. Additionally, a developer solution (0.1%, w/v 1,10-phenanthroline in water) and a buffer solution (0.6 N sodium acetate and 0.36 N H_2SO_4 in water) were used to prepare samples for absorbance measurements.

For each measurement, a quartz cuvette was filled with 3.00 mL of 0.006 M ferrioxalate solution and irradiated for a set of periods of time (10, 20, 30, 40, 50 seconds). Irradiated solution was transferred to a 25 mL volumetric flask, to which was added, in sequence, 4 mL of the developer solution and 1.5 mL of the buffer solution. The sample was diluted to 25 mL with water, mixed, and incubated for 30 minutes. After the incubation period, a sufficient amount of sample was transferred into a 1 cm methacrylate cuvette, and the absorbance at 510 nm was read using a UV-Vis spectrophotometer. The 0-second irradiation samples were used as the blanks.

A standard curve was prepared using $FeSO_4$ solutions ranging from 1.8×10^{-5} M to 1.1×10^{-4} M as follows. Varying amounts of a 4.5×10^{-4} M $FeSO_4$ in 0.1 N H_2SO_4 stock solution (0, 1, 2, 3, 4, 5, 6 mL) were transferred into a 25 mL volumetric flask. To this, sufficient amounts of 0.1

N H_2SO_4 in water solution (12.5, 11.5, 10.5, 9.5, 8.5, 7.5 mL) were added followed by 5 mL of the developer solution, 6.25 mL of the buffer, and water to dilute to the mark. The solutions were incubated for 30 minutes before transferring into a 1cm methacrylate cuvette. The absorbance of the solutions was read at 510 nm using a UV-Vis spectrophotometer.

The flux of the Photochemical Reactor was calculated using the following equations:

$$I = \frac{\Delta n}{10^{-3} \cdot \Phi \cdot V_1 \cdot t}$$

where I is the flux (Einstein/L/s), Δn is the moles of Fe^{2+} photogenerated, Φ is the quantum yield at 365 nm, V_1 is the irradiated volume (mL), and t = irradiation time (seconds).

$$\Delta n = \frac{10^{-3} \cdot V_1 \cdot V_3 \cdot C_T}{V_2}$$

where V_2 is the volume taken from the irradiated sample (mL), V_3 is the volume after dilution for concentration determination (mL), and C_T is the concentration of Fe^{2+} after dilution (M).

$$C_T = \frac{abs}{\varepsilon \cdot l}$$

where abs is the absorbance at 510 nm, ε is the molar absorptivity (M^{-1} cm^{-1}), and l is the path length.

Quantitative ^1H NMR:

Photorelease of the compounds were measured using quantitative 1H NMR (AV III 600 MHz) in either buffer solutions prepared with D_2O at pH 2 or in a pH 7, where the pulse angle was 90° and the recycling delay cycle was 20 seconds.

3-5 mM solutions of the compounds were prepared with dimethyl sulfone as the internal standard. The absorbance of the solution was measured at 300 nm (1-OAc, 1z-OAc, 2-OAc) and 254 nm (for 1-OTs and 3-OAc) using UV-Vis spectrophotometry to ensure the absorbance is higher than 2. A 3mL portion of the prepared sample was transferred into a 4-sided quartz cuvette and was irradiated while stirring for a period. After irradiation, 0.5 mL of the sample was transferred to a NMR tube. The amounts of the leaving group released was quantified using ^1H NMR. The sample was then transferred back to the cuvette and irradiation was continued. The resulting quantum yields were determined as an average of three independent runs.

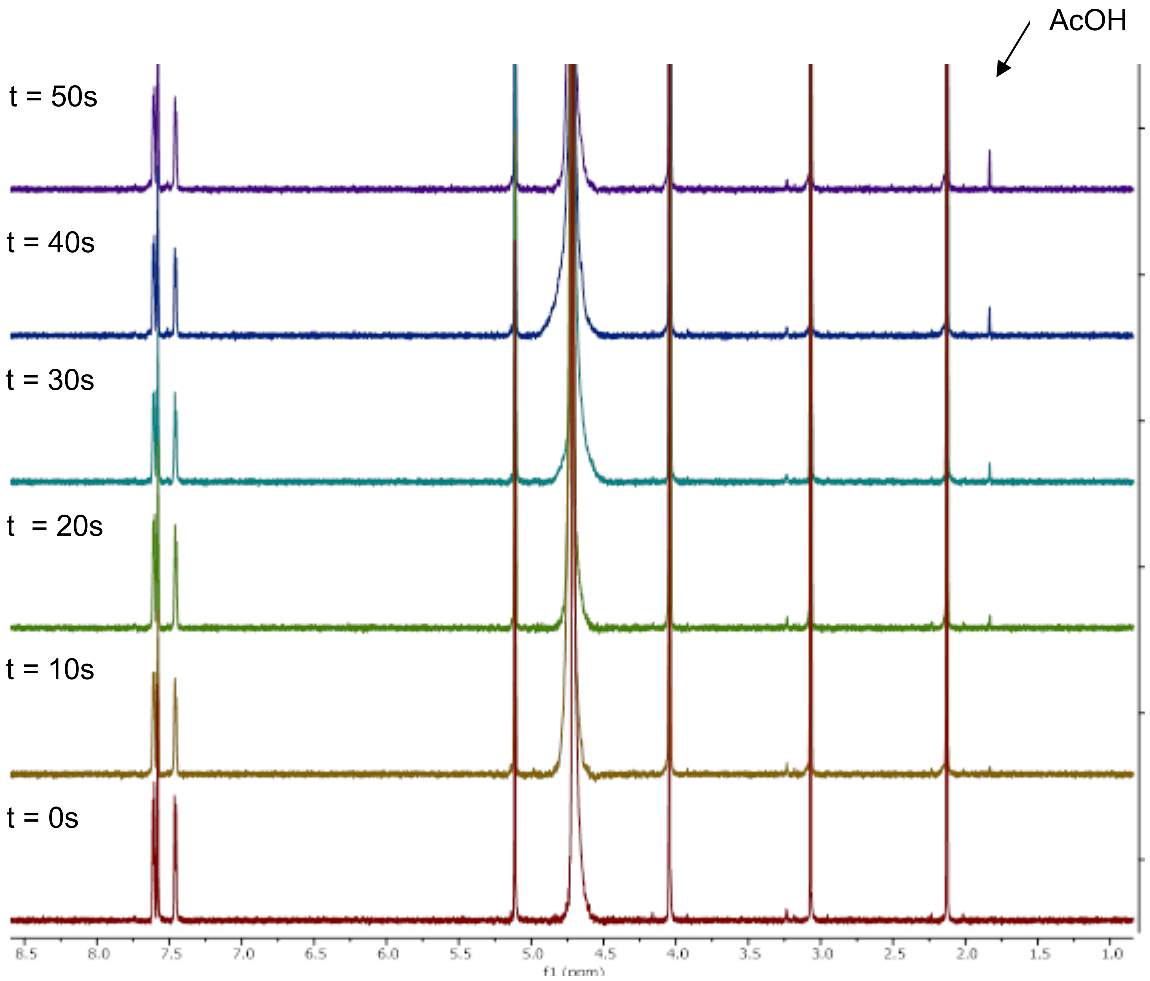

Figure S 1. Photorelease of acetic acid from **1z-OAc** with irradiation.

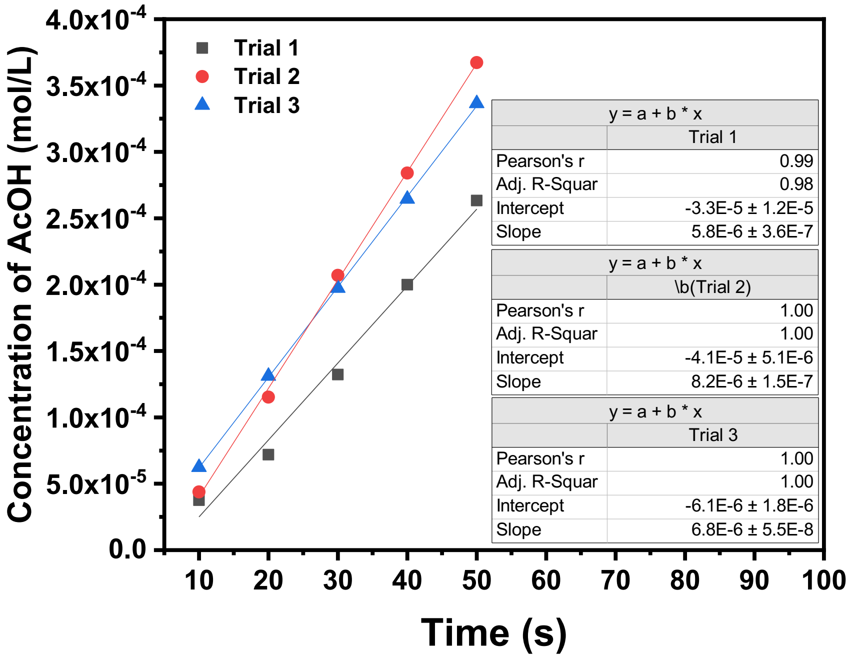

Figure S 2. An example graph for Φ_r calculation (**1z-OAc**).

3.2 Molar Absorption Coefficient determination

The molar extinction coefficient was determined by preparing using the corresponding buffer solution (in D_2O) containing a precise concentration of the internal standard, dimethyl sulfone (DMS). A known quantity of the compound of interest was dissolved in 1 mL of deuterated chloroform, and subsequently, the ^1H nuclear magnetic resonance (NMR) spectrum was recorded. The concentration of the compound of interest was then determined by referencing it against the concentration of the internal standard.

Following this determination, the stock solution was diluted with the respective buffer solution as the solvent, resulting in samples with varying concentrations. A graph correlating absorbance (y-axis) with concentration (x-axis) was plotted, utilizing a minimum of five distinct solutions, each featuring an absorbance falling within the 0.1 to 1 range. The gradient of the resulting line on the graph provides the molar extinction coefficient.

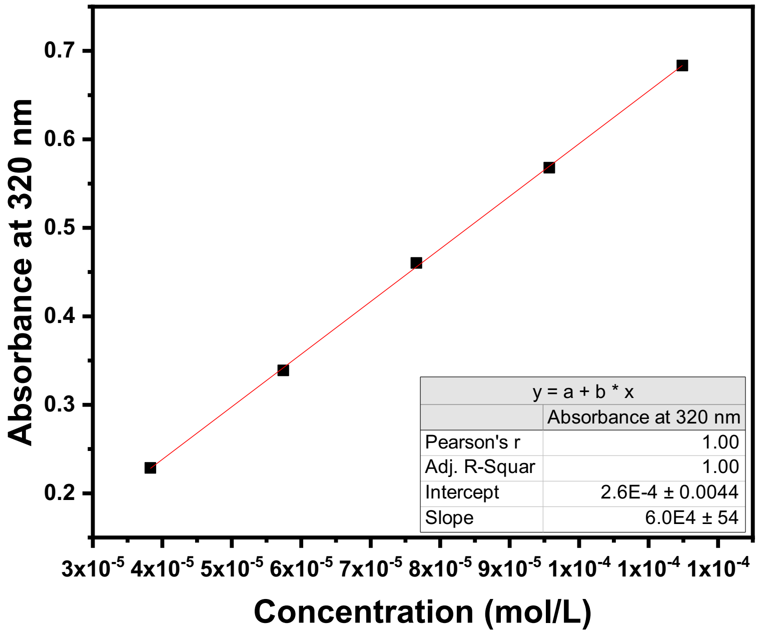

Figure S 3. An example graph for Molar absorption coefficient determination (**1z-OAc**).

3.3 Photorelease under air and inert conditions

Two 0.5 mM samples of 1z-OAc were prepared in pH 7 Buffer (D_2O). One of the samples underwent purging with N_2 for 30 minutes in the dark. Subsequently, both samples were irradiated, and the release of AcOH was quantified using ^1H NMR. The liberated AcOH concentrations were plotted against time, and the two gradients of the resulting curves were compared to determine the impact of oxygen on the release process.

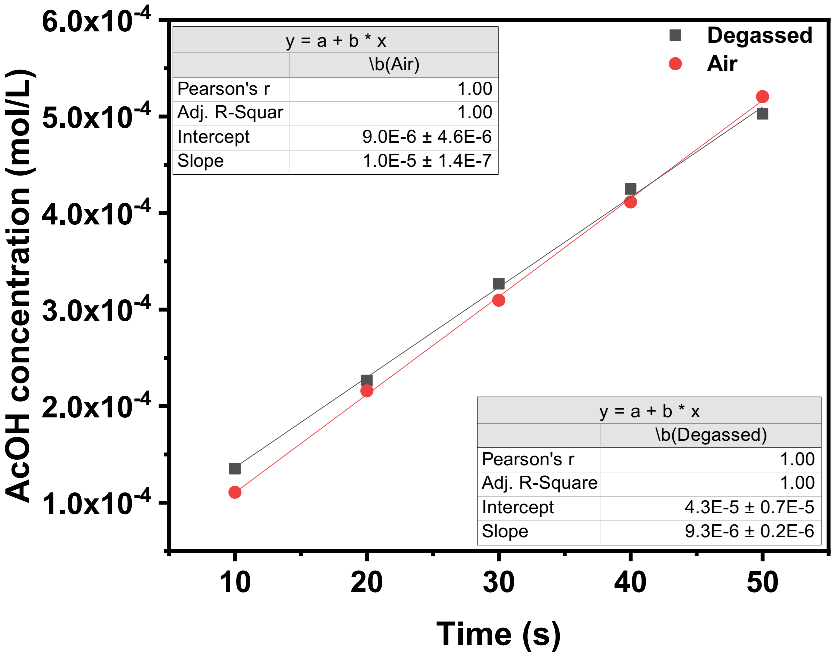

Figure S 4. The graph for the determination of the impact of oxygen on the photorealism of **1z-OAc**.

A similar experiment was conducted where the experiment focused on monitoring the generation of 1-CHO using absorption spectroscopy, with a calibration curve for 1-CHO in pH 7 buffer. After irradiation, absorbance data were collected over time and a time vs 1-CHO concentration was plotted.

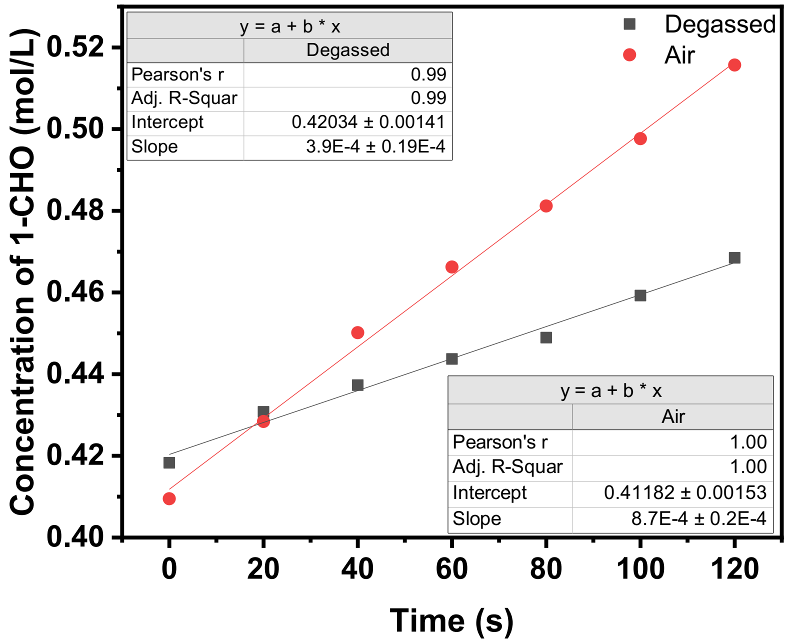

Figure S 5. The graph for the determination of the impact from oxygen on the generation of **1-CHO**.

3.4 Thermal stability of the compound 1z-OAc

Table S1: Thermal stability of compound **1z-OAc**.

Time/h	% Hydrolyzed
0	0
8	0.39
12	0.57
24	1.20

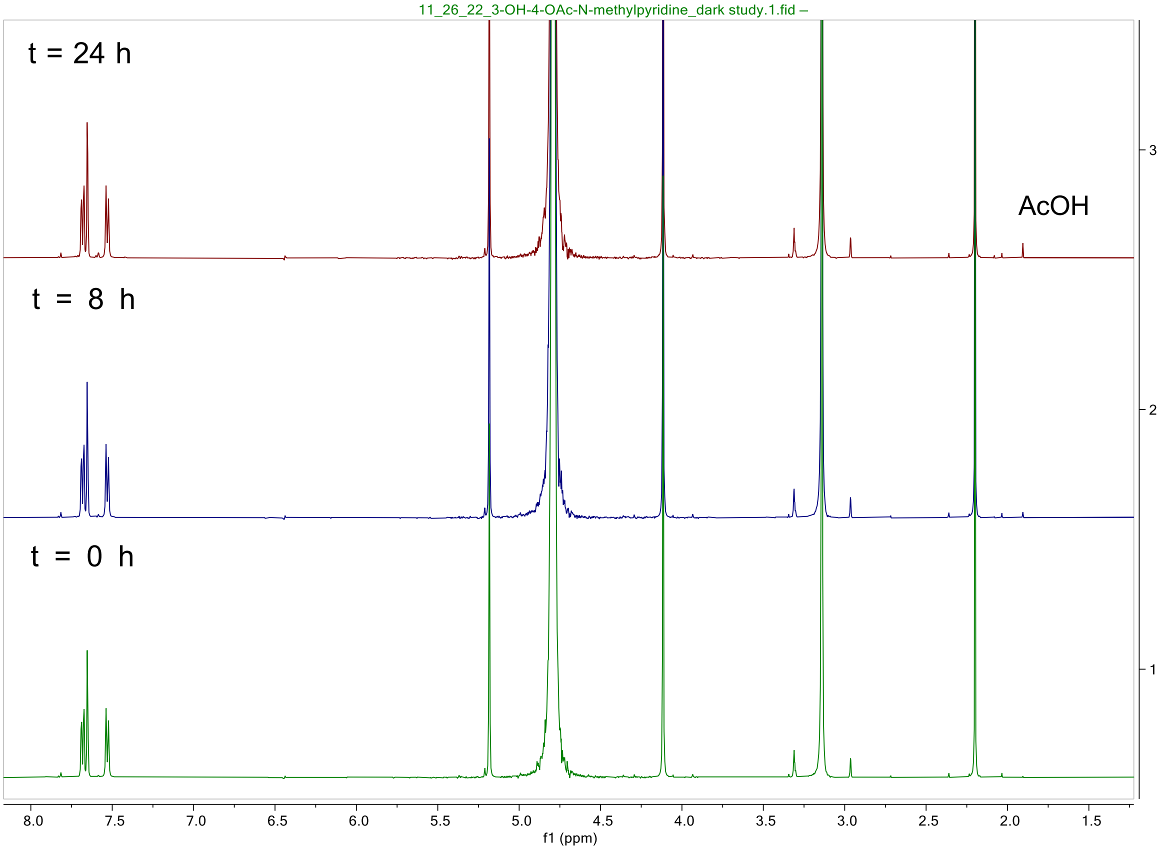

Figure S 6. Release of acetic acid from **1z-OAc** at room temperature in dark.

3.5 Photoproduct analysis

3.5.1 LC-MS studies

A 0.5 mM sample of **1z-OAc** was irradiated for 20 minutes with 5-minute intervals to avoid heating of the instrument. The resulting solution was subjected to LC-MS analysis.

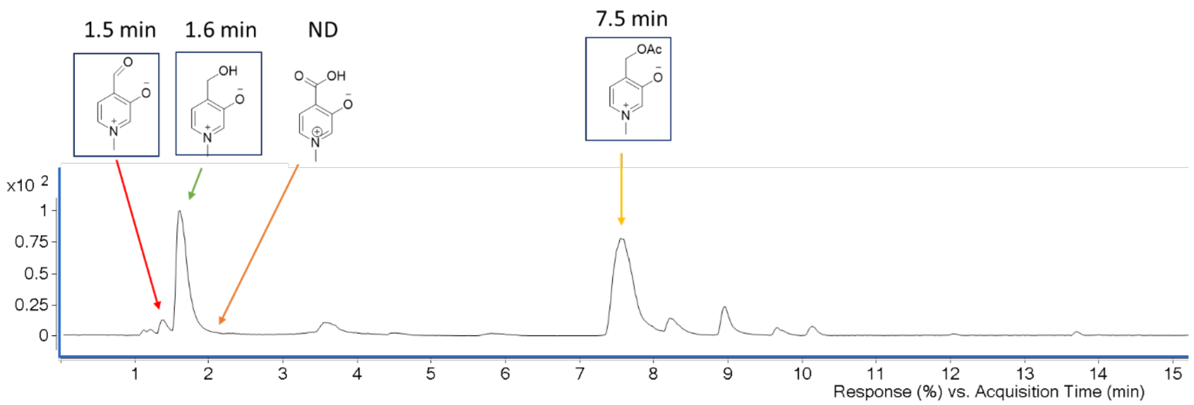

Figure S 7. Photoreaction monitored by LC-MS. Chromatogram based on TIC.

3.5.2 NMR Spectroscopic Studies

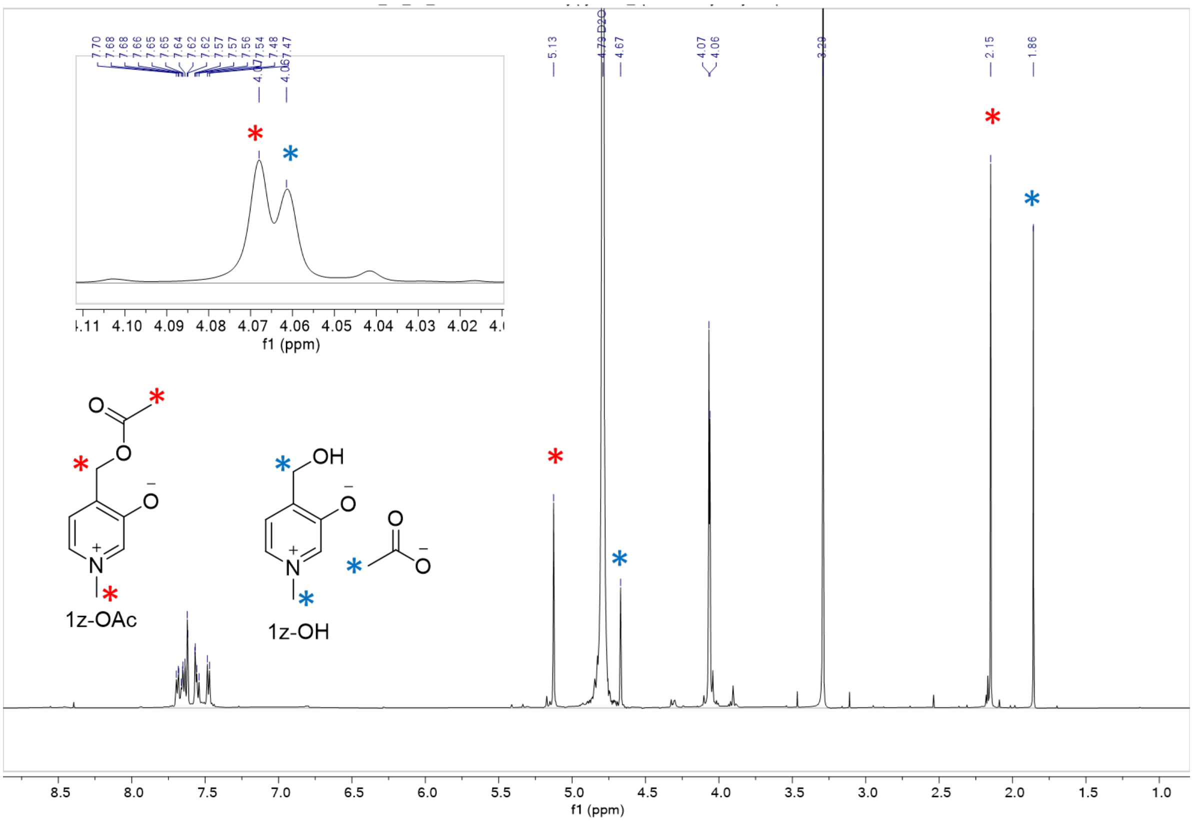

Figure S 8. NMR Spectra of photoreaction solution of **1z-OAc**.

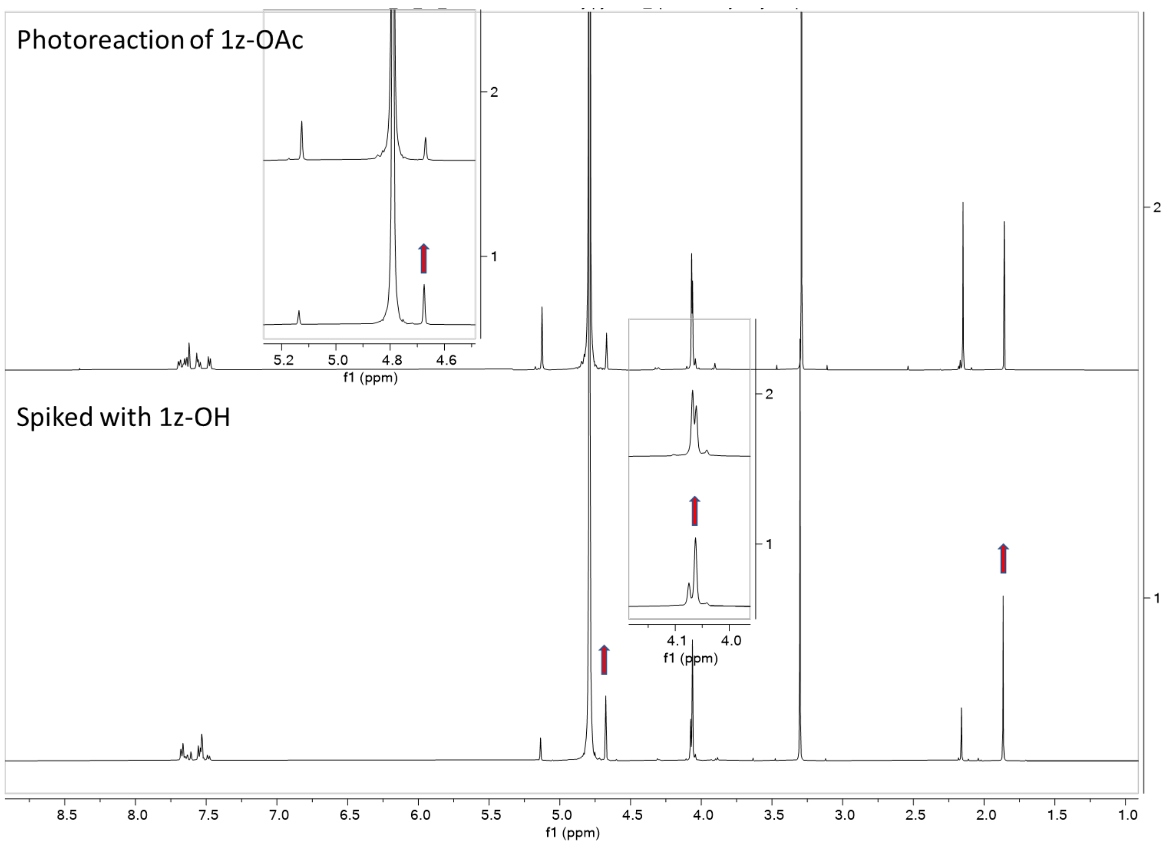

Figure S 9. NMR Spectra Comparison of photoreaction solution of **1z-OAc** and after spiking with **1z-OH**.

4 Spectroscopy studies

4.1 Absorption and emission profiles

4.1.1 Spectroscopy studies

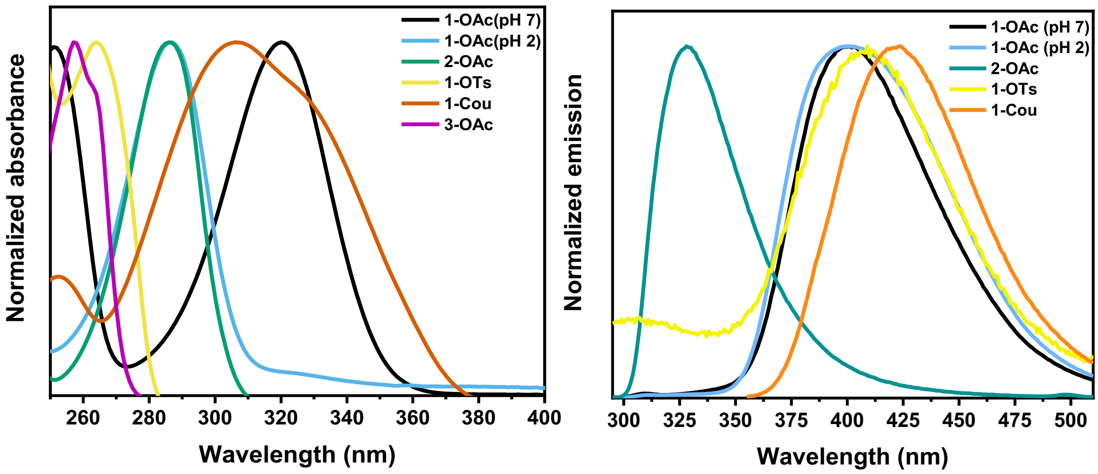

Figure S 10. (Left) Absorption spectra of the compounds synthesized in pH 7 buffer (except **1-OAc** is at pH 2). (Right) Emission spectra of the compounds synthesized, in pH 7 buffer (except **1-OAc** at pH 2)

4.2 pH dependant spectroscopy studies

A 50 mM stock solution of compound **1-OAc** was prepared using DI water. Subsequently, 20 μL from the stock solution was added to 3 mL of the respective buffer, and the absorbance was recorded.

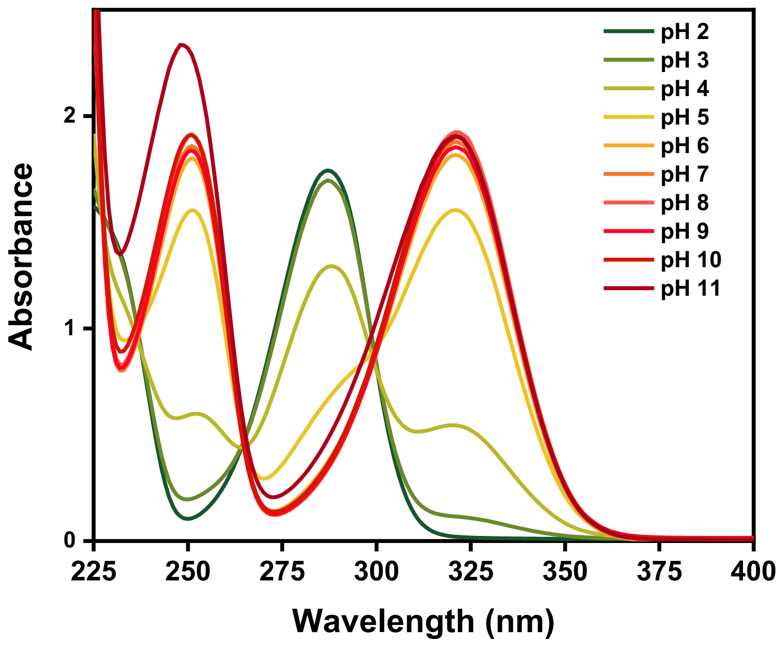

Figure S 11. Variation of absorption of compound **1-OAc** with pH.

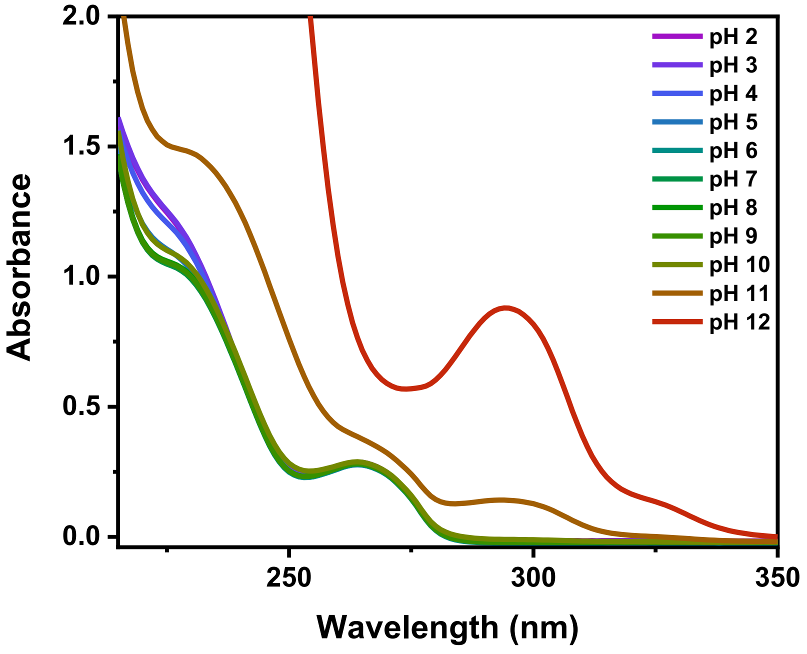

Figure S 12. Variation of absorption of compound **1-OTs** with pH.

4.3 Changes in spectroscopies with photorelease

A 3 mM solution of compound **1z-OAc** and **1-Cou** was prepared using a pH 7 buffer solution. Subsequently, 3 mL of this solution was transferred into a 4-sided quartz cuvette, which underwent irradiation with 300 nm (4 bulbs) in a Rayonet photoreactor. UV-Vis and fluorescence spectra were collected at each time interval.

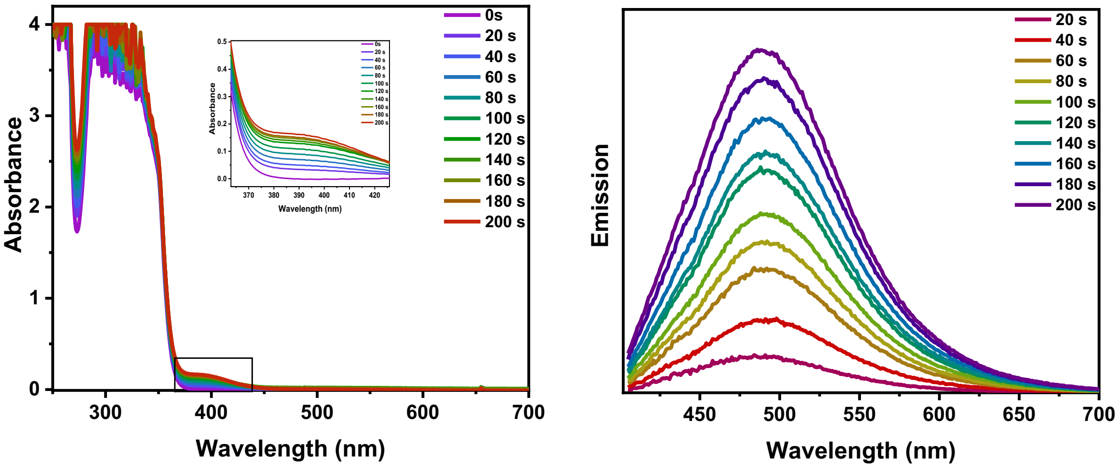

Figure S 13. Formation of **1-CHO** followed by UV-Vis (left) and Fluorescence (right), utilizing an excitation wavelength of 390 nm with a 5 nm slit width. From each timepoint, time 0 data x weightage was subtracted (0.95 to 0.77) to remove the artifacts from the solvent.

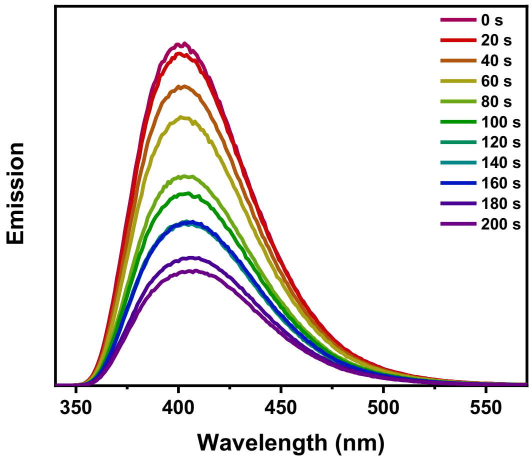

Figure S 14. Photorelease of compound **1z-OAc** followed by fluorescence, utilizing an excitation wavelength of 280 nm with a 5 nm slit width, demonstrating the depletion of the initial **1z-OAc**.

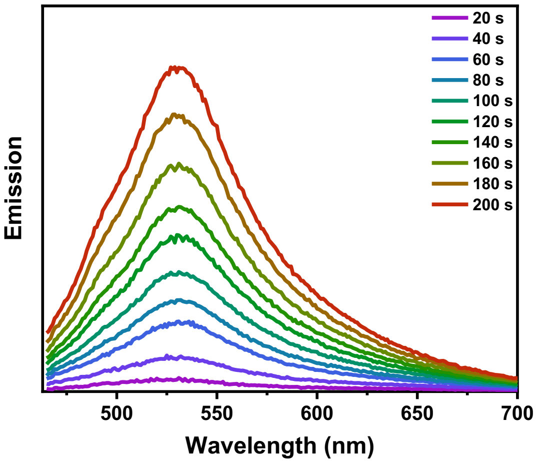

Figure S 15. Photorelease of compound **1z-OAc** followed by fluorescence, utilizing an excitation wavelength of 450 nm with a 5 nm slit width, demonstrating the formation of an unidentified photoproduct from each timepoint, time 0 data x weightage were subtracted (0.95 to 0.77) to remove the artifacts from solvent.

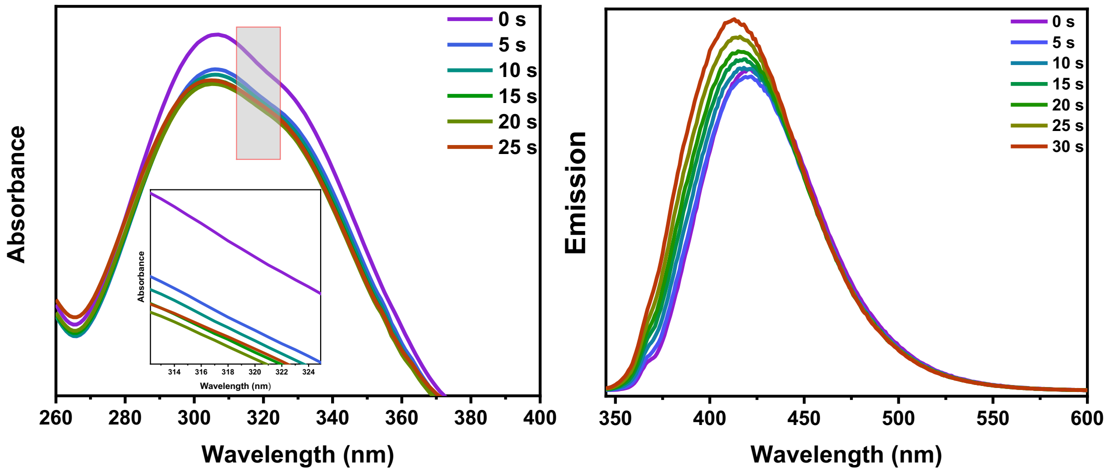

Figure S 16. Photorelease of compound **1-Cou** (3 mM) followed by UV-Vis (left) fluorescence (right), utilizing an excitation wavelength of 280 nm with a 3 nm slit width, demonstrating the release of 3-carboxy coumarin.

5 Cell studies

5.1 Cell sample preparation for fluorescence imaging

Human embryonic kidney 293 cells or HEK293 cells were grown and maintained in DMEM medium supplemented with 10% fetal bovine serum, 36.5 mM penicillin, and 12.5 mM streptomycin (Fisher Scientific, Pittsburgh, PA) in a 37 °C water jacketed CO2 incubator (ThermoScientific, Waltham, MA). Using 0.25% (w/v), trypsin-EDTA (Life Technology, Carlsbad, CA) solution, cells were subcultured every three days. For fluorescence

experiments, Nunc™ Lab-Tek™ chambered glass slides with 8 wells (ThermoScientific) were used and treated with 0.01% poly-L-lysine (Sigma Aldrich) in a sterile solution for 15 minutes. Then, the chambered glass slides were rinsed with autoclaved deionized water, followed by air drying for 30 minutes in a sterile condition. Two days prior to the experiment, the cells were subcultured in these wells. The growth media was removed on the day of the experiment, and different concentrations of compound **1z-OAc** in DMEM (serum-free) were added to the cells and incubated at 37 °C for 30 minutes. HEPES imaging medium (5 mM KCl, 155 mM NaCl, 1 mM $MgCl_2$, 2 mM $CaCl_2$, 10 mM HEPES, 2 mM NaH_2PO_4, and 10 mM Glucose, pH = 7.2) was used to rinse the cells. Finally, the cells were imaged in imaging medium.

5.2 Live cell fluorescence imaging

A Nikon Eclipse TE2000U microscope (Melville, NY) operating in wide-field, epi-fluorescence mode was used to collect all the fluorescence microscopy experiment images. Light was focused on the sample using a 100× Apo, 1.49 numerical aperture oil-immersion objective. HEK293 cells were imaged at 37±2 °C. The increase in fluorescence was seen when the cell sample was exposed to the excitation light using a mercury lamp (X-Cite 120 PC, EXFO Photonic Solutions Inc., Mississauga, Ontario, Canada) that was operated at 25% or 100% lamp power. The excitation light was filtered using an excitation filter (365±17nm) and the emitted light was filtered using an emission filter (425±25nm) from Omega Optical (XF304-1, Brattleboro, VT). A series of fluorescence images were collected with 1s acquisition time per image using an Andor iXon EM+DU-897 back-illuminated electron-multiplying charge coupled device (EMCCD) camera. The control experiments were performed with the same cells without compound **1z-OAc** or **1-OTs**. The images or movies were analyzed with ImageJ (National Institute of Health) and IGOR Pro V 6.32A (WaveMetrices Inc., Lake Oswego, OR).

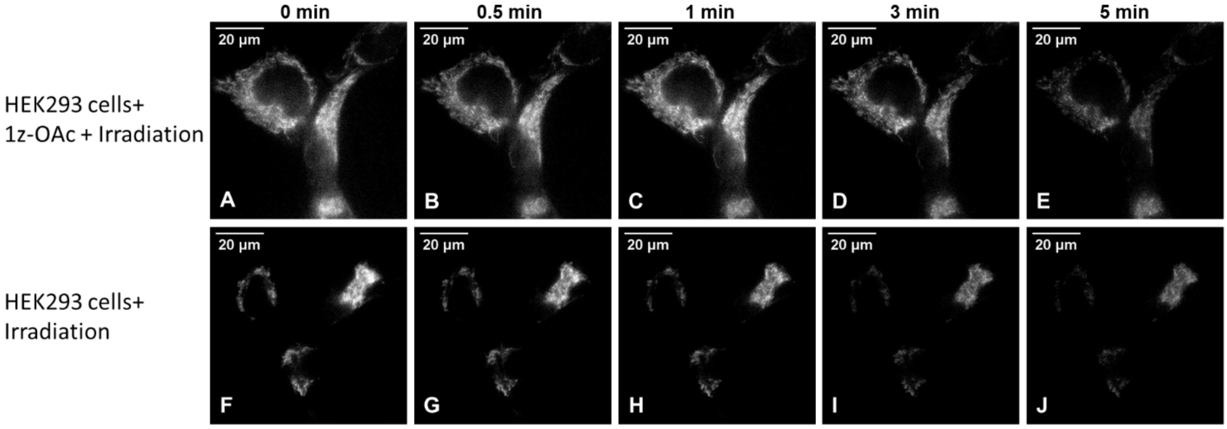

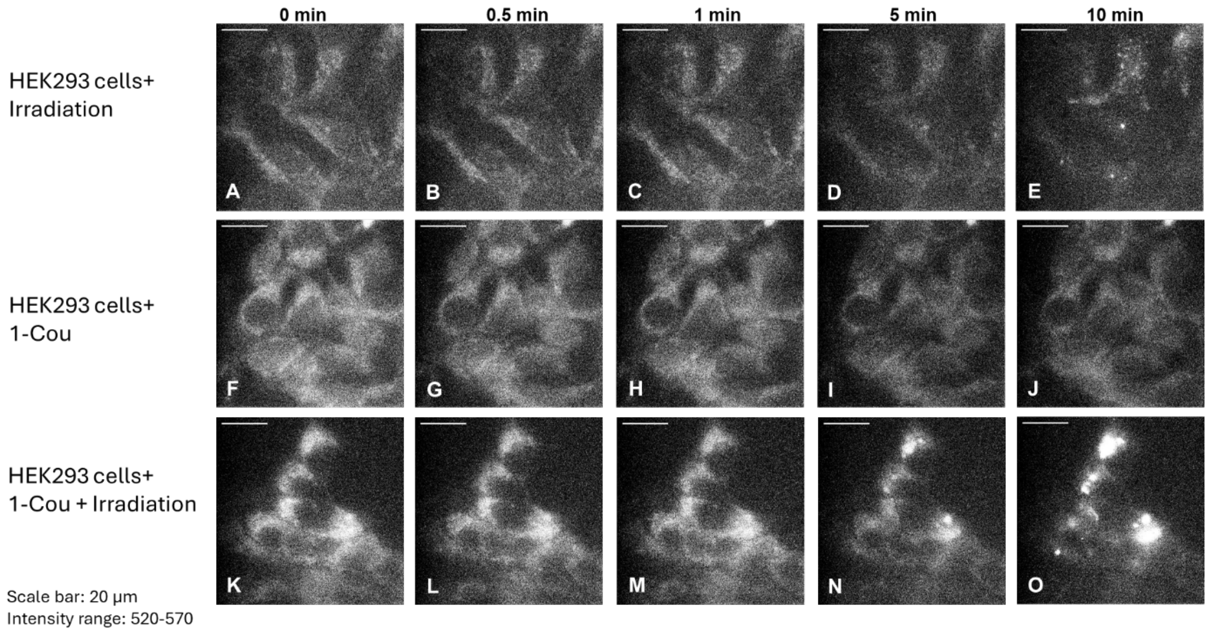

Figure S 17. HEK293 cells showing cellular uptake of compound **1z-OAc** and **1-Cou**

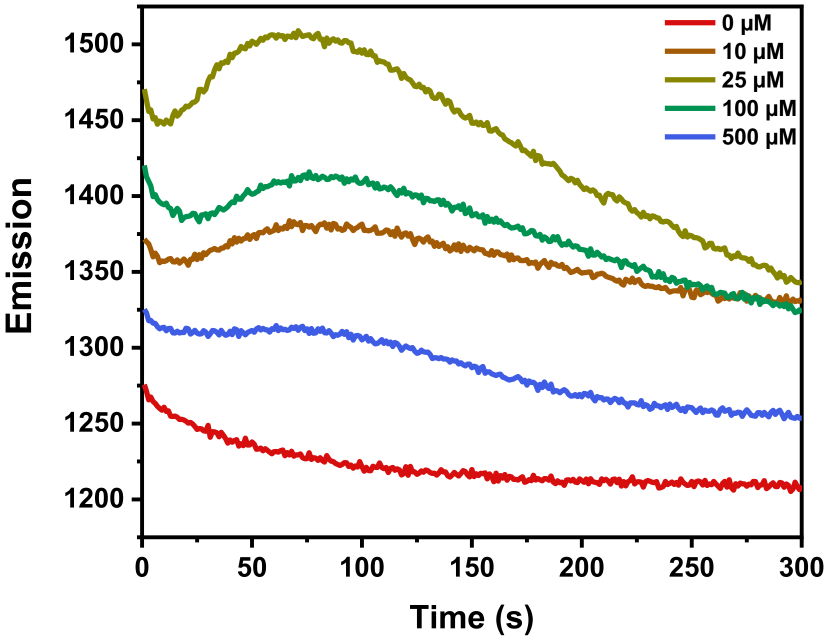

Figure S 18. Graph showing the changes in fluorescence intensity over time for live cell fluorescence imaging experiments. The change in emission of fluorescence is shown when HEK293 cells being treated with 10 µM, 25 µM, 100 µM and 500 µM of compound **1z-OAc** were irradiated continuously with 365±17 nm light. Control experiment was performed without compound **1z-OAc**.

Cytotoxicity assay

To test cytotoxicity of compound **1z-OAc** Trypan blue exclusion assays were used[3]. HEK293 cells were incubated with 0 µM, 10 µM, 25 µM, 100 µM and 500 µM of the compound **1z-OAc** for 30 minutes. Equal volumes of 0.4% trypan blue stain (ThermoScientificTM HycloneTM Trypan Blue, Waltham, MA) and the cell suspension were incubated for 3 minutes at room temperature. Using a hemacytometer (Hausser Bright-Line, Hausser scientific, Horsham, PA) and an optical microscope, the number of cells that did not absorb the dye or the viable cells was counted, and cell viability was thus calculated.

Table S2: Cell viability of compound **1z-OAc** in HEK293 cells.

Concentration of compound **1z-OAc** (µM)	Viable cell (%)
0	96 ± 3
10	95 ± 2
25	95 ± 4
100	88 ± 3
500	75 ± 5

6 Laser Flash Photolysis studies

Experimental Methods

Transient Absorption Spectroscopy

Transient absorption measurements were performed on a femtosecond regenerative amplified Ti:sapphire laser system (Spectra Physics, Solstice Ace) and an automated data acquisition system (Ultrafast Systems, Helios model). The amplifier was seeded with 86 fs laser pulses from an oscillator laser system (Spectra Physics, MaiTai). Probe pulse was obtained from the amplified 800 nm fundamental output to generate UV spectral range white light continuum (320-650 nm). For the experiments described in this study, sample solution was excited by a 320 nm pump beam, generated through optical parametric amplifier (Ultrafast systems, Apollo-T) from the amplified fundamental 800 nm. Sample solution was prepared in phosphate buffer with an absorbance 0.8 OD at the excitation wavelength of 320 nm. During the experiment, 100 mL of sample solution was flowed through 2 mm path-length cuvette with a rate of 8 ml per minute reduce any product accumulation. The data were stored as 3D wavelength-time-absorbance matrices and were exported for further use. Raw data were chirp corrected using Surface Xplorer, designed by Ultrafast systems. Data analyses were carried out in OriginPro 2022b.

Computational Methods

Geometry optimization of the possible intermediates were performed at the B3LYP level of theory and 6-31G+(d,p) basis set as implemented in the Gaussian16 programs.[2-3] Corresponding vertical excitation energies were calculated using time-dependent density functional theory (TD-DFT)[4-5] and SMD[6] solvation model in water.

Results and Discussion

Figure S 19 shows the transient absorption of compound **A** (**1z-OAc**) obtained using femtosecond transient spectroscopy using a previously described setup[1]. Compound A was dissolved in a phosphate buffer solution pH 7 was excited at 320 nm and transient spectra was collected using the visible probe from 320-650 nm[1]. At 350 nm, a fast growth is observed within the first 10 ps followed by a biexponential decay with time constants of 37.1 ps and 1.06 ns. At 400 nm, a bleach is observed. The fluorescence profile of **A** suggests that this signal is attributed to stimulated emission. Gaussian 16 calculated TDDFT/B3LYP/6-31+(g,p)/SMD=water calculations were compared to the remaining transient signals[2-6]. At 450 nm, a growth is observed within the first 50 ps followed by a monoexponential decay with a time constant of 0.94 ns. The second time constant at 350 nm is similar to this decay and likely results from the same species. Figure S-22 shows that compound **C** in Figure S-20 is the only predicted transient species to match the absorption profile in the 450 nm region. The 37.1 ps decay at 350 nm and the growth of species **C** indicate that species **C** forms from the transient species absorbing at 350 nm. Therefore, the first excited state of compound A must be the transient species responsible for the initial signal at 350 nm. It should be noted that TD-DFT absorption spectra of species **E** and **F** and **G** in have some resemblance of the long-

lived signal present. The lack of oxygen dependence demonstrated by quantum yield studies suggests that this signal is more likely due to the formation of the radical G then any transient triplet species. Since this signal appears after the formation and decay of species **C**, heterolysis appears to occur independent of the formation of radical species. Further nanosecond studies are necessary to confirm this assignment.

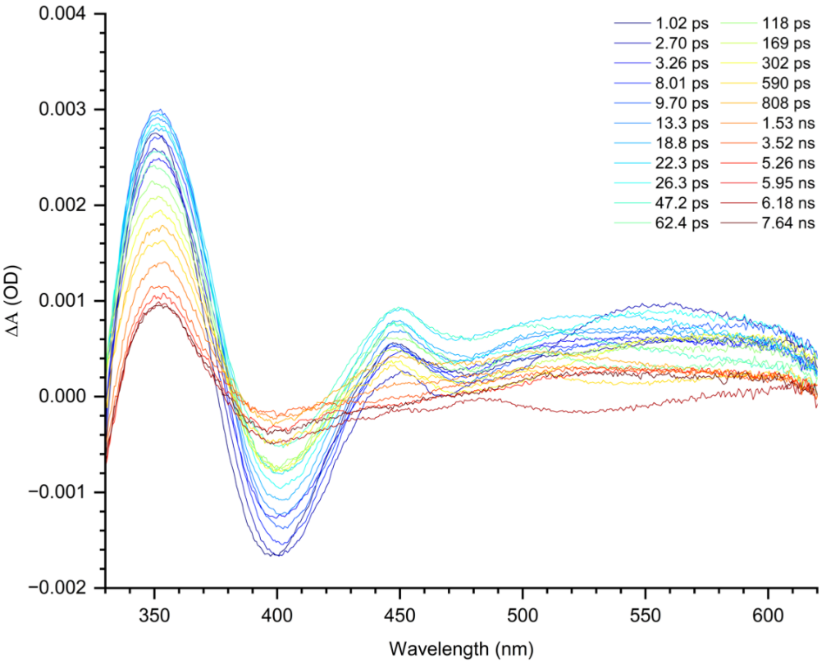

Figure S 19. Transient absorption spectra of selected time delays between 1.02 ps - 7.64 ns in phosphate buffer pH7 solution with an excitation wavelength of 320 nm. The probe range was cropped from 320-650 nm to 330-620 nm to show only transient data and not the pump excitation or its harmonics.

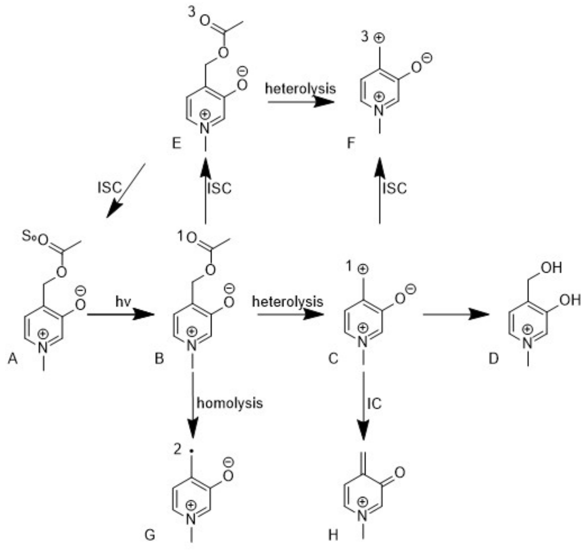

Figure S 20. Reaction Scheme of Compound A (**1z-OAc**) photolysis

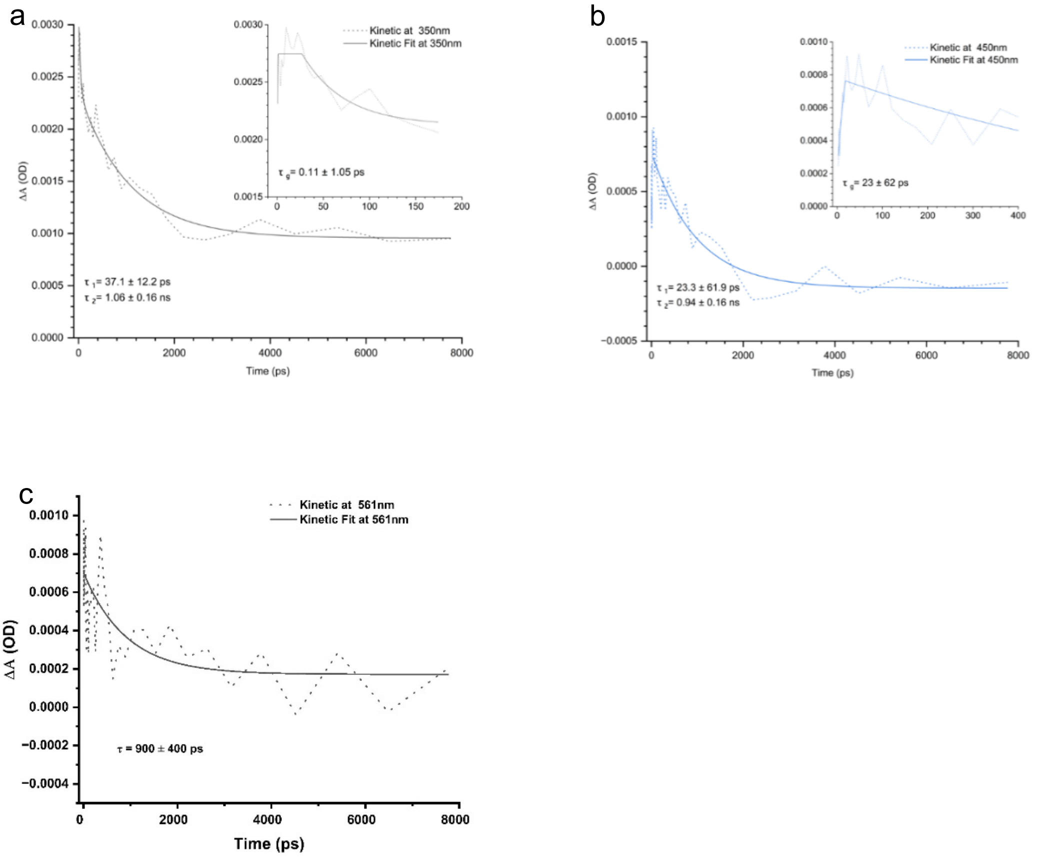

Figure S 21. Kinetic traces of compound A at (a) 350 nm (b) 450 nm (c) 561 nm and their kinetic fits. The growth of each signal is shown in the inset.

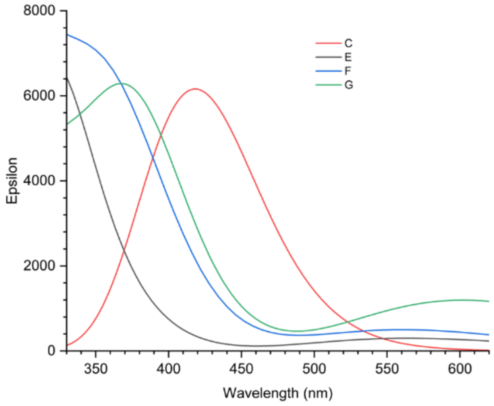

Figure S 22. TDDFT absorption spectra of transient species calculated using Gaussian16 at B3LYP/6-31+(g,p)/SMD=water level of theory[1-5]

7 DFT calculations

Computational Methods

Geometry optimization of the possible intermediates was performed at the B3LYP level of theory and 6-31G+(d,p) basis set as implemented in the Gaussian16 programs.[2-3] Corresponding vertical excitation energies were calculated using time-dependent density functional theory (TD-DFT)[4-5] and SMD[6] solvation model in water.

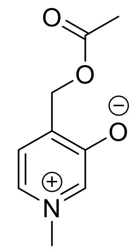

1z-OAc

0 1

C	-2.33825900	-1.37578000	-0.06938700
C	-1.02689100	-1.36911000	-0.48171100
C	-0.26676700	-0.18182300	-0.54393900
C	-0.97813700	1.06619800	-0.20628100
C	-2.33936400	0.99979400	0.20986700
N	-2.99169100	-0.21202800	0.38519600
H	-2.93255400	-2.28073400	-0.03759300
H	-0.58052500	-2.31712800	-0.76649900
H	-2.89167500	1.90359300	0.43393900
C	1.16602800	-0.16376400	-0.92531300
H	1.43121600	-1.04204400	-1.51724800
H	1.44088800	0.74324500	-1.46582400
C	4.03656000	-0.16337800	1.48212100
H	3.66706300	-0.97666000	2.11279600
H	5.10924500	-0.27041400	1.31984000
H	3.83844300	0.77945100	2.00330500
C	3.31836400	-0.15744900	0.16355200
O	3.86590500	-0.10798100	-0.93428300
O	1.98322300	-0.19980900	0.31301900
C	-4.44773500	-0.22998800	0.52274000
H	-4.75849000	0.61072400	1.14513300
H	-4.94121000	-0.15455800	-0.45513500
H	-4.75279900	-1.16013700	1.00589500
O	-0.40294800	2.19537300	-0.30184600

Sum of Electronic and Thermal Energies = -629.918036

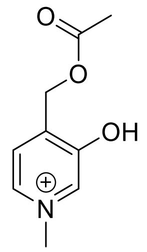

1-OAc

0 3

C	-2.39764100	-1.38652600	0.06346900
C	-1.07355400	-1.49496500	-0.30344600
C	-0.26407400	-0.37675600	-0.51801700
C	-0.94849000	0.92800900	-0.33162600
C	-2.29310300	1.01905900	0.05252800
N	-3.02167100	-0.09701200	0.26080500
H	-3.04820100	-2.23442500	0.22791900
H	-0.66336500	-2.49235000	-0.42588700
H	-2.77397600	1.98220800	0.18379600
C	1.17517900	-0.45148500	-0.87419400
H	1.42640800	-1.44961700	-1.23680300
H	1.45892400	0.29188400	-1.62193200
C	3.99872000	0.17555700	1.49024800
H	3.55989800	-0.38965000	2.31595300
H	5.06257400	-0.04543700	1.40027900
H	3.87034000	1.24223000	1.70649500
C	3.30317200	-0.14151200	0.19930100
O	3.86090700	-0.32196700	-0.87737700
O	1.96366500	-0.18954300	0.34308700
C	-4.42723200	-0.06665800	0.64819900
H	-4.74374800	0.96722600	0.78108300
H	-5.03387700	-0.54205200	-0.12988100
H	-4.56015600	-0.61713000	1.58489900
O	-0.21642000	1.99926600	-0.55812000
H	-0.70621100	2.83579300	-0.43105600

Sum of Electronic and Thermal Energies = -629.844673

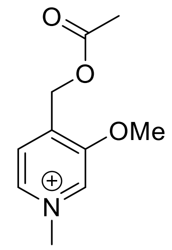

2-OAc

0 3

C	-2.25945500	-1.75025100	-0.03831100
C	-0.93778900	-1.76421100	-0.41467500
C	-0.18756800	-0.58863600	-0.54562700
C	-0.92693700	0.66572700	-0.24807800
C	-2.27287600	0.65690400	0.14414800
N	-2.94330500	-0.51304000	0.24988200
H	-2.86634100	-2.63919100	0.06703100
H	-0.47458400	-2.72539300	-0.61346200
H	-2.82024200	1.56353700	0.36409100
C	1.25263800	-0.57108600	-0.90442200
H	1.54436600	-1.52189000	-1.35344000
H	1.50587500	0.24656900	-1.58197000
C	4.06434000	-0.07324000	1.50670700
H	3.60366800	-0.66071000	2.30409900
H	5.12317000	-0.31722500	1.41519900
H	3.96376800	0.98683500	1.76651900
C	3.37346400	-0.31207400	0.19637600
O	3.93597000	-0.41078600	-0.88875800
O	2.03508600	-0.38619800	0.33261000
C	-4.33838200	-0.58244700	0.67038600
H	-4.72044800	0.42648800	0.81997500
H	-4.92852800	-1.08965500	-0.09943300
H	-4.41497600	-1.14871200	1.60463900
O	-0.22359300	1.76471200	-0.38872400
C	-0.79481300	3.08060700	-0.13781100
H	-1.12533300	3.13673900	0.90093200
H	0.01981000	3.77537100	-0.32723800
H	-1.62249900	3.24893200	-0.82930600

Sum of Electronic and Thermal Energies = -669.532922

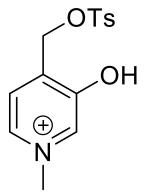

1-OTs

0 3

C	4.55979000	0.51907300	-0.33229700
C	3.31673300	0.97746200	-0.69850200
C	2.15821300	0.18136200	-0.56464100
C	2.36068300	-1.18983300	-0.05615500
C	3.67300500	-1.60797400	0.30360300
N	4.75486600	-0.74305500	0.25372000
H	5.45506100	1.11733400	-0.44906600
H	3.24460500	1.98282300	-1.10168500
H	3.85390300	-2.61746800	0.65036800
C	0.80867100	0.67600800	-0.89458600
H	0.83680000	1.47472200	-1.63599900
H	0.13558500	-0.12486500	-1.20353000
O	0.22490300	1.27691500	0.37013800
C	6.11305400	-1.27543300	0.36275400
H	6.12365100	-2.07822300	1.10189700
H	6.46347700	-1.66846300	-0.60012800
H	6.78573500	-0.48108700	0.69112300
O	1.38893300 -	2.00218000	0.04376900
S	-1.26819100	1.91551900	0.30066200
O	-1.44504800	2.53274700	1.62485900
O	-1.37623900	2.76715000	-0.89692100
C	-2.36932900	0.52571900	0.13261600
C	-2.91790400	0.22655700	-1.11622200
C	-2.64765400	-0.25663700	1.26061100
C	-3.76708600	-0.87589900	-1.22939000
H	-2.69452500	0.83945800	-1.98203500
C	-3.49567600	-1.35123200	1.12307200
H	-2.21530300	-0.01597800	2.22595300
C	-4.06979300	-1.67829600	-0.11939600
H	-4.20004700	-1.11256500	-2.19689500
H	-3.71877400	-1.96123500	1.99388700
C	-5.00027900	-2.85657900	-0.24175200
H	-5.94479800	-2.65746500	0.27891800
H	-5.22988000	-3.07580800	-1.28762000
H	-4.56346500	-3.75093900	0.21511900

Sum of Electronic and Thermal Energies = -1296.076484

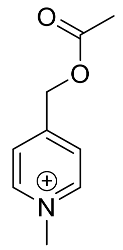

3-OAc

0 3

C	2.54127400	1.03850500	0.51774300
C	1.19555500	1.37655800	0.36669000
C	0.29234100	0.51625400	-0.21692800
C	0.83276600	-0.80485300	-0.67362700
C	2.19287600	-1.12082900	-0.51755900
N	3.03586900	-0.26591900	0.04267100
H	3.29382100	1.66396400	0.97525500
H	0.88179800	2.35268500	0.72421100
H	2.59340400	-2.07294400	-0.84766300
C	-1.13368000	0.84179000	-0.46454700
H	-1.37316900	1.85809200	-0.14547000
H	-1.39250900	0.72202800	-1.52291000
C	-3.99978300	-1.11387200	0.90165500
H	-3.91385300	-0.82868000	1.95531600
H	-5.05083600	-1.14817500	0.61545600
H	-3.54515000	-2.10199500	0.78570000
C	-3.27426200	-0.11160200	0.05428800
O	-3.79509100	0.63305400	-0.76700000
O	-1.94755300	-0.10468500	0.30394300
C	4.45288700	-0.53806900	0.22749600
H	4.69120700	-1.52346500	-0.16943700
H	5.03360600	0.23327900	-0.29007800
H	4.68899200	-0.48882100	1.29625800
H	0.18290900	-1.53477400	-1.14213800

Sum of Electronic and Thermal Energies = -555.010578

Calculation of C-O heterolysis barrier

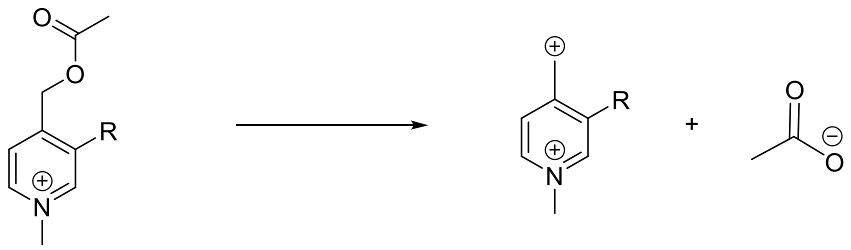

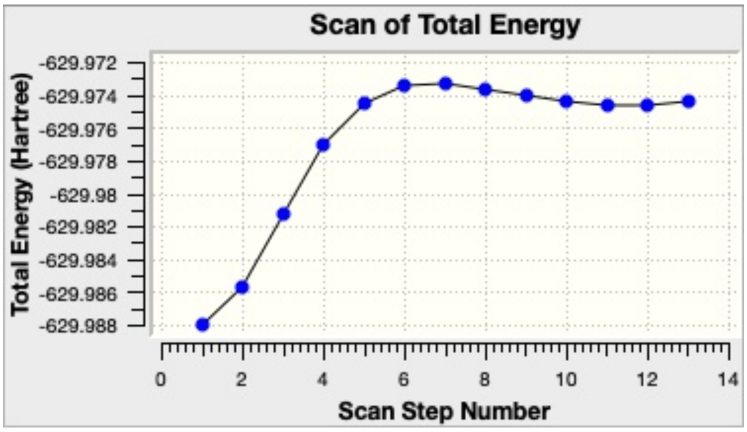

1z-OAc $\Delta E^{\ddagger}$= 9.13 kcal

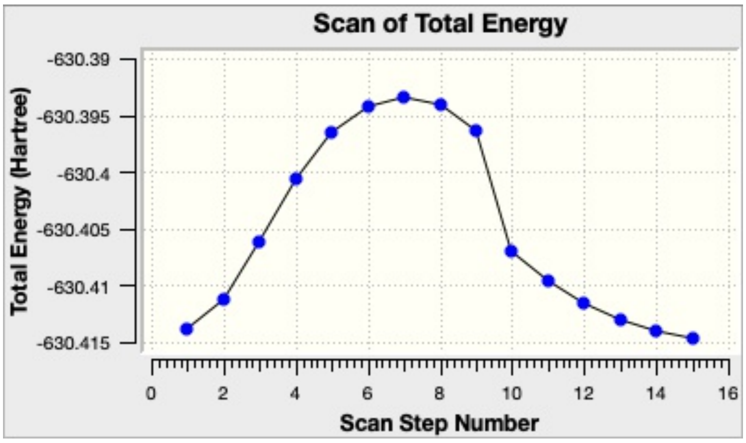

1-OAc $\Delta E^{\ddagger}$= 12.7 kcal

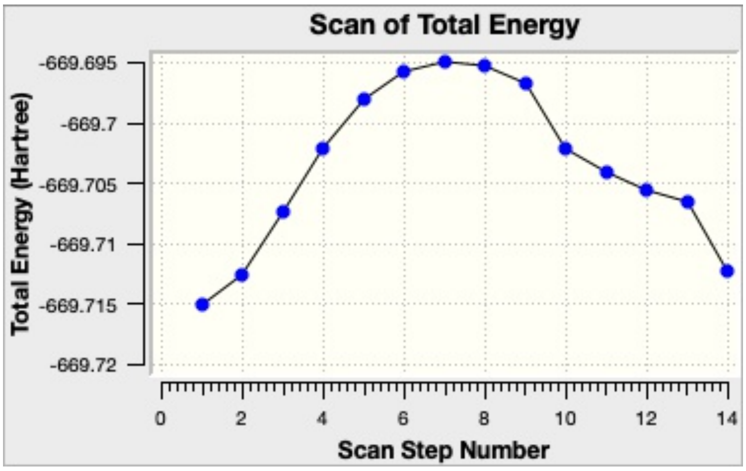

2-OAc $\Delta E^{\ddagger}$= 12.6 kcal

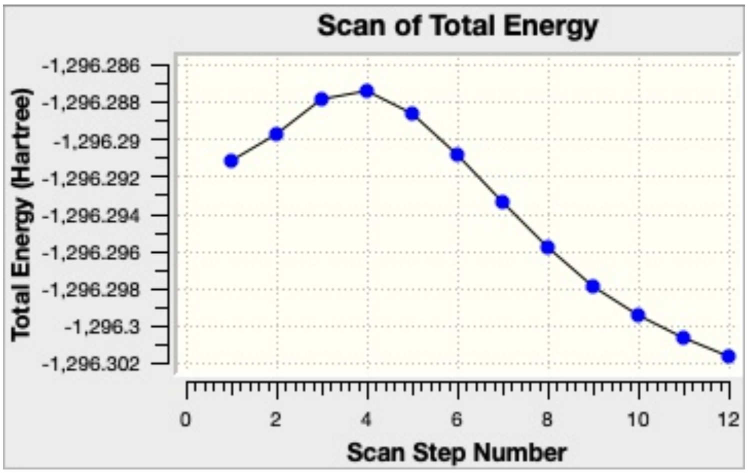

1-OTs ΔE‡= 2.4 kcal

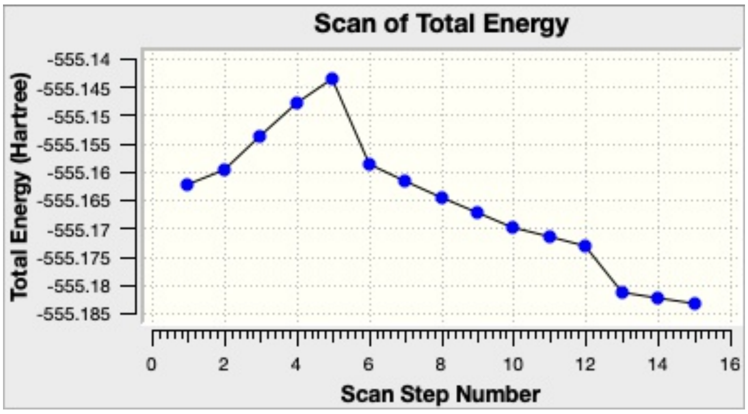

3-OAc ΔE‡= 13.4 kcal

DFT calculations for the possible reactive intermediates and their predicted UV-Vis spectra

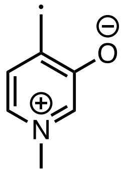

0 2

C	1.01939800	-1.28990900	-0.01123800
C	-0.31845100	-1.55747600	-0.00133300
C	-1.30530700	-0.51184700	0.00310700
C	-0.80433100	0.87778900	-0.00220300
C	0.60579400	1.04845900	-0.01181500
N	1.47032000	0.00990300	-0.01966800
H	1.78335200	-2.05657800	-0.01497900

H	-0.64345000	-2.59287200	0.00078900
H	1.03456500	2.04288000	-0.01546400
C	-2.65711400	-0.78047300	0.00995700
H	-3.38760500	0.02040400	0.01238900
H	-3.01853000	-1.80397900	0.01284500
C	2.92238700	0.25419800	0.02076700
H	3.11741100	1.29241600	-0.24354800
H	3.30116800	0.05197200	1.02602400
H	3.41675100	-0.40608900	-0.69333600
O	-1.58377000	1.89226000	0.00118800

Sum of Electronic and Thermal Energies = -401.427561

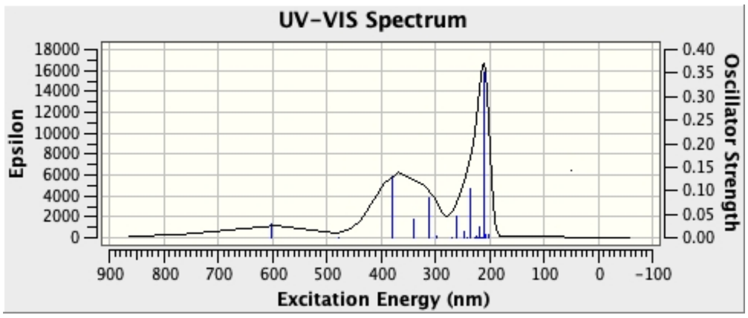

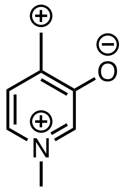

1 1

C	0.99136100	-1.29981300	0.00002400
C	-0.33114000	-1.56516400	0.00004700
C	-1.31838500	-0.50635600	0.00000100
C	-0.82379300	0.88980300	-0.00001100
C	0.64477400	1.05776100	-0.00002800
N	1.44995800	0.03169200	-0.00002700
H	1.76941700	-2.05030100	0.00002500
H	-0.66498600	-2.59695900	0.00010000
H	1.06568300	2.05691500	0.00001800
C	-2.65125300	-0.76806400	-0.00005300
H	-3.01493400	-1.79084000	-0.00011800
H	-3.37983400	0.03647600	-0.00000700

C	2.91699200	0.21998700	-0.00001300
H	3.14273600	1.28319100	0.00006300
H	3.32055200	-0.26236700	0.89202000
H	3.32060600	-0.26223400	-0.89209900
O	-1.53503500	1.89941800	0.00004800

Sum of Electronic and Thermal Energies = -401.252650

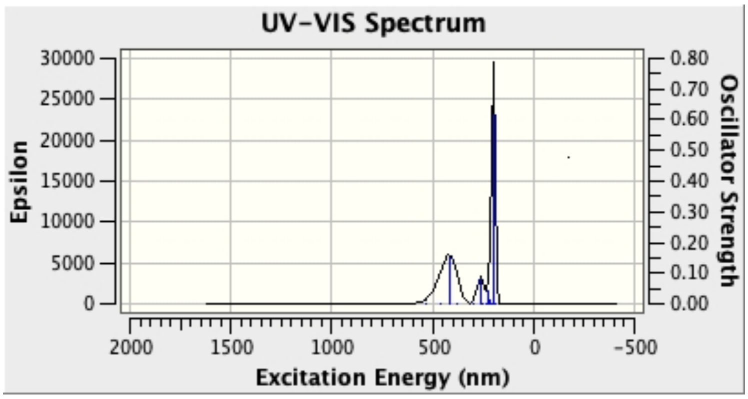

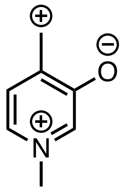

1 3

C	0.99807000	-1.28201200	-0.00000700
C	-0.38630500	-1.54027800	0.00003700
C	-1.32182300	-0.50167800	0.00001500
C	-0.78004500	0.87329100	0.00001500
C	0.63350300	1.03830700	0.00002800
N	1.49070400	-0.03165300	0.00000000
H	1.72100100	-2.08958400	-0.00005000
H	-0.70505200	-2.57632700	0.00008100
H	1.07466300	2.02770300	0.00005000
C	-2.70531000	-0.72679200	-0.00003100
H	-3.09054800	-1.74030800	-0.00008000
H	-3.40053500	0.10405000	-0.00004500
C	2.94793200	0.22367000	-0.00002500
H	3.20427400	0.79412000	-0.89412500

H	3.20434000	0.79392100	0.89418600
H	3.46915100	-0.73118300	-0.00015100
O	-1.52854400	1.89151600	-0.00000900

Sum of Electronic and Thermal Energies = -401.213907

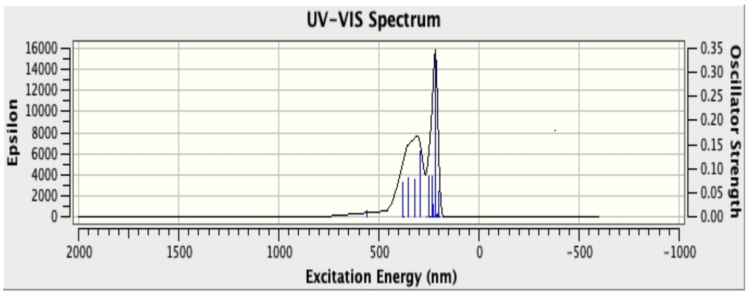

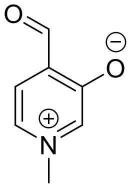

1-CHO

0 3

C	1.05578900	-1.37287900	-0.00001100
C	-0.31818500	-1.31515600	-0.00004100
C	-0.99958600	-0.08667000	-0.00001800
C	-0.25343300	1.14734600	-0.00002700
C	1.18025800	0.98960300	0.00000500
N	1.77046600	-0.20575400	0.00003300
H	1.62809900	-2.29002600	-0.00003400
H	-0.88168900	-2.24191900	-0.00005800
H	1.82086000	1.86307500	0.00000600
C	-2.46047900	-0.05009700	0.00002600
H	-2.91676100	0.95107200	0.00006300
O	-3.18354700	-1.05433500	0.00002200
C	3.24794600	-0.30405400	0.00001700
H	3.67281500	0.69709800	0.00092000
H	3.56192300	-0.84792700	0.89209200
H	3.56204900	-0.84635900	-0.89298500
O	-0.76075500	2.31767400	-0.00001500

Sum of Electronic and Thermal Energies = -476.028852

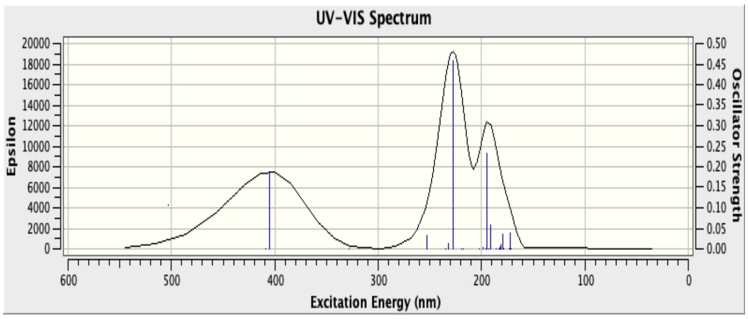

8 References

(1) Goswami, P. P.; Syed, A.; Beck, C. L.; Albright, T. R.; Mahoney, K. M.; Unash, R.; Smith, E. A.; Winter, A. H. BODIPY-Derived Photoremovable Protecting Groups Unmasked with Green Light. J. Am. Chem. Soc. 2015, 137 (11), 3783–3786.

(2) Hatchard, C. G.; Parker, C. A. A New Sensitive Chemical Actinometer. II. Potassium Ferrioxalate as a Standard Chemical Actinometer. Proc. R. Soc. Lond. Ser. Math. Phys. Sci. 1956, 235 (1203), 518–536.

(3) Strober, W. Trypan Blue Exclusion Test of Cell Viability. Curr. Protoc. Immunol. 1997, 21 (1), A.3B.1-A.3B.2.

(4).(a) Becke, A. D. Density-Functional Thermochemistry. III. The Role of Exact Exchange. J. Chem. Phys. 1993, 98, 5648−5652. (b) Lee, C.; Yang, W.; Parr, R. G. Development of the Colle-Salvetti Correlation-Energy Formula into a Functional of the Electron Density. Phys. Rev. B: Condens. Matter Mater. Phys. 1988, 37, 785−789.

(5). Frisch, M. J.; Trucks, G. W.; Schlegel, H. B.; Scuseria, G. E.; Robb, M. A.; Cheeseman, J. R.; Scalmani, G.; Barone, V.; Petersson, G. A.; Nakatsuji, H.; Li, X.; Caricato, M.; Marenich, A. V.; Bloino, J.; Janesko, B. G.; Gomperts, R.; Mennucci, B.; Hratchian, H. P.; Ortiz, J. V.; Izmaylov, A. F.; Sonnenberg, J. L.; Williams-Young, D.; Ding, F.; Lipparini, F.; Egidi, F.; Goings, J.; Peng, B.; Petrone, A.; Henderson, T.; Ranasinghe, D.; Zakrzewski, V. G.; Gao, J.; Rega, N.; Zheng, G.; Liang, W.; Hada, M.; Ehara, M.; Toyota, K.; Fukuda, R.; Hasegawa, J.; Ishida, M.; Nakajima, T.; Honda, Y.; Kitao, O.; Nakai, H.; Vreven, T.; Throssell, K.; Montgomery, J. A., Jr.; Peralta, J. E.; Ogliaro, F.; Bearpark, M. J.; Heyd, J. J.; Brothers, E. N.; Kudin, K. N.; Staroverov, V. N.; Keith, T. A.; Kobayashi, R.; Normand, J.; Raghavachari, K.; Rendell, A. P.; Burant, J. C.; Iyengar, S. S.; Tomasi, J.; Cossi, M.; Millam, J. M.; Klene, M.; Adamo, C.; Cammi, R.; Ochterski, J. W.; Martin, R. L.; Morokuma, K.; Farkas, O.; Foresman, J. B.; Fox, D. J. Gaussian 16, Rev. C.01; Gaussian Inc.: Wallingford, CT, 2016.

(6). Bauernschmitt, R.; Ahlrichs, R., Treatment of electronic excitations within the adiabatic approximation of time dependent density functional theory. Chem. Phys. Lett. 1996, 256 (4), 454-464.

(7). Stratmann, R. E.; Scuseria, G. E.; Frisch, M. J., An efficient implementation of time-dependent density-functional theory for the calculation of excitation energies of large molecules. J. Chem. Phys. 1998, 109 (19), 8218-8224.

(8). Marenich, A.V.; Cramer, C.J.; Truhlar, D.G., Universal solvation model based on solute electron density and on a continuum model of the solvent defined by the bulk dielectric constant and atomic surface tensions. J. Phys. Chem. B 2009, 113, 6378–6396.
